# Eurasian Beaver (*Castor fiber*) Winter Foraging Preferences in Northern Poland—The Role of Woody Vegetation Composition and Anthropopression Level

**DOI:** 10.3390/ani10081376

**Published:** 2020-08-08

**Authors:** Mateusz Jackowiak, Peter Busher, Dagny Krauze-Gryz

**Affiliations:** 1 Department of Forest Zoology and Wildlife Management, Institute of Forest Sciences, Warsaw University of Life Sciences, Nowoursynowska 159 St, 02-787 Warsaw, Poland; dkrauze@wl.sggw.pl; 2 Institute of Environmental Protection—National Research Institute, Krucza 5/11D St, 04-565 Warsaw, Poland; 3 College of General Studies, University of Boston, 871 Commonwealth Avenue, Boston, MA 02215, USA; pbusher@bu.edu

**Keywords:** foraging ecology, feeding behavior, beavers’ diets, human disturbance

## Abstract

**Simple Summary:**

The food preferences of beavers depend on many factors, such as forage taxonomy, stem diameter, and the distance from a riverbank. In this study, we investigated beavers’ diets and food preferences in an urban area of northern Poland subject to varying levels of human disturbance. In the course of the study, we confirmed a similar preference for the browsing of woody plants, as described in other studies. The most popular and preferred woody plants in beaver’s diet were willows and maples, and most woody plants were characterized by a stem diameter less than 10 cm. We also found that human disturbance played an important role in shaping the beavers’ diets. We discussed this phenomenon from the basis of optimal foraging theory.

**Abstract:**

We studied beavers’ dietary preferences and the role of several factors (such as plant species, size and anthropopression level) that affect the beavers’ foraging in northern Poland. Woody plants along the river were measured and classified according to species in six 100 m-long transects that were characterized by a diversified human disturbance level. Ivlev’s electivity index was used to present the beavers’ preferences for various plant species and sizes, and the generalized linear model was used to assess the significance of studied factors in beavers’ browsing choices. Most popular in the beavers’ diets were willows (*Salix)*, maples (*Acer)* and alder (*Alnus)*, but only willows and maples were preferred. We noted a decrease in the beavers’ foraging preference in parallel to an increase in the shoot diameter; plants with a diameter below 10 cm were preferred. All factors included in the generalized linear model (GLM) were significant in shaping the beavers’ foraging choices. A negative correlation between the shoot diameter and the human disturbance level was found, but the species composition of the browsed woody plants was the same in each transect. Beavers’ foraging preferences, as observed in our study, were similar to those described in the literature and confirmed the role of woody species and their diameters in shaping the beavers’ diet. We also suggested the potential role of anthropopression in the shaping of the beavers’ foraging behaviors.

## 1. Introduction

The Eurasian beaver (*Castor fiber*) and the North American beaver (*Castor canadensis*) are examples of strictly herbivorous rodents that use many herbaceous and woody plant species for food [1,2,3,4]. As a consequence of their diverse diet, they are considered foraging generalists, and many publications have documented their foraging preferences [5,6,7,8,9,10,11]. Beaver foraging preferences are often explained in the context of optimal foraging theory (OFT) [12,13,14]. According to the theory, foraging animals tend to maximize their energy gains by choosing the food resources with the highest energy content and by minimizing energetic expenditure during foraging. Beavers are considered central-place foragers, meaning that foraging intensity decreases with increasing distance from a central place. For beavers, the central place is typically associated with a water source. Beavers either forage along a watercourse (stream, river or pond) bank or, if farther away, will bring their food back to the bank for consumption [8,15,16,17,18]. The maximum distance for foraging from water generally ranges between 20 and 40 m [8,19,20,21,22], and typically does not exceed 60 m [18,23,24,25].

The food preferences and foraging choices are shaped by many factors: the forage taxonomy, shoot diameter, distance from the riverbank [26], patchy forage distribution [27], plant secondary metabolites content [24,28,29] and predation risk [30,31]. Beavers also use annual or perennial aquatic and terrestrial herbaceous plant species [6,32,33].

The use of woody species is especially prevalent at northern latitudes during autumn, which is when beavers construct a winter food cache [1,3,4,34,35]. The beaver foraging preferences of woody plant species indicate that they prefer soft-wood species. In most of the studies, willows (*Salix)*, hazels (*Corylus*), birches (*Betula*) and poplars (*Populus*), especially the quaking aspen *Populus tremula* in Europe, are preferred by beavers [9,10,35,36,37]. The dominance of willow species in beaver diets has been reported for populations in the Czech Republic [38], Norway [9] and Poland [37], as well as in North America [19]. Besides willow, hazel has been reported as a preferred tree in Europe [10,36,37,39] while aspen is often considered a preferred species in North America [5,19,40,41]. The oak (*Fagus*) [36] and ash (*Fraxinus*) species [42] are reported to be in the beaver diet, but usually, these species are of less importance [37]. A strong preference for the alder (*Alnus*) species has been documented in both Europe and North America [5,36,40], but this preference may be very site specific [43,44]. When available, maple (*Acer*) species have been reported in the beaver diet [9,37,40], and in North America, they have been considered a preferred species [25] and to be important components of the winter food cache [34]. The variability in beaver foraging preferences within a genus has also been reported [27].

Beaver foraging preferences are influenced by the diameter of browsed plants, regardless of genus/species preferences. Beavers have been reported to utilize only the parts of plants that have the highest nutritional value, which tend to be in the youngest developmental phases and characterized by the smallest shoot diameters with the highest concentration of nutrients [20]. Beavers usually browse trees with relatively small diameters (1 to 15 cm); trees in this size range can account for up to 90% of the total of cut trees [9,36,37].

Beavers are not only food generalists but are relatively adaptable in their habitat selection. The factors influencing habitat utilization by beavers, excluding food availability, include the river/stream depth, the bank profile, the distance to roads, and other human activities and disturbances [45,46,47]. Webb et al. [48], South et al. [49] and Swinnen et al. [50] have documented the ability of beavers to occupy wetland areas associated with urban areas, but this is dependent on the availability of their preferred food species [38,51]. However, no information currently exists as to if or how beaver behavior will change under high levels of anthropopression. Swinnen et al. [52] suggested that beavers maintain their nocturnal activity in habitats with no natural predation because of human pressure/disturbance. Additionally, Czyżowski et al. [53] compared beaver diets in urban and suburban/rural habitats and found differences in the diameters of the browsed plants between the habitats. However, it was not clear if anthropopression was the reason for the observed differences in foraging. Given the increasing range expansion and population growth of the European beaver in the anthropogenic European landscape, it is important to understand if and how anthropopression influences beaver foraging behavior.

This study describes the impact of woody plant species and their diameter on beaver foraging preferences. In this study, we also described the general relationship between the beaver foraging choices and the level of human disturbance, and tried to find out if and how anthropopression may have influenced the selection of forage species.

## 2. Materials and Methods

### 2.1. Study Area

The study was conducted on the Gwda River near the city of Piła (Piła county, Greater Poland voivodeship; 53°09′05″ N, 16°44′16″ E). This area is located in the macroregion of the Southern Pomeranian Lakeland in the physico-geographical mezoregion of the Gwda Valley. The climate is relatively dry with an average annual rainfall of 500–550 mm. The city is surrounded by coniferous forests with low-nutrient, mineral sandur soil and dominated by Scots pine (*Pinus sylvestris*). Piła’s municipality, with a population of 73,987 and a population density of 720 people/km^2^ (in 2016), encompasses 102.68 km^2^ with a forest area covering 53.32 km^2^. The highest percentage of green (open) space is in the western and southern parts of the city.

The Gwda River is 145 km long, and the basin surface is 4943 km^2^. It is a typical meandering river with numerous oxbow lakes. The natural river regime is weak due to the waterflow and water level regulation by six small hydroelectric power plants.

The total length of the river in the city area is 20 km, and the average depth is approximately 2 m. The Gwda flows in a north–south direction through the city with an average waterflow of 27.2 m^3^/s, reaching a high of 60 m^3^/s during a water short accumulation period (mostly occurring between spring and autumn). For the habitat characterization, the river in Piła can be divided into three sections: northern, central and southern. The northern and southern sections possess a more natural character with more meanders, a wider riverbed (average 35–50 m or more), much more vegetation and steeper riverbanks than the more urbanized central section. However, the southern section is narrower and has more gradual, lower-angled banks than the northern section. The urban central section is more human-modified, with a very narrow riverbed (20–30 m), fewer meanders, with banks reinforced by concrete slabs, less abundant vegetation, and an artificial island in the town center. There is also an oxbow lake and an artificial riverbed present, due to a river course regulation in the town area. Tributaries are present; the largest one connects the river with Zalew Koszycki, an artificial lake. Most tributaries are small and drain water from the wetlands and marshy areas to the river.

In the city area, the riverbanks are mostly covered with black alder (*Alnus glutinosa*), black poplar (*Populus nigra*) and some willow species (*Salix* spp.). Additionally, maples (*Acer* spp.), including the Norway maple (*A. platanoides)* and ashleaf maple (*A. negundo)*, are present. The most frequent shrub is the European elder (*Sambucus nigra*).

Beavers have been present in the study area for at least 15 years, and there are between 4 and 7 active sites on the river in the city area [54]. The Eurasian beaver is a protected species in Poland, and animals are not persecuted in any way within the study area.

### 2.2. Field Studies and Data Analysis

The study was conducted from the 5th to 15th of April 2017. We established six 100 m long transects along the river in the city area. The transects were distributed randomly along the river and were not placed within the specific beaver site. The transects were relatively homogeneous in terms of the width of the riverbed (about 25–35 m); only the two located nearest to the town boundary were wider (about 50–60 m). The banks of the river were high (about 1 to 1.5 m) and steep on transects II, III and IV, while low with a gentle slope on transect I. For the two transects located the closest to the town center (V and VI), the banks were of medium height, with slopes of about 45 degrees, reinforced by concrete slabs. The woody vegetation composition was natural and, in general, not altered by humans. However, there were few introduced tree species and a narrow, mowed lawn in transect VI. In transect VI, and partially in transect V, there was a 2 m wide walking path.

We measured the anthropopression level in each transect by determining the average distance from the asphalt roads and buildings, the vehicle-traffic intensity, the asphalt and dirt roads nearest to the river, the pedestrian and bicycle traffic intensity near the river, and the distance to the town center (Table 1). We selected variables based on those suggested by Baker et al. [47]. The intensity of each variable was measured on a three-point scale. The average distance from asphalt roads and buildings was determined by measuring the distance between the transect center point and the nearest roads and buildings in each geographical direction. The pedestrian, bicycle and vehicle traffic intensity was determined by counting each pedestrian, bicycle and vehicle on the nearest asphalt or dirt road or path near the transect center point, and in each transect at 12 a.m. for one hour. Although beavers engage in nocturnal activity, it was assumed that the traffic measured at midday would be proportional to the traffic in the evening and in the morning―when beavers are mostly active. This parameter was tested once for each transect on a working day, under similar weather conditions. 

An inventory of woody plant species was conducted in each transect, at one of the river banks. In each case, the riverside with richer woody vegetation was selected, while the other side was mostly dominated by herbaceous plants, and held few woody plants. We measured every cut and uncut woody plant in each transect up to 30 m from the river bank, and we divided all of the measured woody plants into three distance zones: 0–10, 10–20 and 20–30 m. The trees and shrubs were classified according to species when possible. The diameter was measured at a height of 30–40 cm, where most of the browsing signs are usually present [39], and any beaver browsing signs were noted. If a plant branched out below 30–40 cm in height, each branch was measured as a single plant. For the diameter measurements, a forestry caliper was used with a measuring range between 0 and 80 cm and a measuring resolution of 0.1 cm. For the foraging preferences analysis, we grouped inventoried woody plants by genus. The beaver foraging preferences were defined by Ivlev’s electivity index with Jacob’s modification [55] by using the formula:

(1) $$E=\ln\left[\frac{r\left(1-p\right)}{p\left(1-r\right)}\right],$$

where r is the mean share of the genus among the woody plants browsed by beavers, and p is the mean share of the genus among the woody plants available for beavers (browsed and not browsed in total).

The electivity index was counted for the respective genus for all the transects in total and for the respective genus among three diameter classes: below 2.5 cm, 2.5–10 cm and above 10 cm.

### 2.3. Statistical Analysis

For testing the normality of distribution, Shapiro–Wilk’s test was used. Differences between the shoot diameters on the respective transects were confirmed by the Kruskal–Wallis test. To test the influence of other factors on the probability of tree browsing, a generalized linear model (GLM) with a binomial distribution was used. The optimal model was built from the best subset selection and was selected on the basis of the lowest Akaike information criterion (AIC) value. The dependent variable was the browsing of tree or shrub by beavers (classified as browsed or unbrowsed), and the independent variables were the plant diameter (the continuous variable), the plant species and the human disturbance level (the categorical variable). Moreover, Spearman’s rank correlation coefficient was used to examine the dependence of the shoot diameter and the anthropopression level. All differences and models were statistically significant at the significance level *p* < 0.05. Statistical analysis was performed using the Statistica software, version 13.1 [56].

## 3. Results

The transects were characterized by different levels of human disturbance. The human disturbance level increased according to the intensity of urbanization from the city borders to the city center (Table 2).

**Figure 1:**
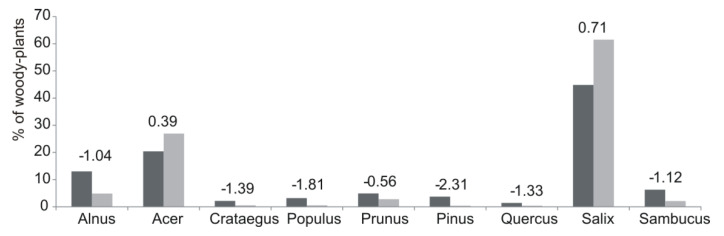
Woody plant genera available (in dark gray) and browsed by beavers (in light gray) with the electivity values (Ivlev’s electivity index with Jacob’s modification) in all the transects located along the Gwda River. Only the genera with an abundance above 0.5% are included in the figure.

A total of 1931 woody plants, belonging to 20 genera and 26 species, were inventoried in all of the transects (Table S1). The majority of the trees and shrubs belonged to nine genera (Figure 1). On five of the six transects (all except transect I, which was the farthest from the town center), the number of species was similar (10–13 species). The highest species richness was observed in the centrally located fifth transect, and the lowest, in the most peripherally located (near the town border) transect (I) and in the city center (VI). The greatest abundance of the woody plants was found in the semi-centrally located third and fourth transects (Table 3).

The average diameters of the browsed and unbrowsed plants differed among transects (in total) (H = 414.304 *p* < 0.001), as did the average diameter of only browsed plants (H = 199.056 *p* < 0.001). The largest average plant diameter was observed in the peripherally located transect I (19.1 cm), and the smallest, in the semi-centrally located transect III (2.3 cm). For the browsed plants, the largest average diameter was in the peripheral transects I and II (about 11 and 6 cm, respectively). In the other four transects, the average diameter of the browsed plants did not exceed 2.3 cm (Table 3). The most abundant woody plants across all the transects were willow (*Salix)*, represented mostly by the white willow, *Salix alba*, which accounted for almost 50% of the trees and shrubs; maple (*Acer)*, mainly the ash-leaf maple, *Acer negundo* (introduced from North America), and the native Norway maple, *Acer platanoides* (12% and 8%, respectively); alder (*Alnus)*, but only the black alder, *Alnus glutinosa* (13%); and poplar (*Populus)*, mostly the black poplar, *Populus nigra* (3%) (Figure 1). The genera that were occasionally found were fir (*Abies*), birch (*Betula*), hazel (*Corylus*), hornbeam (*Carpinus*), ash (*Fraxinus*), juniper (*Juniperus*), walnut (*Juglans*), crabapple (*Malus*), spruce (*Picea*), linden (*Tilia*), and thuja (*Thuja)*. Due to low abundances, these are not reported in Figure 1. Most of the inventoried plants (81%) across all the transects were from within 10 m of the riverbank.

The willow (61.1%) and maple (26.8%) were browsed most heavily by the beavers, while alder (4.8%), blackthorn (2.8%) and black elder (2.1%) were utilized less. Every other genus accounted for less than 1% of the plants utilized by beavers (Figure 1). Only *Salix* (E = 0.71) and *Acer* (E = 0.39) had positive electivity values, indicating selection was greater than their availability. The occurrence of alien maple species (mostly *Acer negundo*) and native species (mostly *Acer platanoides*) in the beavers’ diets was similar (16.1% and 10.7%, respectively), and the beavers preferred both species (E = 0.38 and E = 0.32, respectively). All other genera were used at lower intensities or sometimes avoided by animals. Beavers also used *Prunus* where available but still exhibited a negative electivity (E = −0.57) for this genus. *Alnus* (represented only by black alder trees) was readily available but had a negative electivity (E = −1.04), indicating a low preference. All other genera had lower availabilities, were rarely used as forage by beavers and were characterized by negative electivity values (Figure 1). 

**Figure 2:**
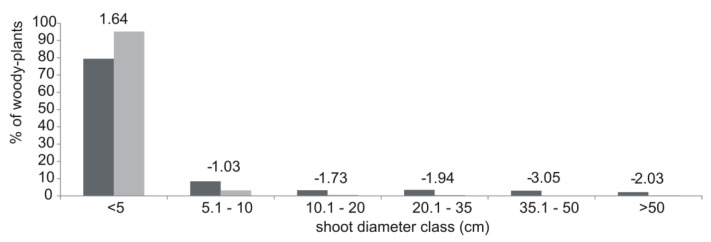
Woody plant diameters available (in dark gray) and browsed by beavers ( in light gray) with the electivity values (Ivlev’s electivity indice with Jacob’s modification) in all the transects located along the Gwda River.

The majority of the woody plants in the transects were less than 10 cm in diameter (87.9%), and 95.2% of all the shoots browsed by beavers were less than 5 cm in diameter (Figure 2). Preference was noted for the plants with diameters not exceeding 5 cm (E = 1.64), and the trees and shrubs with diameters greater than 5 cm were generally avoided (negative electivity indices). In all transects, the majority of the browsed plants (98%) were located within 10 m of the riverbank. 

A decreasing browsing preference with an increasing shoot diameter was noted for *Alnus*, *Acer*, *Prunus*, *Salix* and *Sambucus*. This was not observed for other genera. For some genera (*Pinus* and *Quercus*), it was impossible to determine diameter preferences due to the absence of some diameter classes (Table 4). The observed beavers’ foraging preferences, in relation to the woody plant diameter, were similar on each transect (Table S2).

The GLM was statistically significant (W = 4.0433 *p* = 0.044). All factors included in the model were also significant (Table 5). The factor with the highest impact on beaver foraging choices was genus, while two factors (shoot diameter and anthropopression level) had a weaker influence. A weak negative correlation between the browsed plant diameter and the anthropopression level was found (r = −0.1348 *p* < 0.001).

## 4. Discussion

The availability of woody forage species is critical in determining beaver habitat quality [10,38]. The food availability in the study area was high and included the preferred species. Willow (*Salix* spp.) is well documented as a vital component of and the preferred food in both beaver species’ diets [9,10,19,38,51], and it was the most dominant species in the study area. Maple (*Acer* spp.) was the second most dominant tree genus in our study area, and various maple species have been noted as important beaver food species [9,25,34]. The percentage of alder was similar to that reported in other studies [9,10,25,36]. It should be emphasized that the presence of some species among the browsed plants does not necessarily have to be related to the choice of food, as theycan be used for building purposes, such as in the case of alder [57,58].

Czyżowski et al. [53], working in the urban area of Lublin in south-eastern Poland, documented that white willow (*Salix alba*) was the most favored species, with maples and alder also being important food species. They reported no difference in browsed species selection between urban and suburban areas, which is similar to in our study.

The importance of ash-leaf maple, which was the most dominant *Acer* species in the study area, in the Eurasian beaver’s diet is an interesting observation since, in the natural range of this plant species (North America), it is not considered a major component of the beaver diet [59,60]. Ash, hazel and oak were not important components of the beavers’ diets, most likely due to their rare occurrence and irregular distribution in the study area. Two genera (*Salix* and *Acer*), which were the two most available genera, had positive electivity indices, indicating that the beavers utilized these plants more than was suggested by their availability. The electivity indices for all other genera were negative, but those were generally uncommon (other than *Alnus*) in the transects. It is difficult to define the genera preference because beavers appear to utilize the most available and palatable species. Belovsky [20] also reports that it is difficult to assign preference because beavers forage in a manner that maximizes energy intake and minimizes foraging time.

Our observation that 98% of the browsed plants were located close to the riverbank is similar to that reported in other studies [23,25,27,40]. We observed that the foraging intensity decreased with increasing distance from the river, which supports the central-place foraging (CPF) hypothesis. The central-place foraging theory suggests that not only should foraging intensity decrease with increasing distance from the central place but the average diameter of the browsed trees should decrease too [26]. However, this was impossible to test in our study area because the browsed plants were not recorded at all distances from the riverbank. This was especially true in transects close to the urban center.

The preference for the small diameter plants (less than or equal to 10 cm in diameter) has been well documented, with studies reporting that these diameters make up approximately 90% of all the browsed plants [9,37]. In our study, 95% of browsed plants were 5 cm or less in diameter, supporting a general preference for the smaller diameter plants. However, a preference for the larger diameter plants (10–30 cm) has been observed in Scotland [10]. In that study, the browsed plants belonged to a preferred genus (*Salix*, *Alnus* or *Populus*). Additionally, Urban et al. [42] reported that the diameter selection may be species dependent, with beavers preferring the smaller diameter ash and the larger diameter willow. The general pattern appears to be that trees belonging to the preferred genera are browsed more heavily, regardless of their diameter, possibly due to the physio-chemical wood and tissue properties of these species. However, Jenkins [61] clearly found that the beaver foraging intensity on plants of the preferred genera decreased with increasing shoot diameter and distance from the bank. Based on our study, we conclude that it is the combined influence of the browsed plant diameter and the genus (species) that is more important in shaping the beaver foraging pressure, compared with either of these factors considered separately.

The selection of browsed plants did not change with the anthropopression level, but we did observe a decreasing average diameter of the browsed plants, which was associated with the increasing anthropopression level. However, this association was not entirely clear because there was little variance in plant size across the two semi-central transects.

The smaller diameters of the browsed trees in a landscape with higher anthropopression may be explained in two ways. The first explanation regards the overall habitat quality (measured as food availability). It has been shown that beavers, as generalist herbivores, change their food selectivity based on food availability (habitat quality). For example, in lower quality habitats, the beaver selectivity patterns are not clearly observed (they eat what is available), compared to in the higher quality habitats where food preferences are more visible [62]. However, this explanation excludes the human disturbance level as a significant factor in shaping the beaver diet. Due to additional variables, it can also be more difficult to classify urban habitats as high or low quality.

The second explanation is derived from the optimal foraging theory, and the reaction of beavers to a “predatory” presence (such as a human or dog presence). The higher the predation risk or human presence/disturbance, the more the beavers may decrease their foraging pressure on specific plants [30,31,63]. However, only a few studies have confirmed the influence of the human or dog smell on beaver foraging activity [30,63], or beaver sensitivity to anthropogenic smell; the importance of direct contact with humans in shaping behavior has also been reported [64]. It was also postulated that human presence can force a beaver’s nocturnal activity [52]. Based on our study, we suggest that to optimize foraging and minimize human contact/disturbance in urban areas, beavers should choose more easily available foods with high energetic value and smaller diameters, closer to the riverbank.

## 5. Conclusions

The presented results confirmed the observations made during other studies on beavers’ feeding preferences. Regardless of the habitat type and the degree of human disturbance, beavers tend to choose the same deciduous woody plant species with soft wood, such as willows and maples. However, some species can be characterized by a high percentage of occurrence in a beaver’s diet composition, but this is probably due to their wide availability rather than a high preference for this species. Additionally, a clear preference for small-diameter plants, seems to be a constant and independent of any habitat characteristics. Two factors play a major role in shaping beavers’ foraging preferences―plant species and size. That said, other factors that impact beavers’ diets can be important. The results of our GLM analysis indicated that, in general, anthropopression may be potentially significant in determining beavers’ foraging choices, which was probably observed in the decreasing diameter of the browsed woody plants with a rising anthropopression level.

## Figures and Tables

**Table 1:** Method of anthropopression assessment in woody plant species inventory transects, along the Gwda River.

Variable	Number of Points Assigned		
	1	2	3
Average distance from roads (m)	>301	151–300	0–150
Number of vehicles (N/h)	<20	21–100	>100
Number of pedestrians and bicycles (N/h)	<50	51–100	>101
Distance from the town centre (km)	>2.5	1–2.5	<1

**Table 2:** The atheroprogression level (N of points assigned) for the six transects, located along the Gwda River banks, with the general location in relation to the central urban area (in brackets).

Variable	Transect					
	I	II	III	IV	V	VI
	(Peripherally Located)		(Semi-Centrally Located)		(Centrally Located)	
Average distance from roads (m)	1	2	1	2	2	3
Number of vehicles (N/day)	1	1	1	3	2	3
Number of pedestrians and Bicycles (N/day)	1	1	2	2	3	3
Distance from town centre (km)	1	1	2	2	3	3
Total	4	5	6	9	10	12

**Table 3:** Descriptive statistics and characteristics for the woody plants in transects with different human disturbance levels, inventoried for the beavers’ (*Castor fiber)* foraging preference assessment on Gwda River.

Parameter		Transect					
		I	II	III	IV	V	VI
Number of woody plants	Total	151	274	661	437	287	125
	Browsed	22	75	576	373	214	82
	Unbrowsed	129	199	85	64	69	43
Percentage (%) of plants located within 10 m from the riverbank	Total	38.4	47.1	95.6	93.1	86.1	68.0
	Browsed	63.6	92.0	100.0	98.9	96.3	100.0
Number of species	Total	7	11	11	10	13	8
	Browsed	6	8	7	8	8	1
	Unbrowsed	1	3	4	2	5	7
Mean (±SD) diameter of woody plants (cm)	Total	19.1 (±20.3)	8.4 (±11.8)	2.3 (±4.4)	4.9 (±12)	7.2 (±14.6)	9.1 (±16.2)
	Browsed	10.7	5.9	1.8	1.9	2.3	1.5
	Unbrowsed	20.3	8.6	6.2	22.2	22.3	23.5
Mean variation	Total	411.4	139.5	19.4	143.2	213.2	264.2

**Table 4:** Electivity values of browsed plants inventoried in transects located along the Gwda River, in three diameter classes for nine woody plant genera.

Shoot Diameter (cm)	*Alnus*	*Acer*	*Crataegus*	*Populus*	*Pinus*	*Prunus*	*Quercus*	*Salix*	*Sambucus*	Others
	Indice Value									
<2.5	0.01	0.44	−1.72	−2.59	nd	−0.40	nd	0.72	−0.67	−1.66
2.5–10	−0.94	0.10	−1.30	−1.43	−1.60	−0.63	−0.92	−0.04	−1.22	−1.77
>10	−2.96	−0.74	−135	−1.60	nd	nd	nd	nd	nd	−1.15

nd—no data due to no plants in the diameter class.

**Table 5:** The impact of plant diameter, plant genus and anthropopression level on observed differences in the selection of woody plants by beavers along the Gwda River, according to a generalized linear model with a binomial distribution.

Factor	Degrees of Freedom	Coefficients	Standard Error	Wald Statistics	*p-*Value
Intercept	1	0.3094	0.1095	4.0433	0.044
Diameter	1	0.0654	0.0074	38.8367	<0.001
Plant genus	9	3.6853	0.8257	307.8458	<0.001
Anthropopression Level	5	−1.7431	2.3534	35.3088	<0.001

## Supplementary Materials

The following is available online at https://www.mdpi.com/2076-2615/10/8/1376/s1. Table S1. Plants inventoried along the Gwda River on six transects, with information about shoot diameter, location (transect number), anthropopression level (in points), plant species, the genus name used in analysis, information if the plant was browsed and distance from the river bank. Table S2. Electivity index values for woody plants inventoried in each transect located along the Gwda River in three diameter classes for selected woody plants.
