# Supplementary material for: Eurasian Beaver (Castor fiber) Winter Foraging Preferences in Northern Poland—The Role of Woody Vegetation Composition and Anthropopression Level
Source: Animals (Basel). 2020 Aug 8;10(8):1376. doi: 10.3390/ani10081376 (PMC7460282; doi:10.3390/ani10081376)
Supplement: Supplementary file 1 [file animals-10-01376-s001.zip › Table S1.docx]

**Table S1**. Plants inventoried along the Gwda River on six transects, with information about shoot diameter, location (transect number), anthropopression level (in points), plant species, the genus name used in analysis, information if the plant was browsed and distance from the river bank.

| Shoot diameter [cm] | Transect number | Anthropopression level (in points) | Species | Genus | Browsed/Unbrowsed | Distance from the river bank class [m] |
| --- | --- | --- | --- | --- | --- | --- |
| 0,5 | 1 | 4 | *Sambucus nigra* | *Sambucus* | UNBROW | 21-30 |
| 0,5 | 1 | 4 | *Sambucus nigra* | *Sambucus* | UNBROW | 21-30 |
| 0,6 | 1 | 4 | *Sambucus nigra* | *Sambucus* | UNBROW | 21-30 |
| 0,4 | 1 | 4 | *Sambucus nigra* | *Sambucus* | UNBROW | 21-30 |
| 36,5 | 1 | 4 | *Alnus glutinosa* | *Alnus* | UNBROW | 21-30 |
| 8,1 | 1 | 4 | *Alnus glutinosa* | *Alnus* | UNBROW | 21-30 |
| 5,4 | 1 | 4 | *Sambucus nigra* | *Sambucus* | UNBROW | 21-30 |
| 4,4 | 1 | 4 | *Sambucus nigra* | *Sambucus* | UNBROW | 21-30 |
| 4,4 | 1 | 4 | *Sambucus nigra* | *Sambucus* | UNBROW | 21-30 |
| 3,4 | 1 | 4 | *Sambucus nigra* | *Sambucus* | UNBROW | 21-30 |
| 4,2 | 1 | 4 | *Alnus glutinosa* | *Alnus* | UNBROW | 11-20 |
| 5,7 | 1 | 4 | *Alnus glutinosa* | *Alnus* | UNBROW | 11-20 |
| 43,4 | 1 | 4 | *Alnus glutinosa* | *Alnus* | UNBROW | 11-20 |
| 5,5 | 1 | 4 | *Sambucus nigra* | *Sambucus* | UNBROW | 11-20 |
| 0,7 | 1 | 4 | *Sambucus nigra* | *Sambucus* | UNBROW | 11-20 |
| 1,9 | 1 | 4 | *Sambucus nigra* | *Sambucus* | UNBROW | 11-20 |
| 2,6 | 1 | 4 | *Sambucus nigra* | *Sambucus* | UNBROW | 11-20 |
| 1,4 | 1 | 4 | *Sambucus nigra* | *Sambucus* | UNBROW | 11-20 |
| 40,3 | 1 | 4 | *Alnus glutinosa* | *Alnus* | UNBROW | 11-20 |
| 23,2 | 1 | 4 | *Alnus glutinosa* | *Alnus* | UNBROW | 11-20 |
| 0,5 | 1 | 4 | *Sambucus nigra* | *Sambucus* | UNBROW | 0-10 |
| 56,1 | 1 | 4 | *Alnus glutinosa* | *Alnus* | UNBROW | 0-10 |
| 2,7 | 1 | 4 | *Alnus glutinosa* | *Alnus* | UNBROW | 0-10 |
| 48,5 | 1 | 4 | *Alnus glutinosa* | *Alnus* | UNBROW | 0-10 |
| 48,7 | 1 | 4 | *Alnus glutinosa* | *Alnus* | UNBROW | 0-10 |
| 34,2 | 1 | 4 | *Alnus glutinosa* | *Alnus* | UNBROW | 0-10 |
| 53 | 1 | 4 | *Alnus glutinosa* | *Alnus* | UNBROW | 0-10 |
| 6,2 | 1 | 4 | *Alnus glutinosa* | *Alnus* | UNBROW | 11-20 |
| 15,9 | 1 | 4 | *Alnus glutinosa* | *Alnus* | UNBROW | 11-20 |
| 0,7 | 1 | 4 | *Sambucus nigra* | *Sambucus* | UNBROW | 11-20 |
| 30,4 | 1 | 4 | *Alnus glutinosa* | *Alnus* | UNBROW | 21-30 |
| 4,9 | 1 | 4 | *Alnus glutinosa* | *Alnus* | UNBROW | 21-30 |
| 19,3 | 1 | 4 | *Alnus glutinosa* | *Alnus* | UNBROW | 21-30 |
| 1,9 | 1 | 4 | *Alnus glutinosa* | *Alnus* | UNBROW | 21-30 |
| 0,7 | 1 | 4 | *Sambucus nigra* | *Sambucus* | UNBROW | 21-30 |
| 24,5 | 1 | 4 | *Alnus glutinosa* | *Alnus* | UNBROW | 21-30 |
| 26,1 | 1 | 4 | *Alnus glutinosa* | *Alnus* | UNBROW | 21-30 |
| 23,7 | 1 | 4 | *Alnus glutinosa* | *Alnus* | UNBROW | 21-30 |
| 6,5 | 1 | 4 | *Alnus glutinosa* | *Alnus* | UNBROW | 21-30 |
| 56 | 1 | 4 | *Alnus glutinosa* | *Alnus* | UNBROW | 11-20 |
| 3,2 | 1 | 4 | *Sambucus nigra* | *Sambucus* | UNBROW | 11-20 |
| 4,4 | 1 | 4 | *Alnus glutinosa* | *Alnus* | UNBROW | 11-20 |
| 58 | 1 | 4 | *Alnus glutinosa* | *Alnus* | UNBROW | 0-10 |
| 36,7 | 1 | 4 | *Alnus glutinosa* | *Alnus* | UNBROW | 0-10 |
| 34,1 | 1 | 4 | *Alnus glutinosa* | *Alnus* | UNBROW | 0-10 |
| 6 | 1 | 4 | *Alnus glutinosa* | *Alnus* | BROW | 0-10 |
| 24,9 | 1 | 4 | *Alnus glutinosa* | *Alnus* | UNBROW | 0-10 |
| 23,5 | 1 | 4 | *Alnus glutinosa* | *Alnus* | UNBROW | 0-10 |
| 1,3 | 1 | 4 | *Alnus glutinosa* | *Alnus* | BROW | 0-10 |
| 0,6 | 1 | 4 | *Prunus spinosa* | *Prunus* | BROW | 0-10 |
| 1,1 | 1 | 4 | *Prunus spinosa* | *Prunus* | BROW | 0-10 |
| 1,1 | 1 | 4 | *Prunus spinosa* | *Prunus* | BROW | 0-10 |
| 0,6 | 1 | 4 | *Prunus spinosa* | *Prunus* | BROW | 0-10 |
| 1,9 | 1 | 4 | *Prunus spinosa* | *Prunus* | BROW | 0-10 |
| 7,5 | 1 | 4 | *Prunus spinosa* | *Prunus* | BROW | 0-10 |
| 70 | 1 | 4 | *Alnus glutinosa* | *Alnus* | UNBROW | 0-10 |
| 8,2 | 1 | 4 | *Alnus glutinosa* | *Alnus* | UNBROW | 0-10 |
| 3,3 | 1 | 4 | *Alnus glutinosa* | *Alnus* | UNBROW | 0-10 |
| 14,1 | 1 | 4 | *Alnus glutinosa* | *Alnus* | UNBROW | 0-10 |
| 4,4 | 1 | 4 | *Alnus glutinosa* | *Alnus* | UNBROW | 0-10 |
| 4,3 | 1 | 4 | *Alnus glutinosa* | *Alnus* | UNBROW | 0-10 |
| 5,2 | 1 | 4 | *Alnus glutinosa* | *Alnus* | UNBROW | 0-10 |
| 12,5 | 1 | 4 | *Alnus glutinosa* | *Alnus* | UNBROW | 0-10 |
| 6,3 | 1 | 4 | *Alnus glutinosa* | *Alnus* | UNBROW | 0-10 |
| 7,2 | 1 | 4 | *Alnus glutinosa* | *Alnus* | UNBROW | 0-10 |
| 6,8 | 1 | 4 | *Alnus glutinosa* | *Alnus* | UNBROW | 0-10 |
| 0,7 | 1 | 4 | *Alnus glutinosa* | *Alnus* | UNBROW | 0-10 |
| 2 | 1 | 4 | *Alnus glutinosa* | *Alnus* | BROW | 0-10 |
| 2,1 | 1 | 4 | *Alnus glutinosa* | *Alnus* | BROW | 0-10 |
| 2,5 | 1 | 4 | *Alnus glutinosa* | *Alnus* | BROW | 0-10 |
| 40 | 1 | 4 | *Alnus glutinosa* | *Alnus* | UNBROW | 21-30 |
| 1,3 | 1 | 4 | *Alnus glutinosa* | *Alnus* | UNBROW | 21-30 |
| 1,2 | 1 | 4 | *Sambucus nigra* | *Sambucus* | UNBROW | 21-30 |
| 39,7 | 1 | 4 | *Sambucus nigra* | *Sambucus* | UNBROW | 21-30 |
| 2,5 | 1 | 4 | *Alnus glutinosa* | *Alnus* | UNBROW | 0-10 |
| 1,5 | 1 | 4 | *Sambucus nigra* | *Sambucus* | UNBROW | 0-10 |
| 4,1 | 1 | 4 | *Sambucus nigra* | *Sambucus* | BROW | 0-10 |
| 6,7 | 1 | 4 | *Sambucus nigra* | *Sambucus* | BROW | 0-10 |
| 42,6 | 1 | 4 | *Sambucus nigra* | *Sambucus* | UNBROW | 0-10 |
| 42,8 | 1 | 4 | *Alnus glutinosa* | *Alnus* | UNBROW | 0-10 |
| 35,9 | 1 | 4 | *Alnus glutinosa* | *Alnus* | UNBROW | 0-10 |
| 39,5 | 1 | 4 | *Alnus glutinosa* | *Alnus* | UNBROW | 0-10 |
| 7,2 | 1 | 4 | *Alnus glutinosa* | *Alnus* | BROW | 0-10 |
| 47,9 | 1 | 4 | *Alnus glutinosa* | *Alnus* | UNBROW | 0-10 |
| 58,2 | 1 | 4 | *Alnus glutinosa* | *Alnus* | UNBROW | 0-10 |
| 45,4 | 1 | 4 | *Alnus glutinosa* | *Alnus* | UNBROW | 0-10 |
| 1 | 1 | 4 | *Alnus glutinosa* | *Alnus* | UNBROW | 0-10 |
| 1,1 | 1 | 4 | *Sambucus nigra* | *Sambucus* | UNBROW | 0-10 |
| 1,1 | 1 | 4 | *Sambucus nigra* | *Sambucus* | UNBROW | 0-10 |
| 1,5 | 1 | 4 | *Alnus glutinosa* | *Alnus* | UNBROW | 0-10 |
| 41 | 1 | 4 | *Alnus glutinosa* | *Alnus* | UNBROW | 11-20 |
| 3,1 | 1 | 4 | *Alnus glutinosa* | *Alnus* | BROW | 11-20 |
| 40,1 | 1 | 4 | *Alnus glutinosa* | *Alnus* | UNBROW | 11-20 |
| 48 | 1 | 4 | *Alnus glutinosa* | *Alnus* | UNBROW | 11-20 |
| 0,5 | 1 | 4 | *Prunus spinosa* | *Prunus* | UNBROW | 11-20 |
| 8,2 | 1 | 4 | *Alnus glutinosa* | *Alnus* | UNBROW | 11-20 |
| 35,5 | 1 | 4 | *Alnus glutinosa* | *Alnus* | UNBROW | 11-20 |
| 40 | 1 | 4 | *Alnus glutinosa* | *Alnus* | UNBROW | 11-20 |
| 34,6 | 1 | 4 | *Alnus glutinosa* | *Alnus* | UNBROW | 11-20 |
| 7,8 | 1 | 4 | *Alnus glutinosa* | *Alnus* | BROW | 11-20 |
| 8,3 | 1 | 4 | *Alnus glutinosa* | *Alnus* | UNBROW | 11-20 |
| 8,6 | 1 | 4 | *Crataegus* sp. | *Crataegus* | BROW | 11-20 |
| 20,5 | 1 | 4 | *Crataegus* sp. | *Crataegus* | UNBROW | 11-20 |
| 54,6 | 1 | 4 | *Crataegus* sp. | *Crataegus* | UNBROW | 11-20 |
| 6 | 1 | 4 | *Crataegus* sp. | *Crataegus* | UNBROW | 11-20 |
| 7,5 | 1 | 4 | *Crataegus* sp. | *Crataegus* | UNBROW | 11-20 |
| 48,1 | 1 | 4 | *Crataegus* sp. | *Crataegus* | UNBROW | 11-20 |
| 14,4 | 1 | 4 | *Crataegus* sp. | *Crataegus* | UNBROW | 11-20 |
| 80 | 1 | 4 | *Populus nigra* | *Populus* | BROW | 11-20 |
| 3,3 | 1 | 4 | *Crataegus* sp. | *Crataegus* | UNBROW | 11-20 |
| 5 | 1 | 4 | *Crataegus* sp. | *Crataegus* | UNBROW | 11-20 |
| 13,5 | 1 | 4 | *Crataegus* sp. | *Crataegus* | UNBROW | 11-20 |
| 11 | 1 | 4 | *Crataegus* sp. | *Crataegus* | UNBROW | 11-20 |
| 9,3 | 1 | 4 | *Crataegus* sp. | *Crataegus* | UNBROW | 11-20 |
| 24,1 | 1 | 4 | *Tilia cordata* | Others | BROW | 11-20 |
| 32,6 | 1 | 4 | *Alnus glutinosa* | *Alnus* | UNBROW | 0-10 |
| 39,5 | 1 | 4 | *Alnus glutinosa* | *Alnus* | UNBROW | 0-10 |
| 44,5 | 1 | 4 | *Alnus glutinosa* | *Alnus* | UNBROW | 0-10 |
| 22,6 | 1 | 4 | *Alnus glutinosa* | *Alnus* | UNBROW | 0-10 |
| 80 | 1 | 4 | *Populus nigra* | *Populus* | UNBROW | 0-10 |
| 29,6 | 1 | 4 | *Alnus glutinosa* | *Alnus* | UNBROW | 0-10 |
| 16,9 | 1 | 4 | *Crataegus* sp. | *Crataegus* | UNBROW | 11-20 |
| 42,3 | 1 | 4 | *Alnus glutinosa* | *Alnus* | UNBROW | 11-20 |
| 10,5 | 1 | 4 | *Alnus glutinosa* | *Alnus* | UNBROW | 11-20 |
| 22 | 1 | 4 | *Alnus glutinosa* | *Alnus* | UNBROW | 11-20 |
| 24 | 1 | 4 | *Alnus glutinosa* | *Alnus* | UNBROW | 11-20 |
| 5,4 | 1 | 4 | *Sambucus nigra* | *Sambucus* | UNBROW | 11-20 |
| 25,5 | 1 | 4 | *Alnus glutinosa* | *Alnus* | UNBROW | 11-20 |
| 45 | 1 | 4 | *Alnus glutinosa* | *Alnus* | UNBROW | 11-20 |
| 5,9 | 1 | 4 | *Sambucus nigra* | *Sambucus* | UNBROW | 11-20 |
| 7 | 1 | 4 | *Sambucus nigra* | *Sambucus* | UNBROW | 11-20 |
| 80 | 1 | 4 | *Populus nigra* | *Populus* | UNBROW | 11-20 |
| 80 | 1 | 4 | *Populus nigra* | *Populus* | UNBROW | 11-20 |
| 47,7 | 1 | 4 | *Alnus glutinosa* | *Alnus* | UNBROW | 11-20 |
| 28,9 | 1 | 4 | *Alnus glutinosa* | *Alnus* | UNBROW | 11-20 |
| 3 | 1 | 4 | *Sambucus nigra* | *Sambucus* | UNBROW | 11-20 |
| 3 | 1 | 4 | *Sambucus nigra* | *Sambucus* | UNBROW | 11-20 |
| 5 | 1 | 4 | *Sambucus nigra* | *Sambucus* | UNBROW | 11-20 |
| 6 | 1 | 4 | *Sambucus nigra* | *Sambucus* | UNBROW | 11-20 |
| 7 | 1 | 4 | *Sambucus nigra* | *Sambucus* | UNBROW | 11-20 |
| 49 | 1 | 4 | *Tilia cordata* | Others | BROW | 11-20 |
| 32,1 | 1 | 4 | *Quercus robur* | *Quercus* | UNBROW | 11-20 |
| 16,3 | 1 | 4 | *Crataegus* sp. | *Crataegus* | UNBROW | 11-20 |
| 13,5 | 1 | 4 | *Crataegus* sp. | *Crataegus* | BROW | 11-20 |
| 5 | 1 | 4 | *Crataegus* sp. | *Crataegus* | BROW | 11-20 |
| 4 | 1 | 4 | *Crataegus* sp. | *Crataegus* | UNBROW | 11-20 |
| 6 | 1 | 4 | *Crataegus* sp. | *Crataegus* | UNBROW | 11-20 |
| 6,1 | 1 | 4 | *Crataegus* sp. | *Crataegus* | UNBROW | 11-20 |
| 4,4 | 1 | 4 | *Sambucus nigra* | *Sambucus* | UNBROW | 11-20 |
| 9,4 | 1 | 4 | *Crataegus* sp. | *Crataegus* | UNBROW | 11-20 |
| 20,9 | 1 | 4 | *Alnus glutinosa* | *Alnus* | UNBROW | 0-10 |
| 26,8 | 2 | 5 | *Alnus glutinosa* | *Alnus* | UNBROW | 0-10 |
| 8,1 | 2 | 5 | *Alnus glutinosa* | *Alnus* | BROW | 0-10 |
| 7,3 | 2 | 5 | *Prunus spinosa* | *Prunus* | BROW | 0-10 |
| 4,5 | 2 | 5 | *Prunus spinosa* | *Prunus* | BROW | 0-10 |
| 3,3 | 2 | 5 | *Prunus spinosa* | *Prunus* | BROW | 0-10 |
| 6,4 | 2 | 5 | *Prunus spinosa* | *Prunus* | BROW | 0-10 |
| 4,5 | 2 | 5 | *Prunus spinosa* | *Prunus* | BROW | 0-10 |
| 5,4 | 2 | 5 | *Prunus spinosa* | *Prunus* | BROW | 0-10 |
| 2,9 | 2 | 5 | *Prunus spinosa* | *Prunus* | BROW | 0-10 |
| 2,7 | 2 | 5 | *Prunus spinosa* | *Prunus* | BROW | 0-10 |
| 5,9 | 2 | 5 | *Prunus spinosa* | *Prunus* | BROW | 0-10 |
| 7,9 | 2 | 5 | *Prunus spinosa* | *Prunus* | BROW | 0-10 |
| 4,4 | 2 | 5 | *Prunus spinosa* | *Prunus* | BROW | 0-10 |
| 0,7 | 2 | 5 | *Prunus spinosa* | *Prunus* | BROW | 0-10 |
| 1,4 | 2 | 5 | *Prunus spinosa* | *Prunus* | BROW | 0-10 |
| 2,6 | 2 | 5 | *Prunus spinosa* | *Prunus* | BROW | 0-10 |
| 2,2 | 2 | 5 | *Prunus spinosa* | *Prunus* | BROW | 0-10 |
| 5,7 | 2 | 5 | *Prunus spinosa* | *Prunus* | UNBROW | 0-10 |
| 0,6 | 2 | 5 | *Prunus spinosa* | *Prunus* | UNBROW | 0-10 |
| 1,3 | 2 | 5 | *Prunus spinosa* | *Prunus* | UNBROW | 0-10 |
| 2,2 | 2 | 5 | *Prunus spinosa* | *Prunus* | UNBROW | 0-10 |
| 5,6 | 2 | 5 | *Prunus spinosa* | *Prunus* | UNBROW | 0-10 |
| 3,6 | 2 | 5 | *Prunus spinosa* | *Prunus* | UNBROW | 0-10 |
| 3,7 | 2 | 5 | *Prunus spinosa* | *Prunus* | UNBROW | 0-10 |
| 1,6 | 2 | 5 | *Prunus spinosa* | *Prunus* | UNBROW | 0-10 |
| 5,4 | 2 | 5 | *Prunus spinosa* | *Prunus* | BROW | 0-10 |
| 5,9 | 2 | 5 | *Prunus spinosa* | *Prunus* | BROW | 0-10 |
| 4,3 | 2 | 5 | *Prunus spinosa* | *Prunus* | BROW | 0-10 |
| 3,5 | 2 | 5 | *Prunus spinosa* | *Prunus* | BROW | 0-10 |
| 4,5 | 2 | 5 | *Prunus spinosa* | *Prunus* | BROW | 0-10 |
| 4,1 | 2 | 5 | *Prunus spinosa* | *Prunus* | BROW | 0-10 |
| 4,8 | 2 | 5 | *Prunus spinosa* | *Prunus* | BROW | 0-10 |
| 2,4 | 2 | 5 | *Prunus spinosa* | *Prunus* | BROW | 0-10 |
| 20,4 | 2 | 5 | *Populus nigra* | *Populus* | BROW | 0-10 |
| 3,3 | 2 | 5 | *Prunus spinosa* | *Prunus* | BROW | 0-10 |
| 1,2 | 2 | 5 | *Prunus spinosa* | *Prunus* | BROW | 0-10 |
| 3,7 | 2 | 5 | *Alnus glutinosa* | *Alnus* | BROW | 0-10 |
| 3,7 | 2 | 5 | *Alnus glutinosa* | *Alnus* | BROW | 0-10 |
| 3,7 | 2 | 5 | *Alnus glutinosa* | *Alnus* | BROW | 0-10 |
| 2,6 | 2 | 5 | *Alnus glutinosa* | *Alnus* | BROW | 0-10 |
| 4,3 | 2 | 5 | *Alnus glutinosa* | *Alnus* | BROW | 0-10 |
| 3,5 | 2 | 5 | *Alnus glutinosa* | *Alnus* | BROW | 0-10 |
| 4 | 2 | 5 | *Alnus glutinosa* | *Alnus* | BROW | 0-10 |
| 18,7 | 2 | 5 | *Alnus glutinosa* | *Alnus* | BROW | 0-10 |
| 23 | 2 | 5 | *Alnus glutinosa* | *Alnus* | UNBROW | 0-10 |
| 1,7 | 2 | 5 | *Prunus spinosa* | *Prunus* | BROW | 0-10 |
| 0,5 | 2 | 5 | *Prunus spinosa* | *Prunus* | BROW | 0-10 |
| 1,5 | 2 | 5 | *Alnus glutinosa* | *Alnus* | BROW | 0-10 |
| 0,5 | 2 | 5 | *Alnus glutinosa* | *Alnus* | BROW | 0-10 |
| 4,6 | 2 | 5 | *Alnus glutinosa* | *Alnus* | UNBROW | 0-10 |
| 31,1 | 2 | 5 | *Alnus glutinosa* | *Alnus* | UNBROW | 0-10 |
| 34,1 | 2 | 5 | *Alnus glutinosa* | *Alnus* | UNBROW | 0-10 |
| 1,7 | 2 | 5 | *Prunus spinosa* | *Prunus* | BROW | 0-10 |
| 1,2 | 2 | 5 | *Prunus spinosa* | *Prunus* | BROW | 0-10 |
| 1,5 | 2 | 5 | *Prunus spinosa* | *Prunus* | UNBROW | 0-10 |
| 0,3 | 2 | 5 | *Populus nigra* | *Populus* | UNBROW | 0-10 |
| 1 | 2 | 5 | *Populus nigra* | *Populus* | BROW | 0-10 |
| 0,5 | 2 | 5 | *Populus nigra* | *Populus* | UNBROW | 0-10 |
| 2,5 | 2 | 5 | *Populus nigra* | *Populus* | BROW | 0-10 |
| 0,6 | 2 | 5 | *Prunus spinosa* | *Prunus* | UNBROW | 0-10 |
| 0,3 | 2 | 5 | *Prunus spinosa* | *Prunus* | UNBROW | 0-10 |
| 0,4 | 2 | 5 | *Prunus spinosa* | *Prunus* | UNBROW | 0-10 |
| 1 | 2 | 5 | *Prunus spinosa* | *Prunus* | UNBROW | 0-10 |
| 2,3 | 2 | 5 | *Prunus spinosa* | *Prunus* | UNBROW | 0-10 |
| 80 | 2 | 5 | *Populus nigra* | *Populus* | BROW | 0-10 |
| 7,2 | 2 | 5 | *Populus nigra* | *Populus* | UNBROW | 0-10 |
| 52 | 2 | 5 | *Alnus glutinosa* | *Alnus* | UNBROW | 0-10 |
| 11 | 2 | 5 | *Alnus glutinosa* | *Alnus* | UNBROW | 0-10 |
| 20,7 | 2 | 5 | *Alnus glutinosa* | *Alnus* | BROW | 0-10 |
| 1,1 | 2 | 5 | *Alnus glutinosa* | *Alnus* | BROW | 0-10 |
| 39 | 2 | 5 | *Alnus glutinosa* | *Alnus* | UNBROW | 0-10 |
| 37,7 | 2 | 5 | *Populus nigra* | *Populus* | UNBROW | 0-10 |
| 29,4 | 2 | 5 | *Populus nigra* | *Populus* | UNBROW | 0-10 |
| 33,5 | 2 | 5 | *Alnus glutinosa* | *Alnus* | UNBROW | 0-10 |
| 0,5 | 2 | 5 | *Alnus glutinosa* | *Alnus* | BROW | 0-10 |
| 0,5 | 2 | 5 | *Alnus glutinosa* | *Alnus* | BROW | 0-10 |
| 0,9 | 2 | 5 | *Alnus glutinosa* | *Alnus* | BROW | 0-10 |
| 1,4 | 2 | 5 | *Alnus glutinosa* | *Alnus* | BROW | 0-10 |
| 1,9 | 2 | 5 | *Alnus glutinosa* | *Alnus* | BROW | 0-10 |
| 1,1 | 2 | 5 | *Alnus glutinosa* | *Alnus* | BROW | 0-10 |
| 1 | 2 | 5 | *Alnus glutinosa* | *Alnus* | BROW | 0-10 |
| 0,4 | 2 | 5 | *Alnus glutinosa* | *Alnus* | BROW | 0-10 |
| 3 | 2 | 5 | *Alnus glutinosa* | *Alnus* | BROW | 0-10 |
| 2,9 | 2 | 5 | *Alnus glutinosa* | *Alnus* | BROW | 0-10 |
| 3,1 | 2 | 5 | *Alnus glutinosa* | *Alnus* | BROW | 0-10 |
| 3 | 2 | 5 | *Prunus spinosa* | *Prunus* | UNBROW | 0-10 |
| 3 | 2 | 5 | *Prunus spinosa* | *Prunus* | UNBROW | 0-10 |
| 2 | 2 | 5 | *Prunus spinosa* | *Prunus* | UNBROW | 0-10 |
| 3,2 | 2 | 5 | *Prunus spinosa* | *Prunus* | BROW | 0-10 |
| 4 | 2 | 5 | *Pinus sylvestris* | *Pinus* | BROW | 11-20 |
| 7,5 | 2 | 5 | *Pinus sylvestris* | *Pinus* | UNBROW | 11-20 |
| 1,8 | 2 | 5 | *Populus nigra* | *Populus* | UNBROW | 11-20 |
| 2 | 2 | 5 | *Quercus rubra* | *Quercus* | UNBROW | 11-20 |
| 2,3 | 2 | 5 | *Quercus rubra* | *Quercus* | UNBROW | 11-20 |
| 13,3 | 2 | 5 | *Pinus sylvestris* | *Pinus* | UNBROW | 11-20 |
| 1,5 | 2 | 5 | *Populus nigra* | *Populus* | UNBROW | 11-20 |
| 1,3 | 2 | 5 | *Populus nigra* | *Populus* | UNBROW | 11-20 |
| 1,2 | 2 | 5 | *Populus nigra* | *Populus* | UNBROW | 11-20 |
| 2 | 2 | 5 | *Prunus spinosa* | *Prunus* | UNBROW | 11-20 |
| 1 | 2 | 5 | *Pinus sylvestris* | *Pinus* | UNBROW | 11-20 |
| 0,6 | 2 | 5 | *Pinus sylvestris* | *Pinus* | UNBROW | 11-20 |
| 2,5 | 2 | 5 | *Pinus sylvestris* | *Pinus* | UNBROW | 11-20 |
| 1,6 | 2 | 5 | *Populus nigra* | *Populus* | UNBROW | 11-20 |
| 1 | 2 | 5 | *Populus nigra* | *Populus* | UNBROW | 11-20 |
| 2,4 | 2 | 5 | *Pinus sylvestris* | *Pinus* | UNBROW | 11-20 |
| 7,7 | 2 | 5 | *Populus nigra* | *Populus* | UNBROW | 11-20 |
| 27,4 | 2 | 5 | *Pinus sylvestris* | *Pinus* | UNBROW | 21-30 |
| 19,1 | 2 | 5 | *Pinus sylvestris* | *Pinus* | UNBROW | 21-30 |
| 11 | 2 | 5 | *Pinus sylvestris* | *Pinus* | UNBROW | 21-30 |
| 7,4 | 2 | 5 | *Pinus sylvestris* | *Pinus* | UNBROW | 21-30 |
| 3,9 | 2 | 5 | *Quercus rubra* | *Quercus* | UNBROW | 21-30 |
| 2,4 | 2 | 5 | *Quercus rubra* | *Quercus* | UNBROW | 21-30 |
| 25,5 | 2 | 5 | *Pinus sylvestris* | *Pinus* | UNBROW | 21-30 |
| 22,2 | 2 | 5 | *Pinus sylvestris* | *Pinus* | UNBROW | 21-30 |
| 9,4 | 2 | 5 | *Pinus sylvestris* | *Pinus* | UNBROW | 21-30 |
| 3,7 | 2 | 5 | *Pinus sylvestris* | *Pinus* | BROW | 21-30 |
| 4,7 | 2 | 5 | *Pinus sylvestris* | *Pinus* | BROW | 21-30 |
| 17,9 | 2 | 5 | *Betula pubescens* | Others | UNBROW | 21-30 |
| 20,5 | 2 | 5 | *Pinus sylvestris* | *Pinus* | UNBROW | 21-30 |
| 3,6 | 2 | 5 | *Pinus sylvestris* | *Pinus* | UNBROW | 21-30 |
| 4,6 | 2 | 5 | *Pinus sylvestris* | *Pinus* | UNBROW | 21-30 |
| 3,8 | 2 | 5 | *Pinus sylvestris* | *Pinus* | BROW | 21-30 |
| 8 | 2 | 5 | *Pinus sylvestris* | *Pinus* | UNBROW | 21-30 |
| 5,6 | 2 | 5 | *Pinus sylvestris* | *Pinus* | UNBROW | 21-30 |
| 20 | 2 | 5 | *Pinus sylvestris* | *Pinus* | UNBROW | 21-30 |
| 13 | 2 | 5 | *Pinus sylvestris* | *Pinus* | UNBROW | 21-30 |
| 9,4 | 2 | 5 | *Betula pubescens* | Others | UNBROW | 21-30 |
| 7,9 | 2 | 5 | *Pinus sylvestris* | *Pinus* | UNBROW | 21-30 |
| 23,2 | 2 | 5 | *Pinus sylvestris* | *Pinus* | UNBROW | 21-30 |
| 11,7 | 2 | 5 | *Pinus sylvestris* | *Pinus* | UNBROW | 21-30 |
| 15 | 2 | 5 | *Pinus sylvestris* | *Pinus* | UNBROW | 21-30 |
| 3 | 2 | 5 | *Alnus glutinosa* | *Alnus* | UNBROW | 21-30 |
| 2 | 2 | 5 | *Alnus glutinosa* | *Alnus* | UNBROW | 21-30 |
| 2,1 | 2 | 5 | *Alnus glutinosa* | *Alnus* | BROW | 21-30 |
| 80 | 2 | 5 | *Populus nigra* | *Populus* | BROW | 0-10 |
| 2 | 2 | 5 | *Alnus glutinosa* | *Alnus* | BROW | 0-10 |
| 3 | 2 | 5 | *Prunus spinosa* | *Prunus* | BROW | 0-10 |
| 2 | 2 | 5 | *Populus nigra* | *Populus* | UNBROW | 0-10 |
| 1,6 | 2 | 5 | *Populus nigra* | *Populus* | UNBROW | 0-10 |
| 27,7 | 2 | 5 | *Alnus glutinosa* | *Alnus* | UNBROW | 0-10 |
| 11 | 2 | 5 | *Alnus glutinosa* | *Alnus* | BROW | 0-10 |
| 3,3 | 2 | 5 | *Populus nigra* | *Populus* | UNBROW | 0-10 |
| 2 | 2 | 5 | *Populus nigra* | *Populus* | UNBROW | 0-10 |
| 2,1 | 2 | 5 | *Populus nigra* | *Populus* | UNBROW | 0-10 |
| 2,6 | 2 | 5 | *Populus nigra* | *Populus* | UNBROW | 0-10 |
| 1,5 | 2 | 5 | *Populus nigra* | *Populus* | UNBROW | 0-10 |
| 1,5 | 2 | 5 | *Populus nigra* | *Populus* | UNBROW | 0-10 |
| 1,6 | 2 | 5 | *Populus nigra* | *Populus* | UNBROW | 0-10 |
| 1,1 | 2 | 5 | *Populus nigra* | *Populus* | UNBROW | 0-10 |
| 1,1 | 2 | 5 | *Populus nigra* | *Populus* | UNBROW | 0-10 |
| 2,4 | 2 | 5 | *Populus nigra* | *Populus* | UNBROW | 0-10 |
| 1,1 | 2 | 5 | *Corylus avellana* | Others | UNBROW | 0-10 |
| 0,6 | 2 | 5 | *Corylus avellana* | Others | BROW | 0-10 |
| 1,7 | 2 | 5 | *Corylus avellana* | Others | UNBROW | 0-10 |
| 1,6 | 2 | 5 | *Corylus avellana* | Others | UNBROW | 0-10 |
| 1,5 | 2 | 5 | *Corylus avellana* | Others | UNBROW | 0-10 |
| 1,6 | 2 | 5 | *Corylus avellana* | Others | UNBROW | 0-10 |
| 59,6 | 2 | 5 | *Alnus glutinosa* | *Alnus* | UNBROW | 0-10 |
| 61,4 | 2 | 5 | *Alnus glutinosa* | *Alnus* | UNBROW | 0-10 |
| 5,5 | 2 | 5 | *Prunus spinosa* | *Prunus* | UNBROW | 0-10 |
| 5,3 | 2 | 5 | *Prunus spinosa* | *Prunus* | UNBROW | 0-10 |
| 1,5 | 2 | 5 | *Carpinus betulus* | Others | UNBROW | 0-10 |
| 1,6 | 2 | 5 | *Carpinus betulus* | Others | UNBROW | 0-10 |
| 1 | 2 | 5 | *Carpinus betulus* | Others | UNBROW | 0-10 |
| 1 | 2 | 5 | *Carpinus betulus* | Others | UNBROW | 0-10 |
| 1,6 | 2 | 5 | *Carpinus betulus* | Others | BROW | 0-10 |
| 2 | 2 | 5 | *Prunus spinosa* | *Prunus* | UNBROW | 0-10 |
| 2,7 | 2 | 5 | *Crataegus* sp. | *Crataegus* | BROW | 0-10 |
| 1,5 | 2 | 5 | *Crataegus* sp. | *Crataegus* | UNBROW | 0-10 |
| 80 | 2 | 5 | *Populus nigra* | *Populus* | UNBROW | 0-10 |
| 2 | 2 | 5 | *Acer platanoides* | *Acer* | BROW | 0-10 |
| 1,2 | 2 | 5 | *Acer platanoides* | *Acer* | BROW | 0-10 |
| 5 | 2 | 5 | *Acer platanoides* | *Acer* | BROW | 0-10 |
| 2,3 | 2 | 5 | *Crataegus* sp. | *Crataegus* | BROW | 0-10 |
| 6,9 | 2 | 5 | *Pinus sylvestris* | *Pinus* | BROW | 11-20 |
| 1,3 | 2 | 5 | *Crataegus* sp. | *Crataegus* | UNBROW | 11-20 |
| 22,6 | 2 | 5 | *Pinus sylvestris* | *Pinus* | UNBROW | 11-20 |
| 15 | 2 | 5 | *Pinus sylvestris* | *Pinus* | UNBROW | 11-20 |
| 13,9 | 2 | 5 | *Pinus sylvestris* | *Pinus* | UNBROW | 11-20 |
| 24,1 | 2 | 5 | *Pinus sylvestris* | *Pinus* | UNBROW | 21-30 |
| 11,4 | 2 | 5 | *Alnus glutinosa* | *Alnus* | UNBROW | 21-30 |
| 8,9 | 2 | 5 | *Alnus glutinosa* | *Alnus* | UNBROW | 21-30 |
| 4,3 | 2 | 5 | *Alnus glutinosa* | *Alnus* | UNBROW | 21-30 |
| 2 | 2 | 5 | *Alnus glutinosa* | *Alnus* | UNBROW | 21-30 |
| 4,4 | 2 | 5 | *Alnus glutinosa* | *Alnus* | UNBROW | 21-30 |
| 11,1 | 2 | 5 | *Populus nigra* | *Populus* | UNBROW | 11-20 |
| 16,7 | 2 | 5 | *Salix alba* | *Salix* | UNBROW | 21-30 |
| 9,5 | 2 | 5 | *Alnus glutinosa* | *Alnus* | UNBROW | 21-30 |
| 9,8 | 2 | 5 | *Alnus glutinosa* | *Alnus* | UNBROW | 21-30 |
| 9,4 | 2 | 5 | *Betula pubescens* | Others | UNBROW | 21-30 |
| 7,3 | 2 | 5 | *Betula pubescens* | Others | UNBROW | 21-30 |
| 15,7 | 2 | 5 | *Pinus sylvestris* | *Pinus* | UNBROW | 21-30 |
| 14,5 | 2 | 5 | *Alnus glutinosa* | *Alnus* | UNBROW | 21-30 |
| 14,6 | 2 | 5 | *Pinus sylvestris* | *Pinus* | UNBROW | 11-20 |
| 20,8 | 2 | 5 | *Pinus sylvestris* | *Pinus* | UNBROW | 11-20 |
| 25,3 | 2 | 5 | *Pinus sylvestris* | *Pinus* | UNBROW | 11-20 |
| 6 | 2 | 5 | *Crataegus* sp. | *Crataegus* | UNBROW | 21-30 |
| 17 | 2 | 5 | *Pinus sylvestris* | *Pinus* | UNBROW | 21-30 |
| 17,1 | 2 | 5 | *Pinus sylvestris* | *Pinus* | UNBROW | 21-30 |
| 16,7 | 2 | 5 | *Pinus sylvestris* | *Pinus* | UNBROW | 21-30 |
| 15,4 | 2 | 5 | *Pinus sylvestris* | *Pinus* | UNBROW | 21-30 |
| 16,6 | 2 | 5 | *Pinus sylvestris* | *Pinus* | UNBROW | 21-30 |
| 10 | 2 | 5 | *Pinus sylvestris* | *Pinus* | UNBROW | 21-30 |
| 11,4 | 2 | 5 | *Pinus sylvestris* | *Pinus* | UNBROW | 21-30 |
| 12,2 | 2 | 5 | *Pinus sylvestris* | *Pinus* | UNBROW | 21-30 |
| 3,1 | 2 | 5 | *Alnus glutinosa* | *Alnus* | UNBROW | 21-30 |
| 4,1 | 2 | 5 | *Alnus glutinosa* | *Alnus* | UNBROW | 21-30 |
| 3,9 | 2 | 5 | *Alnus glutinosa* | *Alnus* | UNBROW | 21-30 |
| 2,4 | 2 | 5 | *Alnus glutinosa* | *Alnus* | UNBROW | 21-30 |
| 2,5 | 2 | 5 | *Alnus glutinosa* | *Alnus* | UNBROW | 21-30 |
| 2,6 | 2 | 5 | *Alnus glutinosa* | *Alnus* | UNBROW | 21-30 |
| 2,5 | 2 | 5 | *Alnus glutinosa* | *Alnus* | UNBROW | 21-30 |
| 2,5 | 2 | 5 | *Alnus glutinosa* | *Alnus* | UNBROW | 21-30 |
| 2,9 | 2 | 5 | *Alnus glutinosa* | *Alnus* | UNBROW | 21-30 |
| 3,1 | 2 | 5 | *Alnus glutinosa* | *Alnus* | UNBROW | 21-30 |
| 3,3 | 2 | 5 | *Alnus glutinosa* | *Alnus* | UNBROW | 21-30 |
| 8,9 | 2 | 5 | *Alnus glutinosa* | *Alnus* | UNBROW | 21-30 |
| 9,7 | 2 | 5 | *Alnus glutinosa* | *Alnus* | UNBROW | 21-30 |
| 14,4 | 2 | 5 | *Alnus glutinosa* | *Alnus* | UNBROW | 21-30 |
| 10 | 2 | 5 | *Pinus sylvestris* | *Pinus* | UNBROW | 21-30 |
| 10 | 2 | 5 | *Pinus sylvestris* | *Pinus* | UNBROW | 21-30 |
| 10 | 2 | 5 | *Pinus sylvestris* | *Pinus* | UNBROW | 21-30 |
| 10 | 2 | 5 | *Pinus sylvestris* | *Pinus* | UNBROW | 21-30 |
| 10 | 2 | 5 | *Pinus sylvestris* | *Pinus* | UNBROW | 21-30 |
| 10 | 2 | 5 | *Pinus sylvestris* | *Pinus* | UNBROW | 21-30 |
| 10 | 2 | 5 | *Pinus sylvestris* | *Pinus* | UNBROW | 21-30 |
| 10 | 2 | 5 | *Pinus sylvestris* | *Pinus* | UNBROW | 21-30 |
| 10 | 2 | 5 | *Pinus sylvestris* | *Pinus* | UNBROW | 21-30 |
| 10 | 2 | 5 | *Pinus sylvestris* | *Pinus* | UNBROW | 21-30 |
| 10 | 2 | 5 | *Pinus sylvestris* | *Pinus* | UNBROW | 21-30 |
| 10 | 2 | 5 | *Pinus sylvestris* | *Pinus* | UNBROW | 21-30 |
| 10 | 2 | 5 | *Pinus sylvestris* | *Pinus* | UNBROW | 21-30 |
| 10 | 2 | 5 | *Pinus sylvestris* | *Pinus* | UNBROW | 21-30 |
| 10 | 2 | 5 | *Pinus sylvestris* | *Pinus* | UNBROW | 21-30 |
| 10 | 2 | 5 | *Pinus sylvestris* | *Pinus* | UNBROW | 21-30 |
| 10 | 2 | 5 | *Pinus sylvestris* | *Pinus* | UNBROW | 21-30 |
| 10 | 2 | 5 | *Pinus sylvestris* | *Pinus* | UNBROW | 21-30 |
| 10 | 2 | 5 | *Pinus sylvestris* | *Pinus* | UNBROW | 21-30 |
| 10 | 2 | 5 | *Pinus sylvestris* | *Pinus* | UNBROW | 21-30 |
| 3,5 | 2 | 5 | *Prunus spinosa* | *Prunus* | UNBROW | 11-20 |
| 3,5 | 2 | 5 | *Prunus spinosa* | *Prunus* | UNBROW | 11-20 |
| 3,5 | 2 | 5 | *Prunus spinosa* | *Prunus* | UNBROW | 11-20 |
| 3,5 | 2 | 5 | *Prunus spinosa* | *Prunus* | UNBROW | 11-20 |
| 3,5 | 2 | 5 | *Prunus spinosa* | *Prunus* | UNBROW | 11-20 |
| 3,5 | 2 | 5 | *Prunus spinosa* | *Prunus* | UNBROW | 11-20 |
| 3,5 | 2 | 5 | *Prunus spinosa* | *Prunus* | UNBROW | 11-20 |
| 3,5 | 2 | 5 | *Prunus spinosa* | *Prunus* | UNBROW | 11-20 |
| 3,5 | 2 | 5 | *Prunus spinosa* | *Prunus* | UNBROW | 11-20 |
| 3,5 | 2 | 5 | *Prunus spinosa* | *Prunus* | UNBROW | 11-20 |
| 3,5 | 2 | 5 | *Prunus spinosa* | *Prunus* | UNBROW | 11-20 |
| 3,5 | 2 | 5 | *Prunus spinosa* | *Prunus* | UNBROW | 11-20 |
| 3,5 | 2 | 5 | *Prunus spinosa* | *Prunus* | UNBROW | 11-20 |
| 3,5 | 2 | 5 | *Prunus spinosa* | *Prunus* | UNBROW | 11-20 |
| 3,5 | 2 | 5 | *Prunus spinosa* | *Prunus* | UNBROW | 11-20 |
| 3,5 | 2 | 5 | *Prunus spinosa* | *Prunus* | UNBROW | 11-20 |
| 3,5 | 2 | 5 | *Prunus spinosa* | *Prunus* | UNBROW | 11-20 |
| 3,5 | 2 | 5 | *Prunus spinosa* | *Prunus* | UNBROW | 11-20 |
| 3,5 | 2 | 5 | *Prunus spinosa* | *Prunus* | UNBROW | 11-20 |
| 3,5 | 2 | 5 | *Prunus spinosa* | *Prunus* | UNBROW | 11-20 |
| 3,5 | 2 | 5 | *Prunus spinosa* | *Prunus* | UNBROW | 11-20 |
| 3,5 | 2 | 5 | *Prunus spinosa* | *Prunus* | UNBROW | 11-20 |
| 3,5 | 2 | 5 | *Prunus spinosa* | *Prunus* | UNBROW | 11-20 |
| 3,5 | 2 | 5 | *Prunus spinosa* | *Prunus* | UNBROW | 11-20 |
| 3,5 | 2 | 5 | *Prunus spinosa* | *Prunus* | UNBROW | 11-20 |
| 3,5 | 2 | 5 | *Prunus spinosa* | *Prunus* | UNBROW | 11-20 |
| 3,5 | 2 | 5 | *Prunus spinosa* | *Prunus* | UNBROW | 11-20 |
| 3,5 | 2 | 5 | *Prunus spinosa* | *Prunus* | UNBROW | 11-20 |
| 3,5 | 2 | 5 | *Prunus spinosa* | *Prunus* | UNBROW | 11-20 |
| 3,5 | 2 | 5 | *Prunus spinosa* | *Prunus* | UNBROW | 11-20 |
| 20 | 2 | 5 | *Pinus sylvestris* | *Pinus* | UNBROW | 21-30 |
| 20 | 2 | 5 | *Pinus sylvestris* | *Pinus* | UNBROW | 21-30 |
| 20 | 2 | 5 | *Pinus sylvestris* | *Pinus* | UNBROW | 21-30 |
| 20 | 2 | 5 | *Pinus sylvestris* | *Pinus* | UNBROW | 21-30 |
| 20 | 2 | 5 | *Pinus sylvestris* | *Pinus* | UNBROW | 21-30 |
| 14,9 | 3 | 6 | *Acer negundo* | *Acer* | BROW | 0-10 |
| 4,8 | 3 | 6 | *Acer negundo* | *Acer* | BROW | 0-10 |
| 1,9 | 3 | 6 | *Acer platanoides* | *Acer* | BROW | 0-10 |
| 2,7 | 3 | 6 | *Acer platanoides* | *Acer* | BROW | 0-10 |
| 4,4 | 3 | 6 | *Acer platanoides* | *Acer* | BROW | 0-10 |
| 0,6 | 3 | 6 | *Acer platanoides* | *Acer* | BROW | 0-10 |
| 3,6 | 3 | 6 | *Acer platanoides* | *Acer* | BROW | 0-10 |
| 18,7 | 3 | 6 | *Acer negundo* | *Acer* | BROW | 0-10 |
| 5,6 | 3 | 6 | *Juglans regia* | Others | UNBROW | 21-30 |
| 6,1 | 3 | 6 | *Juglans regia* | Others | UNBROW | 21-30 |
| 7 | 3 | 6 | *Quercus robur* | *Quercus* | UNBROW | 21-30 |
| 60,3 | 3 | 6 | *Alnus glutinosa* | *Alnus* | UNBROW | 0-10 |
| 12,6 | 3 | 6 | *Alnus glutinosa* | *Alnus* | UNBROW | 0-10 |
| 23,4 | 3 | 6 | *Crataegus* sp. | *Crataegus* | BROW | 0-10 |
| 1,3 | 3 | 6 | *Salix alba* | *Salix* | UNBROW | 0-10 |
| 0,4 | 3 | 6 | *Salix alba* | *Salix* | UNBROW | 0-10 |
| 1,1 | 3 | 6 | *Salix alba* | *Salix* | UNBROW | 0-10 |
| 1,4 | 3 | 6 | *Salix alba* | *Salix* | UNBROW | 0-10 |
| 1 | 3 | 6 | *Salix alba* | *Salix* | UNBROW | 0-10 |
| 0,4 | 3 | 6 | *Acer platanoides* | *Acer* | UNBROW | 0-10 |
| 1,3 | 3 | 6 | *Acer platanoides* | *Acer* | UNBROW | 0-10 |
| 7,8 | 3 | 6 | *Sambucus nigra* | *Sambucus* | UNBROW | 0-10 |
| 4,5 | 3 | 6 | *Sambucus nigra* | *Sambucus* | UNBROW | 0-10 |
| 2,6 | 3 | 6 | *Sambucus nigra* | *Sambucus* | UNBROW | 0-10 |
| 4,9 | 3 | 6 | *Sambucus nigra* | *Sambucus* | UNBROW | 0-10 |
| 11,6 | 3 | 6 | *Prunus spinosa* | *Prunus* | UNBROW | 0-10 |
| 2,2 | 3 | 6 | *Juglans regia* | Others | UNBROW | 0-10 |
| 1,6 | 3 | 6 | *Juglans regia* | Others | UNBROW | 0-10 |
| 0,7 | 3 | 6 | *Acer platanoides* | *Acer* | BROW | 0-10 |
| 0,5 | 3 | 6 | *Acer platanoides* | *Acer* | BROW | 0-10 |
| 3,3 | 3 | 6 | *Acer platanoides* | *Acer* | BROW | 0-10 |
| 0,3 | 3 | 6 | *Acer platanoides* | *Acer* | BROW | 0-10 |
| 1 | 3 | 6 | *Acer platanoides* | *Acer* | BROW | 0-10 |
| 4,7 | 3 | 6 | *Juglans regia* | Others | UNBROW | 0-10 |
| 2 | 3 | 6 | *Juglans regia* | Others | UNBROW | 0-10 |
| 8,7 | 3 | 6 | *Juglans regia* | Others | UNBROW | 0-10 |
| 3,5 | 3 | 6 | *Juglans regia* | Others | BROW | 0-10 |
| 2 | 3 | 6 | *Alnus glutinosa* | *Alnus* | UNBROW | 0-10 |
| 0,7 | 3 | 6 | *Alnus glutinosa* | *Alnus* | UNBROW | 0-10 |
| 0,8 | 3 | 6 | *Crataegus* sp. | *Crataegus* | UNBROW | 0-10 |
| 0,3 | 3 | 6 | *Crataegus* sp. | *Crataegus* | UNBROW | 0-10 |
| 6,5 | 3 | 6 | *Acer platanoides* | *Acer* | BROW | 0-10 |
| 6,2 | 3 | 6 | *Salix alba* | *Salix* | BROW | 0-10 |
| 2,9 | 3 | 6 | *Salix alba* | *Salix* | BROW | 0-10 |
| 6,6 | 3 | 6 | *Salix alba* | *Salix* | UNBROW | 0-10 |
| 4,1 | 3 | 6 | *Sambucus nigra* | *Sambucus* | UNBROW | 0-10 |
| 14,3 | 3 | 6 | *Sambucus nigra* | *Sambucus* | UNBROW | 0-10 |
| 5 | 3 | 6 | *Alnus glutinosa* | *Alnus* | UNBROW | 0-10 |
| 58,4 | 3 | 6 | *Alnus glutinosa* | *Alnus* | UNBROW | 0-10 |
| 0,7 | 3 | 6 | *Fraxinus excelsior* | Others | UNBROW | 0-10 |
| 2 | 3 | 6 | *Prunus spinosa* | *Prunus* | UNBROW | 0-10 |
| 0,8 | 3 | 6 | *Prunus spinosa* | *Prunus* | UNBROW | 0-10 |
| 0,6 | 3 | 6 | *Sambucus nigra* | *Sambucus* | UNBROW | 0-10 |
| 3 | 3 | 6 | *Sambucus nigra* | *Sambucus* | UNBROW | 0-10 |
| 4,3 | 3 | 6 | *Sambucus nigra* | *Sambucus* | UNBROW | 0-10 |
| 6,1 | 3 | 6 | *Sambucus nigra* | *Sambucus* | UNBROW | 0-10 |
| 7 | 3 | 6 | *Sambucus nigra* | *Sambucus* | UNBROW | 0-10 |
| 2,7 | 3 | 6 | *Sambucus nigra* | *Sambucus* | UNBROW | 0-10 |
| 1 | 3 | 6 | *Sambucus nigra* | *Sambucus* | UNBROW | 0-10 |
| 3,6 | 3 | 6 | *Sambucus nigra* | *Sambucus* | UNBROW | 0-10 |
| 6,3 | 3 | 6 | *Sambucus nigra* | *Sambucus* | UNBROW | 0-10 |
| 5,5 | 3 | 6 | *Sambucus nigra* | *Sambucus* | UNBROW | 0-10 |
| 10,7 | 3 | 6 | *Sambucus nigra* | *Sambucus* | UNBROW | 0-10 |
| 2,8 | 3 | 6 | *Sambucus nigra* | *Sambucus* | UNBROW | 0-10 |
| 1,3 | 3 | 6 | *Sambucus nigra* | *Sambucus* | UNBROW | 0-10 |
| 3,3 | 3 | 6 | *Sambucus nigra* | *Sambucus* | UNBROW | 0-10 |
| 6,7 | 3 | 6 | *Sambucus nigra* | *Sambucus* | UNBROW | 0-10 |
| 10,9 | 3 | 6 | *Sambucus nigra* | *Sambucus* | UNBROW | 0-10 |
| 6,4 | 3 | 6 | *Sambucus nigra* | *Sambucus* | UNBROW | 0-10 |
| 5,3 | 3 | 6 | *Juglans regia* | Others | UNBROW | 11-20 |
| 58,6 | 3 | 6 | *Alnus glutinosa* | *Alnus* | UNBROW | 0-10 |
| 2,9 | 3 | 6 | *Sambucus nigra* | *Sambucus* | UNBROW | 0-10 |
| 2,3 | 3 | 6 | *Sambucus nigra* | *Sambucus* | UNBROW | 21-30 |
| 7,3 | 3 | 6 | *Sambucus nigra* | *Sambucus* | UNBROW | 0-10 |
| 0,9 | 3 | 6 | *Acer negundo* | *Acer* | UNBROW | 0-10 |
| 2,3 | 3 | 6 | *Quercus robur* | *Quercus* | UNBROW | 21-30 |
| 4,4 | 3 | 6 | *Crataegus* sp. | *Crataegus* | UNBROW | 0-10 |
| 12,2 | 3 | 6 | *Crataegus* sp. | *Crataegus* | UNBROW | 0-10 |
| 4,5 | 3 | 6 | *Crataegus* sp. | *Crataegus* | UNBROW | 0-10 |
| 2,6 | 3 | 6 | *Crataegus* sp. | *Crataegus* | UNBROW | 0-10 |
| 1,6 | 3 | 6 | *Crataegus* sp. | *Crataegus* | UNBROW | 11-20 |
| 2,4 | 3 | 6 | *Crataegus* sp. | *Crataegus* | UNBROW | 11-20 |
| 4,1 | 3 | 6 | *Crataegus* sp. | *Crataegus* | UNBROW | 11-20 |
| 3,7 | 3 | 6 | *Crataegus* sp. | *Crataegus* | UNBROW | 11-20 |
| 4,4 | 3 | 6 | *Crataegus* sp. | *Crataegus* | UNBROW | 11-20 |
| 3,6 | 3 | 6 | *Crataegus* sp. | *Crataegus* | UNBROW | 11-20 |
| 4,1 | 3 | 6 | *Quercus robur* | *Quercus* | UNBROW | 11-20 |
| 2,9 | 3 | 6 | *Quercus robur* | *Quercus* | UNBROW | 11-20 |
| 3,9 | 3 | 6 | *Quercus robur* | *Quercus* | UNBROW | 11-20 |
| 1,8 | 3 | 6 | *Crataegus* sp. | *Crataegus* | UNBROW | 11-20 |
| 6,6 | 3 | 6 | *Quercus rubra* | *Quercus* | UNBROW | 11-20 |
| 4,4 | 3 | 6 | *Quercus rubra* | *Quercus* | UNBROW | 11-20 |
| 13,1 | 3 | 6 | *Quercus rubra* | *Quercus* | UNBROW | 11-20 |
| 10,3 | 3 | 6 | *Alnus glutinosa* | *Alnus* | UNBROW | 11-20 |
| 3,6 | 3 | 6 | *Alnus glutinosa* | *Alnus* | UNBROW | 11-20 |
| 1 | 3 | 6 | *Alnus glutinosa* | *Alnus* | UNBROW | 11-20 |
| 1,5 | 3 | 6 | *Alnus glutinosa* | *Alnus* | UNBROW | 11-20 |
| 1,3 | 3 | 6 | *Alnus glutinosa* | *Alnus* | UNBROW | 11-20 |
| 10,8 | 3 | 6 | *Alnus glutinosa* | *Alnus* | UNBROW | 11-20 |
| 1,6 | 3 | 6 | *Quercus robur* | *Quercus* | UNBROW | 11-20 |
| 7,2 | 3 | 6 | *Quercus robur* | *Quercus* | UNBROW | 11-20 |
| 2 | 3 | 6 | *Quercus robur* | *Quercus* | UNBROW | 11-20 |
| 2,1 | 3 | 6 | *Quercus robur* | *Quercus* | UNBROW | 11-20 |
| 1,5 | 3 | 6 | *Salix alba* | *Salix* | BROW | 0-10 |
| 1,6 | 3 | 6 | *Salix alba* | *Salix* | BROW | 0-10 |
| 1,8 | 3 | 6 | *Salix alba* | *Salix* | BROW | 0-10 |
| 1,6 | 3 | 6 | *Salix alba* | *Salix* | BROW | 0-10 |
| 1,4 | 3 | 6 | *Salix alba* | *Salix* | BROW | 0-10 |
| 1,3 | 3 | 6 | *Salix alba* | *Salix* | BROW | 0-10 |
| 1,7 | 3 | 6 | *Salix alba* | *Salix* | BROW | 0-10 |
| 1,1 | 3 | 6 | *Salix alba* | *Salix* | BROW | 0-10 |
| 2 | 3 | 6 | *Salix alba* | *Salix* | BROW | 0-10 |
| 1,2 | 3 | 6 | *Salix alba* | *Salix* | BROW | 0-10 |
| 1,2 | 3 | 6 | *Salix alba* | *Salix* | BROW | 0-10 |
| 1,4 | 3 | 6 | *Salix alba* | *Salix* | BROW | 0-10 |
| 1,7 | 3 | 6 | *Salix alba* | *Salix* | BROW | 0-10 |
| 1,8 | 3 | 6 | *Salix alba* | *Salix* | BROW | 0-10 |
| 1,9 | 3 | 6 | *Salix alba* | *Salix* | BROW | 0-10 |
| 1,5 | 3 | 6 | *Salix alba* | *Salix* | BROW | 0-10 |
| 1,5 | 3 | 6 | *Salix alba* | *Salix* | BROW | 0-10 |
| 1,6 | 3 | 6 | *Salix alba* | *Salix* | BROW | 0-10 |
| 1,7 | 3 | 6 | *Salix alba* | *Salix* | BROW | 0-10 |
| 1,4 | 3 | 6 | *Salix alba* | *Salix* | BROW | 0-10 |
| 1,3 | 3 | 6 | *Salix alba* | *Salix* | BROW | 0-10 |
| 1,5 | 3 | 6 | *Salix alba* | *Salix* | BROW | 0-10 |
| 1,6 | 3 | 6 | *Salix alba* | *Salix* | BROW | 0-10 |
| 1,4 | 3 | 6 | *Salix alba* | *Salix* | BROW | 0-10 |
| 1,7 | 3 | 6 | *Salix alba* | *Salix* | BROW | 0-10 |
| 1,7 | 3 | 6 | *Salix alba* | *Salix* | BROW | 0-10 |
| 1,8 | 3 | 6 | *Salix alba* | *Salix* | BROW | 0-10 |
| 1,8 | 3 | 6 | *Salix alba* | *Salix* | BROW | 0-10 |
| 1,1 | 3 | 6 | *Salix alba* | *Salix* | BROW | 0-10 |
| 1,2 | 3 | 6 | *Salix alba* | *Salix* | BROW | 0-10 |
| 1,3 | 3 | 6 | *Salix alba* | *Salix* | BROW | 0-10 |
| 1,4 | 3 | 6 | *Salix alba* | *Salix* | BROW | 0-10 |
| 1,3 | 3 | 6 | *Salix alba* | *Salix* | BROW | 0-10 |
| 1,4 | 3 | 6 | *Salix alba* | *Salix* | BROW | 0-10 |
| 1,5 | 3 | 6 | *Salix alba* | *Salix* | BROW | 0-10 |
| 1,5 | 3 | 6 | *Salix alba* | *Salix* | BROW | 0-10 |
| 1,5 | 3 | 6 | *Salix alba* | *Salix* | BROW | 0-10 |
| 1,3 | 3 | 6 | *Salix alba* | *Salix* | BROW | 0-10 |
| 1,3 | 3 | 6 | *Salix alba* | *Salix* | BROW | 0-10 |
| 1,6 | 3 | 6 | *Salix alba* | *Salix* | BROW | 0-10 |
| 1,7 | 3 | 6 | *Salix alba* | *Salix* | BROW | 0-10 |
| 1,7 | 3 | 6 | *Salix alba* | *Salix* | BROW | 0-10 |
| 1,7 | 3 | 6 | *Salix alba* | *Salix* | BROW | 0-10 |
| 1,8 | 3 | 6 | *Salix alba* | *Salix* | BROW | 0-10 |
| 1,7 | 3 | 6 | *Salix alba* | *Salix* | BROW | 0-10 |
| 1,9 | 3 | 6 | *Salix alba* | *Salix* | BROW | 0-10 |
| 1,6 | 3 | 6 | *Salix alba* | *Salix* | BROW | 0-10 |
| 1,5 | 3 | 6 | *Salix alba* | *Salix* | BROW | 0-10 |
| 1,2 | 3 | 6 | *Salix alba* | *Salix* | BROW | 0-10 |
| 1,3 | 3 | 6 | *Salix alba* | *Salix* | BROW | 0-10 |
| 1,6 | 3 | 6 | *Salix alba* | *Salix* | BROW | 0-10 |
| 1,7 | 3 | 6 | *Salix alba* | *Salix* | BROW | 0-10 |
| 1,8 | 3 | 6 | *Salix alba* | *Salix* | BROW | 0-10 |
| 1,9 | 3 | 6 | *Salix alba* | *Salix* | BROW | 0-10 |
| 1,4 | 3 | 6 | *Salix alba* | *Salix* | BROW | 0-10 |
| 1,4 | 3 | 6 | *Salix alba* | *Salix* | BROW | 0-10 |
| 1,4 | 3 | 6 | *Salix alba* | *Salix* | BROW | 0-10 |
| 1,6 | 3 | 6 | *Salix alba* | *Salix* | BROW | 0-10 |
| 1,6 | 3 | 6 | *Salix alba* | *Salix* | BROW | 0-10 |
| 1,8 | 3 | 6 | *Salix alba* | *Salix* | BROW | 0-10 |
| 1,5 | 3 | 6 | *Salix alba* | *Salix* | BROW | 0-10 |
| 1,5 | 3 | 6 | *Salix alba* | *Salix* | BROW | 0-10 |
| 1,4 | 3 | 6 | *Salix alba* | *Salix* | BROW | 0-10 |
| 1,6 | 3 | 6 | *Salix alba* | *Salix* | BROW | 0-10 |
| 1,9 | 3 | 6 | *Salix alba* | *Salix* | BROW | 0-10 |
| 1,8 | 3 | 6 | *Salix alba* | *Salix* | BROW | 0-10 |
| 1,7 | 3 | 6 | *Salix alba* | *Salix* | BROW | 0-10 |
| 1,5 | 3 | 6 | *Salix alba* | *Salix* | BROW | 0-10 |
| 1,2 | 3 | 6 | *Salix alba* | *Salix* | BROW | 0-10 |
| 1,3 | 3 | 6 | *Salix alba* | *Salix* | BROW | 0-10 |
| 1,2 | 3 | 6 | *Salix alba* | *Salix* | BROW | 0-10 |
| 1,3 | 3 | 6 | *Salix alba* | *Salix* | BROW | 0-10 |
| 1,4 | 3 | 6 | *Salix alba* | *Salix* | BROW | 0-10 |
| 1,3 | 3 | 6 | *Salix alba* | *Salix* | BROW | 0-10 |
| 1,4 | 3 | 6 | *Salix alba* | *Salix* | BROW | 0-10 |
| 1,5 | 3 | 6 | *Salix alba* | *Salix* | BROW | 0-10 |
| 1,5 | 3 | 6 | *Salix alba* | *Salix* | BROW | 0-10 |
| 1,7 | 3 | 6 | *Salix alba* | *Salix* | BROW | 0-10 |
| 1,7 | 3 | 6 | *Salix alba* | *Salix* | BROW | 0-10 |
| 1,8 | 3 | 6 | *Salix alba* | *Salix* | BROW | 0-10 |
| 1,7 | 3 | 6 | *Salix alba* | *Salix* | BROW | 0-10 |
| 1,9 | 3 | 6 | *Salix alba* | *Salix* | BROW | 0-10 |
| 1,6 | 3 | 6 | *Salix alba* | *Salix* | BROW | 0-10 |
| 1,5 | 3 | 6 | *Salix alba* | *Salix* | BROW | 0-10 |
| 1,2 | 3 | 6 | *Salix alba* | *Salix* | BROW | 0-10 |
| 1,3 | 3 | 6 | *Salix alba* | *Salix* | BROW | 0-10 |
| 1,6 | 3 | 6 | *Salix alba* | *Salix* | BROW | 0-10 |
| 1,7 | 3 | 6 | *Salix alba* | *Salix* | BROW | 0-10 |
| 1,8 | 3 | 6 | *Salix alba* | *Salix* | BROW | 0-10 |
| 1,9 | 3 | 6 | *Salix alba* | *Salix* | BROW | 0-10 |
| 1,4 | 3 | 6 | *Salix alba* | *Salix* | BROW | 0-10 |
| 1,4 | 3 | 6 | *Salix alba* | *Salix* | BROW | 0-10 |
| 1,4 | 3 | 6 | *Salix alba* | *Salix* | BROW | 0-10 |
| 1,6 | 3 | 6 | *Salix alba* | *Salix* | BROW | 0-10 |
| 1,6 | 3 | 6 | *Salix alba* | *Salix* | BROW | 0-10 |
| 1,8 | 3 | 6 | *Salix alba* | *Salix* | BROW | 0-10 |
| 1,5 | 3 | 6 | *Salix alba* | *Salix* | BROW | 0-10 |
| 1,5 | 3 | 6 | *Salix alba* | *Salix* | BROW | 0-10 |
| 1,4 | 3 | 6 | *Salix alba* | *Salix* | BROW | 0-10 |
| 1,6 | 3 | 6 | *Salix alba* | *Salix* | BROW | 0-10 |
| 1,9 | 3 | 6 | *Salix alba* | *Salix* | BROW | 0-10 |
| 1,8 | 3 | 6 | *Salix alba* | *Salix* | BROW | 0-10 |
| 1,7 | 3 | 6 | *Salix alba* | *Salix* | BROW | 0-10 |
| 1,5 | 3 | 6 | *Salix alba* | *Salix* | BROW | 0-10 |
| 1,2 | 3 | 6 | *Salix alba* | *Salix* | BROW | 0-10 |
| 1,3 | 3 | 6 | *Salix alba* | *Salix* | BROW | 0-10 |
| 1,2 | 3 | 6 | *Salix alba* | *Salix* | BROW | 0-10 |
| 1,3 | 3 | 6 | *Salix alba* | *Salix* | BROW | 0-10 |
| 1,4 | 3 | 6 | *Salix alba* | *Salix* | BROW | 0-10 |
| 1,3 | 3 | 6 | *Salix alba* | *Salix* | BROW | 0-10 |
| 1,4 | 3 | 6 | *Salix alba* | *Salix* | BROW | 0-10 |
| 1,5 | 3 | 6 | *Salix alba* | *Salix* | BROW | 0-10 |
| 1,5 | 3 | 6 | *Salix alba* | *Salix* | BROW | 0-10 |
| 1,3 | 3 | 6 | *Salix alba* | *Salix* | BROW | 0-10 |
| 1,3 | 3 | 6 | *Salix alba* | *Salix* | BROW | 0-10 |
| 1,3 | 3 | 6 | *Salix alba* | *Salix* | BROW | 0-10 |
| 1,3 | 3 | 6 | *Salix alba* | *Salix* | BROW | 0-10 |
| 1,2 | 3 | 6 | *Salix alba* | *Salix* | BROW | 0-10 |
| 1,2 | 3 | 6 | *Salix alba* | *Salix* | BROW | 0-10 |
| 1,5 | 3 | 6 | *Salix alba* | *Salix* | BROW | 0-10 |
| 1,5 | 3 | 6 | *Salix alba* | *Salix* | BROW | 0-10 |
| 1,5 | 3 | 6 | *Salix alba* | *Salix* | BROW | 0-10 |
| 1,5 | 3 | 6 | *Salix alba* | *Salix* | BROW | 0-10 |
| 1,5 | 3 | 6 | *Salix alba* | *Salix* | BROW | 0-10 |
| 1,5 | 3 | 6 | *Salix alba* | *Salix* | BROW | 0-10 |
| 1,6 | 3 | 6 | *Salix alba* | *Salix* | BROW | 0-10 |
| 1,7 | 3 | 6 | *Salix alba* | *Salix* | BROW | 0-10 |
| 1,7 | 3 | 6 | *Salix alba* | *Salix* | BROW | 0-10 |
| 1,6 | 3 | 6 | *Salix alba* | *Salix* | BROW | 0-10 |
| 1,5 | 3 | 6 | *Salix alba* | *Salix* | BROW | 0-10 |
| 1,5 | 3 | 6 | *Salix alba* | *Salix* | BROW | 0-10 |
| 1,6 | 3 | 6 | *Salix alba* | *Salix* | BROW | 0-10 |
| 1,8 | 3 | 6 | *Salix alba* | *Salix* | BROW | 0-10 |
| 1,6 | 3 | 6 | *Salix alba* | *Salix* | BROW | 0-10 |
| 1,4 | 3 | 6 | *Salix alba* | *Salix* | BROW | 0-10 |
| 1,3 | 3 | 6 | *Salix alba* | *Salix* | BROW | 0-10 |
| 1,7 | 3 | 6 | *Salix alba* | *Salix* | BROW | 0-10 |
| 1,1 | 3 | 6 | *Salix alba* | *Salix* | BROW | 0-10 |
| 2 | 3 | 6 | *Salix alba* | *Salix* | BROW | 0-10 |
| 1,2 | 3 | 6 | *Salix alba* | *Salix* | BROW | 0-10 |
| 1,2 | 3 | 6 | *Salix alba* | *Salix* | BROW | 0-10 |
| 1,4 | 3 | 6 | *Salix alba* | *Salix* | BROW | 0-10 |
| 1,7 | 3 | 6 | *Salix alba* | *Salix* | BROW | 0-10 |
| 1,8 | 3 | 6 | *Salix alba* | *Salix* | BROW | 0-10 |
| 1,9 | 3 | 6 | *Salix alba* | *Salix* | BROW | 0-10 |
| 1,5 | 3 | 6 | *Salix alba* | *Salix* | BROW | 0-10 |
| 1,5 | 3 | 6 | *Salix alba* | *Salix* | BROW | 0-10 |
| 1,6 | 3 | 6 | *Salix alba* | *Salix* | BROW | 0-10 |
| 1,7 | 3 | 6 | *Salix alba* | *Salix* | BROW | 0-10 |
| 1,4 | 3 | 6 | *Salix alba* | *Salix* | BROW | 0-10 |
| 1,3 | 3 | 6 | *Salix alba* | *Salix* | BROW | 0-10 |
| 1,5 | 3 | 6 | *Salix alba* | *Salix* | BROW | 0-10 |
| 1,6 | 3 | 6 | *Salix alba* | *Salix* | BROW | 0-10 |
| 1,4 | 3 | 6 | *Salix alba* | *Salix* | BROW | 0-10 |
| 1,7 | 3 | 6 | *Salix alba* | *Salix* | BROW | 0-10 |
| 1,7 | 3 | 6 | *Salix alba* | *Salix* | BROW | 0-10 |
| 1,8 | 3 | 6 | *Salix alba* | *Salix* | BROW | 0-10 |
| 1,8 | 3 | 6 | *Salix alba* | *Salix* | BROW | 0-10 |
| 1,1 | 3 | 6 | *Salix alba* | *Salix* | BROW | 0-10 |
| 1,2 | 3 | 6 | *Salix alba* | *Salix* | BROW | 0-10 |
| 1,3 | 3 | 6 | *Salix alba* | *Salix* | BROW | 0-10 |
| 1,4 | 3 | 6 | *Salix alba* | *Salix* | BROW | 0-10 |
| 1,3 | 3 | 6 | *Salix alba* | *Salix* | BROW | 0-10 |
| 1,4 | 3 | 6 | *Salix alba* | *Salix* | BROW | 0-10 |
| 1,5 | 3 | 6 | *Salix alba* | *Salix* | BROW | 0-10 |
| 1,5 | 3 | 6 | *Salix alba* | *Salix* | BROW | 0-10 |
| 1,5 | 3 | 6 | *Salix alba* | *Salix* | BROW | 0-10 |
| 1,3 | 3 | 6 | *Salix alba* | *Salix* | BROW | 0-10 |
| 1,3 | 3 | 6 | *Salix alba* | *Salix* | BROW | 0-10 |
| 1,6 | 3 | 6 | *Salix alba* | *Salix* | BROW | 0-10 |
| 1,7 | 3 | 6 | *Salix alba* | *Salix* | BROW | 0-10 |
| 1,7 | 3 | 6 | *Salix alba* | *Salix* | BROW | 0-10 |
| 1,7 | 3 | 6 | *Salix alba* | *Salix* | BROW | 0-10 |
| 1,8 | 3 | 6 | *Salix alba* | *Salix* | BROW | 0-10 |
| 1,7 | 3 | 6 | *Salix alba* | *Salix* | BROW | 0-10 |
| 1,9 | 3 | 6 | *Salix alba* | *Salix* | BROW | 0-10 |
| 1,6 | 3 | 6 | *Salix alba* | *Salix* | BROW | 0-10 |
| 1,5 | 3 | 6 | *Salix alba* | *Salix* | BROW | 0-10 |
| 1,2 | 3 | 6 | *Salix alba* | *Salix* | BROW | 0-10 |
| 1,3 | 3 | 6 | *Salix alba* | *Salix* | BROW | 0-10 |
| 1,6 | 3 | 6 | *Salix alba* | *Salix* | BROW | 0-10 |
| 1,7 | 3 | 6 | *Salix alba* | *Salix* | BROW | 0-10 |
| 1,8 | 3 | 6 | *Salix alba* | *Salix* | BROW | 0-10 |
| 1,9 | 3 | 6 | *Salix alba* | *Salix* | BROW | 0-10 |
| 1,4 | 3 | 6 | *Salix alba* | *Salix* | BROW | 0-10 |
| 1,4 | 3 | 6 | *Salix alba* | *Salix* | BROW | 0-10 |
| 1,4 | 3 | 6 | *Salix alba* | *Salix* | BROW | 0-10 |
| 1,6 | 3 | 6 | *Salix alba* | *Salix* | BROW | 0-10 |
| 1,6 | 3 | 6 | *Salix alba* | *Salix* | BROW | 0-10 |
| 1,8 | 3 | 6 | *Salix alba* | *Salix* | BROW | 0-10 |
| 1,5 | 3 | 6 | *Salix alba* | *Salix* | BROW | 0-10 |
| 1,5 | 3 | 6 | *Salix alba* | *Salix* | BROW | 0-10 |
| 1,4 | 3 | 6 | *Salix alba* | *Salix* | BROW | 0-10 |
| 1,6 | 3 | 6 | *Salix alba* | *Salix* | BROW | 0-10 |
| 1,9 | 3 | 6 | *Salix alba* | *Salix* | BROW | 0-10 |
| 1,8 | 3 | 6 | *Salix alba* | *Salix* | BROW | 0-10 |
| 1,7 | 3 | 6 | *Salix alba* | *Salix* | BROW | 0-10 |
| 1,5 | 3 | 6 | *Salix alba* | *Salix* | BROW | 0-10 |
| 1,2 | 3 | 6 | *Salix alba* | *Salix* | BROW | 0-10 |
| 1,3 | 3 | 6 | *Salix alba* | *Salix* | BROW | 0-10 |
| 1,2 | 3 | 6 | *Salix alba* | *Salix* | BROW | 0-10 |
| 1,3 | 3 | 6 | *Salix alba* | *Salix* | BROW | 0-10 |
| 1,4 | 3 | 6 | *Salix alba* | *Salix* | BROW | 0-10 |
| 1,3 | 3 | 6 | *Salix alba* | *Salix* | BROW | 0-10 |
| 1,4 | 3 | 6 | *Salix alba* | *Salix* | BROW | 0-10 |
| 1,5 | 3 | 6 | *Salix alba* | *Salix* | BROW | 0-10 |
| 1,5 | 3 | 6 | *Salix alba* | *Salix* | BROW | 0-10 |
| 1,7 | 3 | 6 | *Salix alba* | *Salix* | BROW | 0-10 |
| 1,7 | 3 | 6 | *Salix alba* | *Salix* | BROW | 0-10 |
| 1,8 | 3 | 6 | *Salix alba* | *Salix* | BROW | 0-10 |
| 1,7 | 3 | 6 | *Salix alba* | *Salix* | BROW | 0-10 |
| 1,9 | 3 | 6 | *Salix alba* | *Salix* | BROW | 0-10 |
| 1,6 | 3 | 6 | *Salix alba* | *Salix* | BROW | 0-10 |
| 1,5 | 3 | 6 | *Salix alba* | *Salix* | BROW | 0-10 |
| 1,2 | 3 | 6 | *Salix alba* | *Salix* | BROW | 0-10 |
| 1,3 | 3 | 6 | *Salix alba* | *Salix* | BROW | 0-10 |
| 1,6 | 3 | 6 | *Salix alba* | *Salix* | BROW | 0-10 |
| 1,7 | 3 | 6 | *Salix alba* | *Salix* | BROW | 0-10 |
| 1,8 | 3 | 6 | *Salix alba* | *Salix* | BROW | 0-10 |
| 1,9 | 3 | 6 | *Salix alba* | *Salix* | BROW | 0-10 |
| 1,4 | 3 | 6 | *Salix alba* | *Salix* | BROW | 0-10 |
| 1,4 | 3 | 6 | *Salix alba* | *Salix* | BROW | 0-10 |
| 1,4 | 3 | 6 | *Salix alba* | *Salix* | BROW | 0-10 |
| 1,6 | 3 | 6 | *Salix alba* | *Salix* | BROW | 0-10 |
| 1,6 | 3 | 6 | *Salix alba* | *Salix* | BROW | 0-10 |
| 1,8 | 3 | 6 | *Salix alba* | *Salix* | BROW | 0-10 |
| 1,5 | 3 | 6 | *Salix alba* | *Salix* | BROW | 0-10 |
| 1,5 | 3 | 6 | *Salix alba* | *Salix* | BROW | 0-10 |
| 1,4 | 3 | 6 | *Salix alba* | *Salix* | BROW | 0-10 |
| 1,6 | 3 | 6 | *Salix alba* | *Salix* | BROW | 0-10 |
| 1,9 | 3 | 6 | *Salix alba* | *Salix* | BROW | 0-10 |
| 1,8 | 3 | 6 | *Salix alba* | *Salix* | BROW | 0-10 |
| 1,7 | 3 | 6 | *Salix alba* | *Salix* | BROW | 0-10 |
| 1,5 | 3 | 6 | *Salix alba* | *Salix* | BROW | 0-10 |
| 1,2 | 3 | 6 | *Salix alba* | *Salix* | BROW | 0-10 |
| 1,3 | 3 | 6 | *Salix alba* | *Salix* | BROW | 0-10 |
| 1,2 | 3 | 6 | *Salix alba* | *Salix* | BROW | 0-10 |
| 1,3 | 3 | 6 | *Salix alba* | *Salix* | BROW | 0-10 |
| 1,4 | 3 | 6 | *Salix alba* | *Salix* | BROW | 0-10 |
| 1,3 | 3 | 6 | *Salix alba* | *Salix* | BROW | 0-10 |
| 1,4 | 3 | 6 | *Salix alba* | *Salix* | BROW | 0-10 |
| 1,5 | 3 | 6 | *Salix alba* | *Salix* | BROW | 0-10 |
| 1,5 | 3 | 6 | *Salix alba* | *Salix* | BROW | 0-10 |
| 1,3 | 3 | 6 | *Salix alba* | *Salix* | BROW | 0-10 |
| 1,3 | 3 | 6 | *Salix alba* | *Salix* | BROW | 0-10 |
| 1,3 | 3 | 6 | *Salix alba* | *Salix* | BROW | 0-10 |
| 1,3 | 3 | 6 | *Salix alba* | *Salix* | BROW | 0-10 |
| 1,2 | 3 | 6 | *Salix alba* | *Salix* | BROW | 0-10 |
| 1,2 | 3 | 6 | *Salix alba* | *Salix* | BROW | 0-10 |
| 1,5 | 3 | 6 | *Salix alba* | *Salix* | BROW | 0-10 |
| 1,5 | 3 | 6 | *Salix alba* | *Salix* | BROW | 0-10 |
| 1,5 | 3 | 6 | *Salix alba* | *Salix* | BROW | 0-10 |
| 1,5 | 3 | 6 | *Salix alba* | *Salix* | BROW | 0-10 |
| 1,5 | 3 | 6 | *Salix alba* | *Salix* | BROW | 0-10 |
| 1,5 | 3 | 6 | *Salix alba* | *Salix* | BROW | 0-10 |
| 1,6 | 3 | 6 | *Salix alba* | *Salix* | BROW | 0-10 |
| 1,7 | 3 | 6 | *Salix alba* | *Salix* | BROW | 0-10 |
| 1,7 | 3 | 6 | *Salix alba* | *Salix* | BROW | 0-10 |
| 1,6 | 3 | 6 | *Salix alba* | *Salix* | BROW | 0-10 |
| 1,5 | 3 | 6 | *Salix alba* | *Salix* | BROW | 0-10 |
| 1,2 | 3 | 6 | *Salix alba* | *Salix* | BROW | 0-10 |
| 1,2 | 3 | 6 | *Salix alba* | *Salix* | BROW | 0-10 |
| 1,3 | 3 | 6 | *Salix alba* | *Salix* | BROW | 0-10 |
| 1,3 | 3 | 6 | *Salix alba* | *Salix* | BROW | 0-10 |
| 1,2 | 3 | 6 | *Salix alba* | *Salix* | BROW | 0-10 |
| 1,3 | 3 | 6 | *Salix alba* | *Salix* | BROW | 0-10 |
| 1,3 | 3 | 6 | *Salix alba* | *Salix* | BROW | 0-10 |
| 1,3 | 3 | 6 | *Salix alba* | *Salix* | BROW | 0-10 |
| 1,3 | 3 | 6 | *Salix alba* | *Salix* | BROW | 0-10 |
| 1,3 | 3 | 6 | *Salix alba* | *Salix* | BROW | 0-10 |
| 1,4 | 3 | 6 | *Salix alba* | *Salix* | BROW | 0-10 |
| 1,4 | 3 | 6 | *Salix alba* | *Salix* | BROW | 0-10 |
| 1,4 | 3 | 6 | *Salix alba* | *Salix* | BROW | 0-10 |
| 1,4 | 3 | 6 | *Salix alba* | *Salix* | BROW | 0-10 |
| 1,4 | 3 | 6 | *Salix alba* | *Salix* | BROW | 0-10 |
| 1,4 | 3 | 6 | *Salix alba* | *Salix* | BROW | 0-10 |
| 1,4 | 3 | 6 | *Salix alba* | *Salix* | BROW | 0-10 |
| 1,5 | 3 | 6 | *Salix alba* | *Salix* | BROW | 0-10 |
| 1,5 | 3 | 6 | *Salix alba* | *Salix* | BROW | 0-10 |
| 1,5 | 3 | 6 | *Salix alba* | *Salix* | BROW | 0-10 |
| 1,5 | 3 | 6 | *Salix alba* | *Salix* | BROW | 0-10 |
| 1,5 | 3 | 6 | *Salix alba* | *Salix* | BROW | 0-10 |
| 1,5 | 3 | 6 | *Salix alba* | *Salix* | BROW | 0-10 |
| 1,5 | 3 | 6 | *Salix alba* | *Salix* | BROW | 0-10 |
| 1,5 | 3 | 6 | *Salix alba* | *Salix* | BROW | 0-10 |
| 1,5 | 3 | 6 | *Salix alba* | *Salix* | BROW | 0-10 |
| 1,5 | 3 | 6 | *Salix alba* | *Salix* | BROW | 0-10 |
| 1,5 | 3 | 6 | *Salix alba* | *Salix* | BROW | 0-10 |
| 1,5 | 3 | 6 | *Salix alba* | *Salix* | BROW | 0-10 |
| 1,4 | 3 | 6 | *Salix alba* | *Salix* | BROW | 0-10 |
| 1,6 | 3 | 6 | *Salix alba* | *Salix* | BROW | 0-10 |
| 1,9 | 3 | 6 | *Salix alba* | *Salix* | BROW | 0-10 |
| 1,8 | 3 | 6 | *Salix alba* | *Salix* | BROW | 0-10 |
| 1,7 | 3 | 6 | *Salix alba* | *Salix* | BROW | 0-10 |
| 1,5 | 3 | 6 | *Salix alba* | *Salix* | BROW | 0-10 |
| 1,2 | 3 | 6 | *Salix alba* | *Salix* | BROW | 0-10 |
| 1,3 | 3 | 6 | *Salix alba* | *Salix* | BROW | 0-10 |
| 1,2 | 3 | 6 | *Salix alba* | *Salix* | BROW | 0-10 |
| 1,3 | 3 | 6 | *Salix alba* | *Salix* | BROW | 0-10 |
| 1,4 | 3 | 6 | *Salix alba* | *Salix* | BROW | 0-10 |
| 1,3 | 3 | 6 | *Salix alba* | *Salix* | BROW | 0-10 |
| 1,4 | 3 | 6 | *Salix alba* | *Salix* | BROW | 0-10 |
| 1,5 | 3 | 6 | *Salix alba* | *Salix* | BROW | 0-10 |
| 1,5 | 3 | 6 | *Salix alba* | *Salix* | BROW | 0-10 |
| 1,3 | 3 | 6 | *Salix alba* | *Salix* | BROW | 0-10 |
| 1,3 | 3 | 6 | *Salix alba* | *Salix* | BROW | 0-10 |
| 1,3 | 3 | 6 | *Salix alba* | *Salix* | BROW | 0-10 |
| 1,3 | 3 | 6 | *Salix alba* | *Salix* | BROW | 0-10 |
| 1,2 | 3 | 6 | *Salix alba* | *Salix* | BROW | 0-10 |
| 1,2 | 3 | 6 | *Salix alba* | *Salix* | BROW | 0-10 |
| 1,5 | 3 | 6 | *Salix alba* | *Salix* | BROW | 0-10 |
| 1,5 | 3 | 6 | *Salix alba* | *Salix* | BROW | 0-10 |
| 1,5 | 3 | 6 | *Salix alba* | *Salix* | BROW | 0-10 |
| 1,5 | 3 | 6 | *Salix alba* | *Salix* | BROW | 0-10 |
| 1,5 | 3 | 6 | *Salix alba* | *Salix* | BROW | 0-10 |
| 1,5 | 3 | 6 | *Salix alba* | *Salix* | BROW | 0-10 |
| 1,6 | 3 | 6 | *Salix alba* | *Salix* | BROW | 0-10 |
| 1,7 | 3 | 6 | *Salix alba* | *Salix* | BROW | 0-10 |
| 1,7 | 3 | 6 | *Salix alba* | *Salix* | BROW | 0-10 |
| 1,6 | 3 | 6 | *Salix alba* | *Salix* | BROW | 0-10 |
| 1,5 | 3 | 6 | *Salix alba* | *Salix* | BROW | 0-10 |
| 1,2 | 3 | 6 | *Salix alba* | *Salix* | BROW | 0-10 |
| 1,2 | 3 | 6 | *Salix alba* | *Salix* | BROW | 0-10 |
| 1,3 | 3 | 6 | *Salix alba* | *Salix* | BROW | 0-10 |
| 1,3 | 3 | 6 | *Salix alba* | *Salix* | BROW | 0-10 |
| 1,2 | 3 | 6 | *Salix alba* | *Salix* | BROW | 0-10 |
| 1,3 | 3 | 6 | *Salix alba* | *Salix* | BROW | 0-10 |
| 1,3 | 3 | 6 | *Salix alba* | *Salix* | BROW | 0-10 |
| 1,3 | 3 | 6 | *Salix alba* | *Salix* | BROW | 0-10 |
| 1,3 | 3 | 6 | *Salix alba* | *Salix* | BROW | 0-10 |
| 1,3 | 3 | 6 | *Salix alba* | *Salix* | BROW | 0-10 |
| 1,4 | 3 | 6 | *Salix alba* | *Salix* | BROW | 0-10 |
| 1,4 | 3 | 6 | *Salix alba* | *Salix* | BROW | 0-10 |
| 1,4 | 3 | 6 | *Salix alba* | *Salix* | BROW | 0-10 |
| 1,4 | 3 | 6 | *Salix alba* | *Salix* | BROW | 0-10 |
| 1,4 | 3 | 6 | *Salix alba* | *Salix* | BROW | 0-10 |
| 1,4 | 3 | 6 | *Salix alba* | *Salix* | BROW | 0-10 |
| 1,4 | 3 | 6 | *Salix alba* | *Salix* | BROW | 0-10 |
| 1,5 | 3 | 6 | *Salix alba* | *Salix* | BROW | 0-10 |
| 1,5 | 3 | 6 | *Salix alba* | *Salix* | BROW | 0-10 |
| 1,5 | 3 | 6 | *Salix alba* | *Salix* | BROW | 0-10 |
| 1,5 | 3 | 6 | *Salix alba* | *Salix* | BROW | 0-10 |
| 1,4 | 3 | 6 | *Salix alba* | *Salix* | BROW | 0-10 |
| 1,6 | 3 | 6 | *Salix alba* | *Salix* | BROW | 0-10 |
| 1,9 | 3 | 6 | *Salix alba* | *Salix* | BROW | 0-10 |
| 1,8 | 3 | 6 | *Salix alba* | *Salix* | BROW | 0-10 |
| 1,7 | 3 | 6 | *Salix alba* | *Salix* | BROW | 0-10 |
| 1,5 | 3 | 6 | *Salix alba* | *Salix* | BROW | 0-10 |
| 1,2 | 3 | 6 | *Salix alba* | *Salix* | BROW | 0-10 |
| 1,3 | 3 | 6 | *Salix alba* | *Salix* | BROW | 0-10 |
| 1,2 | 3 | 6 | *Salix alba* | *Salix* | BROW | 0-10 |
| 1,3 | 3 | 6 | *Salix alba* | *Salix* | BROW | 0-10 |
| 1,4 | 3 | 6 | *Salix alba* | *Salix* | BROW | 0-10 |
| 1,3 | 3 | 6 | *Salix alba* | *Salix* | BROW | 0-10 |
| 1,4 | 3 | 6 | *Salix alba* | *Salix* | BROW | 0-10 |
| 1,5 | 3 | 6 | *Salix alba* | *Salix* | BROW | 0-10 |
| 1,5 | 3 | 6 | *Salix alba* | *Salix* | BROW | 0-10 |
| 1,3 | 3 | 6 | *Salix alba* | *Salix* | BROW | 0-10 |
| 1,3 | 3 | 6 | *Salix alba* | *Salix* | BROW | 0-10 |
| 1,3 | 3 | 6 | *Salix alba* | *Salix* | BROW | 0-10 |
| 1,3 | 3 | 6 | *Salix alba* | *Salix* | BROW | 0-10 |
| 1,2 | 3 | 6 | *Salix alba* | *Salix* | BROW | 0-10 |
| 1,2 | 3 | 6 | *Salix alba* | *Salix* | BROW | 0-10 |
| 1,5 | 3 | 6 | *Salix alba* | *Salix* | BROW | 0-10 |
| 1,5 | 3 | 6 | *Salix alba* | *Salix* | BROW | 0-10 |
| 1,5 | 3 | 6 | *Salix alba* | *Salix* | BROW | 0-10 |
| 1,5 | 3 | 6 | *Salix alba* | *Salix* | BROW | 0-10 |
| 1,5 | 3 | 6 | *Salix alba* | *Salix* | BROW | 0-10 |
| 1,5 | 3 | 6 | *Salix alba* | *Salix* | BROW | 0-10 |
| 1,6 | 3 | 6 | *Salix alba* | *Salix* | BROW | 0-10 |
| 1,7 | 3 | 6 | *Salix alba* | *Salix* | BROW | 0-10 |
| 1,4 | 3 | 6 | *Salix alba* | *Salix* | BROW | 0-10 |
| 1,4 | 3 | 6 | *Salix alba* | *Salix* | BROW | 0-10 |
| 1,4 | 3 | 6 | *Salix alba* | *Salix* | BROW | 0-10 |
| 1,4 | 3 | 6 | *Salix alba* | *Salix* | BROW | 0-10 |
| 1,4 | 3 | 6 | *Salix alba* | *Salix* | BROW | 0-10 |
| 1,4 | 3 | 6 | *Salix alba* | *Salix* | BROW | 0-10 |
| 1,4 | 3 | 6 | *Salix alba* | *Salix* | BROW | 0-10 |
| 1,4 | 3 | 6 | *Salix alba* | *Salix* | BROW | 0-10 |
| 1,4 | 3 | 6 | *Salix alba* | *Salix* | BROW | 0-10 |
| 1,4 | 3 | 6 | *Salix alba* | *Salix* | BROW | 0-10 |
| 1,4 | 3 | 6 | *Salix alba* | *Salix* | BROW | 0-10 |
| 1,4 | 3 | 6 | *Salix alba* | *Salix* | BROW | 0-10 |
| 1,4 | 3 | 6 | *Salix alba* | *Salix* | BROW | 0-10 |
| 1,4 | 3 | 6 | *Salix alba* | *Salix* | BROW | 0-10 |
| 1,4 | 3 | 6 | *Salix alba* | *Salix* | BROW | 0-10 |
| 1,4 | 3 | 6 | *Salix alba* | *Salix* | BROW | 0-10 |
| 1,4 | 3 | 6 | *Salix alba* | *Salix* | BROW | 0-10 |
| 1,4 | 3 | 6 | *Salix alba* | *Salix* | BROW | 0-10 |
| 1,4 | 3 | 6 | *Salix alba* | *Salix* | BROW | 0-10 |
| 1,4 | 3 | 6 | *Salix alba* | *Salix* | BROW | 0-10 |
| 1,4 | 3 | 6 | *Salix alba* | *Salix* | BROW | 0-10 |
| 1,4 | 3 | 6 | *Salix alba* | *Salix* | BROW | 0-10 |
| 1,5 | 3 | 6 | *Salix alba* | *Salix* | BROW | 0-10 |
| 1,5 | 3 | 6 | *Salix alba* | *Salix* | BROW | 0-10 |
| 1,5 | 3 | 6 | *Salix alba* | *Salix* | BROW | 0-10 |
| 1,5 | 3 | 6 | *Acer platanoides* | *Acer* | BROW | 0-10 |
| 1,6 | 3 | 6 | *Acer platanoides* | *Acer* | BROW | 0-10 |
| 1,4 | 3 | 6 | *Acer platanoides* | *Acer* | BROW | 0-10 |
| 1,7 | 3 | 6 | *Acer platanoides* | *Acer* | BROW | 0-10 |
| 1,6 | 3 | 6 | *Acer platanoides* | *Acer* | BROW | 0-10 |
| 1,2 | 3 | 6 | *Acer platanoides* | *Acer* | BROW | 0-10 |
| 1,3 | 3 | 6 | *Acer platanoides* | *Acer* | BROW | 0-10 |
| 1,4 | 3 | 6 | *Acer platanoides* | *Acer* | BROW | 0-10 |
| 1,4 | 3 | 6 | *Acer platanoides* | *Acer* | BROW | 0-10 |
| 1,5 | 3 | 6 | *Acer platanoides* | *Acer* | BROW | 0-10 |
| 1,6 | 3 | 6 | *Acer platanoides* | *Acer* | BROW | 0-10 |
| 1,4 | 3 | 6 | *Acer platanoides* | *Acer* | BROW | 0-10 |
| 1,7 | 3 | 6 | *Acer platanoides* | *Acer* | BROW | 0-10 |
| 1,6 | 3 | 6 | *Acer platanoides* | *Acer* | BROW | 0-10 |
| 1,2 | 3 | 6 | *Acer platanoides* | *Acer* | BROW | 0-10 |
| 1,3 | 3 | 6 | *Acer platanoides* | *Acer* | BROW | 0-10 |
| 1,4 | 3 | 6 | *Acer platanoides* | *Acer* | BROW | 0-10 |
| 1,4 | 3 | 6 | *Acer platanoides* | *Acer* | BROW | 0-10 |
| 1,5 | 3 | 6 | *Acer platanoides* | *Acer* | BROW | 0-10 |
| 1,6 | 3 | 6 | *Acer platanoides* | *Acer* | BROW | 0-10 |
| 1,4 | 3 | 6 | *Acer platanoides* | *Acer* | BROW | 0-10 |
| 1,7 | 3 | 6 | *Acer platanoides* | *Acer* | BROW | 0-10 |
| 1,6 | 3 | 6 | *Acer platanoides* | *Acer* | BROW | 0-10 |
| 1,2 | 3 | 6 | *Acer platanoides* | *Acer* | BROW | 0-10 |
| 1,3 | 3 | 6 | *Acer platanoides* | *Acer* | BROW | 0-10 |
| 1,4 | 3 | 6 | *Acer platanoides* | *Acer* | BROW | 0-10 |
| 1,4 | 3 | 6 | *Acer platanoides* | *Acer* | BROW | 0-10 |
| 1,5 | 3 | 6 | *Acer platanoides* | *Acer* | BROW | 0-10 |
| 1,5 | 3 | 6 | *Acer platanoides* | *Acer* | BROW | 0-10 |
| 1,3 | 3 | 6 | *Acer platanoides* | *Acer* | BROW | 0-10 |
| 1,3 | 3 | 6 | *Acer platanoides* | *Acer* | BROW | 0-10 |
| 1,7 | 3 | 6 | *Acer platanoides* | *Acer* | BROW | 0-10 |
| 1,7 | 3 | 6 | *Acer platanoides* | *Acer* | BROW | 0-10 |
| 1,6 | 3 | 6 | *Acer platanoides* | *Acer* | BROW | 0-10 |
| 1,4 | 3 | 6 | *Acer platanoides* | *Acer* | BROW | 0-10 |
| 1,5 | 3 | 6 | *Acer platanoides* | *Acer* | BROW | 0-10 |
| 1,6 | 3 | 6 | *Acer platanoides* | *Acer* | BROW | 0-10 |
| 1,4 | 3 | 6 | *Acer platanoides* | *Acer* | BROW | 0-10 |
| 1,7 | 3 | 6 | *Acer platanoides* | *Acer* | BROW | 0-10 |
| 1,6 | 3 | 6 | *Acer platanoides* | *Acer* | BROW | 0-10 |
| 1,2 | 3 | 6 | *Acer platanoides* | *Acer* | BROW | 0-10 |
| 1,3 | 3 | 6 | *Acer platanoides* | *Acer* | BROW | 0-10 |
| 1,4 | 3 | 6 | *Acer platanoides* | *Acer* | BROW | 0-10 |
| 1,4 | 3 | 6 | *Acer platanoides* | *Acer* | BROW | 0-10 |
| 1,5 | 3 | 6 | *Acer platanoides* | *Acer* | BROW | 0-10 |
| 1,6 | 3 | 6 | *Acer platanoides* | *Acer* | BROW | 0-10 |
| 1,4 | 3 | 6 | *Acer platanoides* | *Acer* | BROW | 0-10 |
| 1,7 | 3 | 6 | *Acer platanoides* | *Acer* | BROW | 0-10 |
| 1,6 | 3 | 6 | *Acer platanoides* | *Acer* | BROW | 0-10 |
| 1,2 | 3 | 6 | *Acer platanoides* | *Acer* | BROW | 0-10 |
| 1,3 | 3 | 6 | *Acer platanoides* | *Acer* | BROW | 0-10 |
| 1,4 | 3 | 6 | *Acer platanoides* | *Acer* | BROW | 0-10 |
| 1,4 | 3 | 6 | *Acer platanoides* | *Acer* | BROW | 0-10 |
| 1,5 | 3 | 6 | *Acer platanoides* | *Acer* | BROW | 0-10 |
| 1,6 | 3 | 6 | *Acer platanoides* | *Acer* | BROW | 0-10 |
| 1,4 | 3 | 6 | *Acer platanoides* | *Acer* | BROW | 0-10 |
| 1,7 | 3 | 6 | *Acer platanoides* | *Acer* | BROW | 0-10 |
| 1,6 | 3 | 6 | *Acer platanoides* | *Acer* | BROW | 0-10 |
| 1,2 | 3 | 6 | *Acer platanoides* | *Acer* | BROW | 0-10 |
| 1,3 | 3 | 6 | *Acer platanoides* | *Acer* | BROW | 0-10 |
| 1,4 | 3 | 6 | *Acer platanoides* | *Acer* | BROW | 0-10 |
| 1,4 | 3 | 6 | *Acer platanoides* | *Acer* | BROW | 0-10 |
| 1,5 | 3 | 6 | *Acer platanoides* | *Acer* | BROW | 0-10 |
| 1,5 | 3 | 6 | *Acer platanoides* | *Acer* | BROW | 0-10 |
| 1,3 | 3 | 6 | *Acer platanoides* | *Acer* | BROW | 0-10 |
| 1,3 | 3 | 6 | *Acer platanoides* | *Acer* | BROW | 0-10 |
| 1,7 | 3 | 6 | *Acer platanoides* | *Acer* | BROW | 0-10 |
| 1,7 | 3 | 6 | *Acer platanoides* | *Acer* | BROW | 0-10 |
| 1,6 | 3 | 6 | *Acer platanoides* | *Acer* | BROW | 0-10 |
| 1,4 | 3 | 6 | *Acer platanoides* | *Acer* | BROW | 0-10 |
| 1,5 | 3 | 6 | *Acer platanoides* | *Acer* | BROW | 0-10 |
| 1,5 | 3 | 6 | *Acer platanoides* | *Acer* | BROW | 0-10 |
| 1,5 | 3 | 6 | *Acer platanoides* | *Acer* | BROW | 0-10 |
| 1,5 | 3 | 6 | *Acer platanoides* | *Acer* | BROW | 0-10 |
| 1,5 | 3 | 6 | *Acer platanoides* | *Acer* | BROW | 0-10 |
| 1,5 | 3 | 6 | *Acer platanoides* | *Acer* | BROW | 0-10 |
| 1,5 | 3 | 6 | *Acer platanoides* | *Acer* | BROW | 0-10 |
| 1,5 | 3 | 6 | *Acer platanoides* | *Acer* | BROW | 0-10 |
| 1,5 | 3 | 6 | *Acer platanoides* | *Acer* | BROW | 0-10 |
| 1,5 | 3 | 6 | *Acer platanoides* | *Acer* | BROW | 0-10 |
| 1,5 | 3 | 6 | *Acer platanoides* | *Acer* | BROW | 0-10 |
| 1,5 | 3 | 6 | *Acer platanoides* | *Acer* | BROW | 0-10 |
| 1,5 | 3 | 6 | *Acer platanoides* | *Acer* | BROW | 0-10 |
| 1,5 | 3 | 6 | *Acer platanoides* | *Acer* | BROW | 0-10 |
| 1,5 | 3 | 6 | *Acer platanoides* | *Acer* | BROW | 0-10 |
| 1,5 | 3 | 6 | *Acer platanoides* | *Acer* | BROW | 0-10 |
| 1,5 | 3 | 6 | *Acer platanoides* | *Acer* | BROW | 0-10 |
| 1,5 | 3 | 6 | *Acer platanoides* | *Acer* | BROW | 0-10 |
| 1,5 | 3 | 6 | *Acer platanoides* | *Acer* | BROW | 0-10 |
| 1,5 | 3 | 6 | *Acer platanoides* | *Acer* | BROW | 0-10 |
| 1,5 | 3 | 6 | *Acer platanoides* | *Acer* | BROW | 0-10 |
| 1,5 | 3 | 6 | *Acer platanoides* | *Acer* | BROW | 0-10 |
| 1,5 | 3 | 6 | *Acer platanoides* | *Acer* | BROW | 0-10 |
| 1,5 | 3 | 6 | *Acer platanoides* | *Acer* | BROW | 0-10 |
| 1,5 | 3 | 6 | *Acer platanoides* | *Acer* | BROW | 0-10 |
| 1,6 | 3 | 6 | *Acer platanoides* | *Acer* | BROW | 0-10 |
| 1,7 | 3 | 6 | *Acer platanoides* | *Acer* | BROW | 0-10 |
| 1,3 | 3 | 6 | *Acer platanoides* | *Acer* | BROW | 0-10 |
| 1,4 | 3 | 6 | *Acer platanoides* | *Acer* | BROW | 0-10 |
| 1,4 | 3 | 6 | *Acer platanoides* | *Acer* | BROW | 0-10 |
| 1,5 | 3 | 6 | *Acer platanoides* | *Acer* | BROW | 0-10 |
| 1,4 | 3 | 6 | *Acer platanoides* | *Acer* | BROW | 0-10 |
| 1,3 | 3 | 6 | *Acer platanoides* | *Acer* | BROW | 0-10 |
| 1,5 | 3 | 6 | *Acer platanoides* | *Acer* | BROW | 0-10 |
| 1,6 | 3 | 6 | *Acer platanoides* | *Acer* | BROW | 0-10 |
| 1,5 | 3 | 6 | *Acer platanoides* | *Acer* | BROW | 0-10 |
| 1,4 | 3 | 6 | *Acer platanoides* | *Acer* | BROW | 0-10 |
| 1,4 | 3 | 6 | *Acer platanoides* | *Acer* | BROW | 0-10 |
| 1,4 | 3 | 6 | *Acer platanoides* | *Acer* | BROW | 0-10 |
| 1,6 | 3 | 6 | *Acer platanoides* | *Acer* | BROW | 0-10 |
| 1,6 | 3 | 6 | *Acer platanoides* | *Acer* | BROW | 0-10 |
| 1,8 | 3 | 6 | *Acer platanoides* | *Acer* | BROW | 0-10 |
| 1,8 | 3 | 6 | *Acer platanoides* | *Acer* | BROW | 0-10 |
| 1,5 | 3 | 6 | *Acer platanoides* | *Acer* | BROW | 0-10 |
| 1,5 | 3 | 6 | *Alnus glutinosa* | *Alnus* | BROW | 0-10 |
| 1,4 | 3 | 6 | *Alnus glutinosa* | *Alnus* | BROW | 0-10 |
| 1,3 | 3 | 6 | *Alnus glutinosa* | *Alnus* | BROW | 0-10 |
| 1,2 | 3 | 6 | *Alnus glutinosa* | *Alnus* | BROW | 0-10 |
| 1 | 3 | 6 | *Alnus glutinosa* | *Alnus* | BROW | 0-10 |
| 1,8 | 3 | 6 | *Alnus glutinosa* | *Alnus* | BROW | 0-10 |
| 1,9 | 3 | 6 | *Alnus glutinosa* | *Alnus* | BROW | 0-10 |
| 1,7 | 3 | 6 | *Alnus glutinosa* | *Alnus* | BROW | 0-10 |
| 1,7 | 3 | 6 | *Alnus glutinosa* | *Alnus* | BROW | 0-10 |
| 1,8 | 3 | 6 | *Alnus glutinosa* | *Alnus* | BROW | 0-10 |
| 1,5 | 3 | 6 | *Alnus glutinosa* | *Alnus* | BROW | 0-10 |
| 1,2 | 3 | 6 | *Alnus glutinosa* | *Alnus* | BROW | 0-10 |
| 1,3 | 3 | 6 | *Alnus glutinosa* | *Alnus* | BROW | 0-10 |
| 1,5 | 3 | 6 | *Alnus glutinosa* | *Alnus* | BROW | 0-10 |
| 1,6 | 3 | 6 | *Alnus glutinosa* | *Alnus* | BROW | 0-10 |
| 1,5 | 3 | 6 | *Alnus glutinosa* | *Alnus* | BROW | 0-10 |
| 1,5 | 3 | 6 | *Alnus glutinosa* | *Alnus* | BROW | 0-10 |
| 1,5 | 3 | 6 | *Alnus glutinosa* | *Alnus* | BROW | 0-10 |
| 1,5 | 3 | 6 | *Sambucus nigra* | *Sambucus* | BROW | 0-10 |
| 1,5 | 3 | 6 | *Sambucus nigra* | *Sambucus* | BROW | 0-10 |
| 1,5 | 3 | 6 | *Sambucus nigra* | *Sambucus* | BROW | 0-10 |
| 1,5 | 3 | 6 | *Sambucus nigra* | *Sambucus* | BROW | 0-10 |
| 1,3 | 3 | 6 | *Sambucus nigra* | *Sambucus* | BROW | 0-10 |
| 1,4 | 3 | 6 | *Sambucus nigra* | *Sambucus* | BROW | 0-10 |
| 1,5 | 3 | 6 | *Sambucus nigra* | *Sambucus* | BROW | 0-10 |
| 1,5 | 3 | 6 | *Sambucus nigra* | *Sambucus* | BROW | 0-10 |
| 1,5 | 3 | 6 | *Sambucus nigra* | *Sambucus* | BROW | 0-10 |
| 1,5 | 3 | 6 | *Sambucus nigra* | *Sambucus* | BROW | 0-10 |
| 9,5 | 3 | 6 | *Sambucus nigra* | *Sambucus* | BROW | 0-10 |
| 10,5 | 3 | 6 | *Sambucus nigra* | *Sambucus* | BROW | 0-10 |
| 8,9 | 3 | 6 | *Sambucus nigra* | *Sambucus* | BROW | 0-10 |
| 11,3 | 3 | 6 | *Sambucus nigra* | *Sambucus* | BROW | 0-10 |
| 8,7 | 3 | 6 | *Sambucus nigra* | *Sambucus* | BROW | 0-10 |
| 11,1 | 3 | 6 | *Sambucus nigra* | *Sambucus* | BROW | 0-10 |
| 10,1 | 3 | 6 | *Sambucus nigra* | *Sambucus* | BROW | 0-10 |
| 10,1 | 3 | 6 | *Sambucus nigra* | *Sambucus* | BROW | 0-10 |
| 9,8 | 3 | 6 | *Sambucus nigra* | *Sambucus* | BROW | 0-10 |
| 9,9 | 3 | 6 | *Sambucus nigra* | *Sambucus* | BROW | 0-10 |
| 1,5 | 3 | 6 | *Acer platanoides* | *Acer* | BROW | 0-10 |
| 1,5 | 3 | 6 | *Acer platanoides* | *Acer* | BROW | 0-10 |
| 1,4 | 3 | 6 | *Acer platanoides* | *Acer* | BROW | 0-10 |
| 1,4 | 3 | 6 | *Acer platanoides* | *Acer* | BROW | 0-10 |
| 1,3 | 3 | 6 | *Acer platanoides* | *Acer* | BROW | 0-10 |
| 1,6 | 3 | 6 | *Acer platanoides* | *Acer* | BROW | 0-10 |
| 1,6 | 3 | 6 | *Acer platanoides* | *Acer* | BROW | 0-10 |
| 1,7 | 3 | 6 | *Acer platanoides* | *Acer* | BROW | 0-10 |
| 1,7 | 3 | 6 | *Acer platanoides* | *Acer* | BROW | 0-10 |
| 1,5 | 3 | 6 | *Acer platanoides* | *Acer* | BROW | 0-10 |
| 43,8 | 4 | 9 | *Alnus glutinosa* | *Alnus* | UNBROW | 0-10 |
| 44,5 | 4 | 9 | *Alnus glutinosa* | *Alnus* | UNBROW | 0-10 |
| 3,8 | 4 | 9 | *Alnus glutinosa* | *Alnus* | BROW | 0-10 |
| 4,5 | 4 | 9 | *Alnus glutinosa* | *Alnus* | BROW | 0-10 |
| 7,8 | 4 | 9 | *Sambucus nigra* | *Sambucus* | UNBROW | 0-10 |
| 44,7 | 4 | 9 | *Alnus glutinosa* | *Alnus* | UNBROW | 0-10 |
| 36,6 | 4 | 9 | *Alnus glutinosa* | *Alnus* | UNBROW | 0-10 |
| 38,1 | 4 | 9 | *Alnus glutinosa* | *Alnus* | UNBROW | 0-10 |
| 40,4 | 4 | 9 | *Alnus glutinosa* | *Alnus* | UNBROW | 0-10 |
| 37,2 | 4 | 9 | *Betula pubescens* | Others | UNBROW | 0-10 |
| 41,1 | 4 | 9 | *Alnus glutinosa* | *Alnus* | UNBROW | 0-10 |
| 56,4 | 4 | 9 | *Populus alba* | *Populus* | BROW | 11-20 |
| 58,9 | 4 | 9 | *Populus alba* | *Populus* | UNBROW | 21-30 |
| 34,5 | 4 | 9 | *Populus alba* | *Populus* | UNBROW | 21-30 |
| 61,9 | 4 | 9 | *Populus alba* | *Populus* | UNBROW | 21-30 |
| 60,4 | 4 | 9 | *Populus alba* | *Populus* | UNBROW | 21-30 |
| 35,4 | 4 | 9 | *Betula pubescens* | Others | UNBROW | 21-30 |
| 39,4 | 4 | 9 | *Alnus glutinosa* | *Alnus* | UNBROW | 0-10 |
| 38,6 | 4 | 9 | *Alnus glutinosa* | *Alnus* | UNBROW | 0-10 |
| 3,2 | 4 | 9 | *Alnus glutinosa* | *Alnus* | BROW | 0-10 |
| 5,4 | 4 | 9 | *Alnus glutinosa* | *Alnus* | BROW | 0-10 |
| 49,1 | 4 | 9 | *Alnus glutinosa* | *Alnus* | UNBROW | 0-10 |
| 48 | 4 | 9 | *Alnus glutinosa* | *Alnus* | UNBROW | 0-10 |
| 5,7 | 4 | 9 | *Sambucus nigra* | *Sambucus* | UNBROW | 21-30 |
| 5,8 | 4 | 9 | *Sambucus nigra* | *Sambucus* | UNBROW | 21-30 |
| 3,5 | 4 | 9 | *Sambucus nigra* | *Sambucus* | UNBROW | 21-30 |
| 6,4 | 4 | 9 | *Sambucus nigra* | *Sambucus* | UNBROW | 21-30 |
| 3,1 | 4 | 9 | *Fraxinus excelsior* | Others | UNBROW | 0-10 |
| 2,3 | 4 | 9 | *Alnus glutinosa* | *Alnus* | BROW | 0-10 |
| 2,2 | 4 | 9 | *Alnus glutinosa* | *Alnus* | BROW | 0-10 |
| 4,2 | 4 | 9 | *Alnus glutinosa* | *Alnus* | BROW | 0-10 |
| 4 | 4 | 9 | *Alnus glutinosa* | *Alnus* | BROW | 0-10 |
| 9,9 | 4 | 9 | *Betula pubescens* | Others | BROW | 0-10 |
| 5,6 | 4 | 9 | *Sambucus nigra* | *Sambucus* | BROW | 11-20 |
| 6,4 | 4 | 9 | *Sambucus nigra* | *Sambucus* | UNBROW | 11-20 |
| 3,7 | 4 | 9 | *Sambucus nigra* | *Sambucus* | UNBROW | 11-20 |
| 10,4 | 4 | 9 | *Picea abies* | Others | BROW | 11-20 |
| 10,5 | 4 | 9 | *Picea abies* | Others | BROW | 11-20 |
| 13,2 | 4 | 9 | *Picea abies* | Others | UNBROW | 11-20 |
| 80 | 4 | 9 | *Populus nigra* | *Populus* | UNBROW | 11-20 |
| 80 | 4 | 9 | *Populus nigra* | *Populus* | UNBROW | 11-20 |
| 34,6 | 4 | 9 | *Alnus glutinosa* | *Alnus* | UNBROW | 0-10 |
| 6,7 | 4 | 9 | *Alnus glutinosa* | *Alnus* | UNBROW | 0-10 |
| 7,8 | 4 | 9 | *Alnus glutinosa* | *Alnus* | UNBROW | 0-10 |
| 5,7 | 4 | 9 | *Alnus glutinosa* | *Alnus* | UNBROW | 0-10 |
| 4,6 | 4 | 9 | *Alnus glutinosa* | *Alnus* | UNBROW | 0-10 |
| 4,9 | 4 | 9 | *Alnus glutinosa* | *Alnus* | UNBROW | 0-10 |
| 34,9 | 4 | 9 | *Alnus glutinosa* | *Alnus* | UNBROW | 0-10 |
| 5,1 | 4 | 9 | *Alnus glutinosa* | *Alnus* | UNBROW | 0-10 |
| 5 | 4 | 9 | *Alnus glutinosa* | *Alnus* | UNBROW | 0-10 |
| 2,1 | 4 | 9 | *Alnus glutinosa* | *Alnus* | UNBROW | 0-10 |
| 3,4 | 4 | 9 | *Sambucus nigra* | *Sambucus* | UNBROW | 0-10 |
| 3,7 | 4 | 9 | *Sambucus nigra* | *Sambucus* | UNBROW | 0-10 |
| 3,9 | 4 | 9 | *Sambucus nigra* | *Sambucus* | UNBROW | 0-10 |
| 5,6 | 4 | 9 | *Sambucus nigra* | *Sambucus* | UNBROW | 11-20 |
| 5,5 | 4 | 9 | *Sambucus nigra* | *Sambucus* | UNBROW | 11-20 |
| 5,4 | 4 | 9 | *Sambucus nigra* | *Sambucus* | UNBROW | 11-20 |
| 3,4 | 4 | 9 | *Sambucus nigra* | *Sambucus* | UNBROW | 11-20 |
| 7,8 | 4 | 9 | *Sambucus nigra* | *Sambucus* | UNBROW | 11-20 |
| 8,9 | 4 | 9 | *Sambucus nigra* | *Sambucus* | UNBROW | 11-20 |
| 8,1 | 4 | 9 | *Sambucus nigra* | *Sambucus* | UNBROW | 11-20 |
| 6,8 | 4 | 9 | *Sambucus nigra* | *Sambucus* | UNBROW | 11-20 |
| 7,3 | 4 | 9 | *Sambucus nigra* | *Sambucus* | UNBROW | 11-20 |
| 3,4 | 4 | 9 | *Sambucus nigra* | *Sambucus* | UNBROW | 11-20 |
| 2,8 | 4 | 9 | *Fraxinus excelsior* | Others | UNBROW | 0-10 |
| 45,5 | 4 | 9 | *Alnus glutinosa* | *Alnus* | UNBROW | 0-10 |
| 43,4 | 4 | 9 | *Alnus glutinosa* | *Alnus* | UNBROW | 0-10 |
| 3,4 | 4 | 9 | *Alnus glutinosa* | *Alnus* | UNBROW | 0-10 |
| 4,4 | 4 | 9 | *Alnus glutinosa* | *Alnus* | UNBROW | 0-10 |
| 4,4 | 4 | 9 | *Sambucus nigra* | *Sambucus* | UNBROW | 0-10 |
| 4,9 | 4 | 9 | *Sambucus nigra* | *Sambucus* | UNBROW | 0-10 |
| 5,1 | 4 | 9 | *Alnus glutinosa* | *Alnus* | UNBROW | 0-10 |
| 5 | 4 | 9 | *Sambucus nigra* | *Sambucus* | UNBROW | 0-10 |
| 2,6 | 4 | 9 | *Sambucus nigra* | *Sambucus* | UNBROW | 0-10 |
| 3,7 | 4 | 9 | *Sambucus nigra* | *Sambucus* | UNBROW | 0-10 |
| 9,8 | 4 | 9 | *Alnus glutinosa* | *Alnus* | BROW | 0-10 |
| 2,4 | 4 | 9 | *Alnus glutinosa* | *Alnus* | BROW | 0-10 |
| 80 | 4 | 9 | *Populus nigra* | *Populus* | UNBROW | 11-20 |
| 80 | 4 | 9 | *Populus nigra* | *Populus* | UNBROW | 11-20 |
| 3,5 | 4 | 9 | *Acer negundo* | *Acer* | BROW | 0-10 |
| 4,1 | 4 | 9 | *Acer negundo* | *Acer* | BROW | 0-10 |
| 23,4 | 4 | 9 | *Acer platanoides* | *Acer* | BROW | 0-10 |
| 2 | 4 | 9 | *Acer negundo* | *Acer* | BROW | 0-10 |
| 1,6 | 4 | 9 | *Acer negundo* | *Acer* | BROW | 0-10 |
| 1,9 | 4 | 9 | *Acer negundo* | *Acer* | BROW | 0-10 |
| 1,5 | 4 | 9 | *Acer negundo* | *Acer* | BROW | 0-10 |
| 1,5 | 4 | 9 | *Acer negundo* | *Acer* | BROW | 0-10 |
| 1,5 | 4 | 9 | *Acer negundo* | *Acer* | BROW | 0-10 |
| 1,5 | 4 | 9 | *Acer negundo* | *Acer* | BROW | 0-10 |
| 1,5 | 4 | 9 | *Acer negundo* | *Acer* | BROW | 0-10 |
| 1,4 | 4 | 9 | *Acer negundo* | *Acer* | BROW | 0-10 |
| 1,4 | 4 | 9 | *Acer negundo* | *Acer* | BROW | 0-10 |
| 1,3 | 4 | 9 | *Acer negundo* | *Acer* | BROW | 0-10 |
| 1,3 | 4 | 9 | *Acer negundo* | *Acer* | BROW | 0-10 |
| 1,3 | 4 | 9 | *Acer negundo* | *Acer* | BROW | 0-10 |
| 1,8 | 4 | 9 | *Acer negundo* | *Acer* | BROW | 0-10 |
| 1,8 | 4 | 9 | *Acer negundo* | *Acer* | BROW | 0-10 |
| 1,8 | 4 | 9 | *Acer negundo* | *Acer* | BROW | 0-10 |
| 1,9 | 4 | 9 | *Acer negundo* | *Acer* | BROW | 0-10 |
| 1,4 | 4 | 9 | *Acer negundo* | *Acer* | BROW | 0-10 |
| 1,6 | 4 | 9 | *Acer negundo* | *Acer* | BROW | 0-10 |
| 1,4 | 4 | 9 | *Acer negundo* | *Acer* | BROW | 0-10 |
| 1,8 | 4 | 9 | *Acer negundo* | *Acer* | BROW | 0-10 |
| 1,5 | 4 | 9 | *Acer negundo* | *Acer* | BROW | 0-10 |
| 0,9 | 4 | 9 | *Acer negundo* | *Acer* | BROW | 0-10 |
| 0,9 | 4 | 9 | *Acer negundo* | *Acer* | BROW | 0-10 |
| 1,1 | 4 | 9 | *Acer negundo* | *Acer* | BROW | 0-10 |
| 1,8 | 4 | 9 | *Acer negundo* | *Acer* | BROW | 0-10 |
| 1,9 | 4 | 9 | *Acer negundo* | *Acer* | BROW | 0-10 |
| 1,1 | 4 | 9 | *Acer negundo* | *Acer* | BROW | 0-10 |
| 1,2 | 4 | 9 | *Acer negundo* | *Acer* | BROW | 0-10 |
| 1,3 | 4 | 9 | *Acer negundo* | *Acer* | BROW | 0-10 |
| 1,4 | 4 | 9 | *Acer negundo* | *Acer* | BROW | 0-10 |
| 1,9 | 4 | 9 | *Acer negundo* | *Acer* | BROW | 0-10 |
| 1,9 | 4 | 9 | *Acer negundo* | *Acer* | BROW | 0-10 |
| 1,4 | 4 | 9 | *Acer negundo* | *Acer* | BROW | 0-10 |
| 1,3 | 4 | 9 | *Acer negundo* | *Acer* | BROW | 0-10 |
| 1,5 | 4 | 9 | *Acer negundo* | *Acer* | BROW | 0-10 |
| 1,5 | 4 | 9 | *Acer negundo* | *Acer* | BROW | 0-10 |
| 1,5 | 4 | 9 | *Acer negundo* | *Acer* | BROW | 0-10 |
| 1,5 | 4 | 9 | *Acer negundo* | *Acer* | BROW | 0-10 |
| 1,5 | 4 | 9 | *Acer negundo* | *Acer* | BROW | 0-10 |
| 1,5 | 4 | 9 | *Acer negundo* | *Acer* | BROW | 0-10 |
| 1,3 | 4 | 9 | *Acer negundo* | *Acer* | BROW | 0-10 |
| 1,7 | 4 | 9 | *Acer negundo* | *Acer* | BROW | 0-10 |
| 1,6 | 4 | 9 | *Acer negundo* | *Acer* | BROW | 0-10 |
| 1,2 | 4 | 9 | *Acer negundo* | *Acer* | BROW | 0-10 |
| 1,2 | 4 | 9 | *Acer negundo* | *Acer* | BROW | 0-10 |
| 1 | 4 | 9 | *Acer negundo* | *Acer* | BROW | 0-10 |
| 1,4 | 4 | 9 | *Acer negundo* | *Acer* | BROW | 0-10 |
| 1,4 | 4 | 9 | *Acer negundo* | *Acer* | BROW | 0-10 |
| 1,4 | 4 | 9 | *Acer negundo* | *Acer* | BROW | 0-10 |
| 1,6 | 4 | 9 | *Acer negundo* | *Acer* | BROW | 0-10 |
| 1,7 | 4 | 9 | *Acer negundo* | *Acer* | BROW | 0-10 |
| 1,9 | 4 | 9 | *Acer negundo* | *Acer* | BROW | 0-10 |
| 1,8 | 4 | 9 | *Acer negundo* | *Acer* | BROW | 0-10 |
| 1,6 | 4 | 9 | *Acer negundo* | *Acer* | BROW | 0-10 |
| 1,5 | 4 | 9 | *Acer negundo* | *Acer* | BROW | 0-10 |
| 1,5 | 4 | 9 | *Acer negundo* | *Acer* | BROW | 0-10 |
| 1,8 | 4 | 9 | *Acer negundo* | *Acer* | BROW | 0-10 |
| 1,8 | 4 | 9 | *Acer negundo* | *Acer* | BROW | 0-10 |
| 1,9 | 4 | 9 | *Acer negundo* | *Acer* | BROW | 0-10 |
| 1,5 | 4 | 9 | *Acer negundo* | *Acer* | BROW | 0-10 |
| 1,5 | 4 | 9 | *Acer negundo* | *Acer* | BROW | 0-10 |
| 1,9 | 4 | 9 | *Acer negundo* | *Acer* | BROW | 0-10 |
| 1,4 | 4 | 9 | *Acer negundo* | *Acer* | BROW | 0-10 |
| 1,3 | 4 | 9 | *Acer negundo* | *Acer* | BROW | 0-10 |
| 1,5 | 4 | 9 | *Acer negundo* | *Acer* | BROW | 0-10 |
| 1,5 | 4 | 9 | *Acer negundo* | *Acer* | BROW | 0-10 |
| 1,5 | 4 | 9 | *Acer negundo* | *Acer* | BROW | 0-10 |
| 1,5 | 4 | 9 | *Acer negundo* | *Acer* | BROW | 0-10 |
| 1,5 | 4 | 9 | *Acer negundo* | *Acer* | BROW | 0-10 |
| 1,5 | 4 | 9 | *Acer negundo* | *Acer* | BROW | 0-10 |
| 1,3 | 4 | 9 | *Acer negundo* | *Acer* | BROW | 0-10 |
| 1,7 | 4 | 9 | *Acer negundo* | *Acer* | BROW | 0-10 |
| 1,6 | 4 | 9 | *Acer negundo* | *Acer* | BROW | 0-10 |
| 1,2 | 4 | 9 | *Acer negundo* | *Acer* | BROW | 0-10 |
| 1,2 | 4 | 9 | *Acer negundo* | *Acer* | BROW | 0-10 |
| 1 | 4 | 9 | *Acer negundo* | *Acer* | BROW | 0-10 |
| 1,4 | 4 | 9 | *Acer negundo* | *Acer* | BROW | 0-10 |
| 1,4 | 4 | 9 | *Acer negundo* | *Acer* | BROW | 0-10 |
| 1,4 | 4 | 9 | *Acer negundo* | *Acer* | BROW | 0-10 |
| 1,6 | 4 | 9 | *Acer negundo* | *Acer* | BROW | 0-10 |
| 1,7 | 4 | 9 | *Acer negundo* | *Acer* | BROW | 0-10 |
| 1 | 4 | 9 | *Acer negundo* | *Acer* | BROW | 0-10 |
| 1 | 4 | 9 | *Acer negundo* | *Acer* | BROW | 0-10 |
| 1,1 | 4 | 9 | *Acer negundo* | *Acer* | BROW | 0-10 |
| 1,2 | 4 | 9 | *Acer negundo* | *Acer* | BROW | 0-10 |
| 1 | 4 | 9 | *Acer negundo* | *Acer* | BROW | 0-10 |
| 0,9 | 4 | 9 | *Acer negundo* | *Acer* | BROW | 0-10 |
| 1,8 | 4 | 9 | *Acer negundo* | *Acer* | BROW | 0-10 |
| 1,9 | 4 | 9 | *Acer negundo* | *Acer* | BROW | 0-10 |
| 1,5 | 4 | 9 | *Acer negundo* | *Acer* | BROW | 0-10 |
| 1,5 | 4 | 9 | *Acer negundo* | *Acer* | BROW | 0-10 |
| 1,5 | 4 | 9 | *Acer negundo* | *Acer* | BROW | 0-10 |
| 1,5 | 4 | 9 | *Acer negundo* | *Acer* | BROW | 0-10 |
| 1,6 | 4 | 9 | *Acer negundo* | *Acer* | BROW | 0-10 |
| 1,4 | 4 | 9 | *Acer negundo* | *Acer* | BROW | 0-10 |
| 1,4 | 4 | 9 | *Acer negundo* | *Acer* | BROW | 0-10 |
| 1,6 | 4 | 9 | *Acer negundo* | *Acer* | BROW | 0-10 |
| 1,7 | 4 | 9 | *Acer negundo* | *Acer* | BROW | 0-10 |
| 1,3 | 4 | 9 | *Acer negundo* | *Acer* | BROW | 0-10 |
| 1,3 | 4 | 9 | *Acer negundo* | *Acer* | BROW | 0-10 |
| 1,4 | 4 | 9 | *Acer negundo* | *Acer* | BROW | 0-10 |
| 1,9 | 4 | 9 | *Acer negundo* | *Acer* | BROW | 0-10 |
| 1,8 | 4 | 9 | *Acer negundo* | *Acer* | BROW | 0-10 |
| 1,5 | 4 | 9 | *Acer negundo* | *Acer* | BROW | 0-10 |
| 1,6 | 4 | 9 | *Acer negundo* | *Acer* | BROW | 0-10 |
| 1,5 | 4 | 9 | *Acer negundo* | *Acer* | BROW | 0-10 |
| 1,5 | 4 | 9 | *Acer negundo* | *Acer* | BROW | 0-10 |
| 1,4 | 4 | 9 | *Acer negundo* | *Acer* | BROW | 0-10 |
| 1,4 | 4 | 9 | *Acer negundo* | *Acer* | BROW | 0-10 |
| 1,6 | 4 | 9 | *Acer negundo* | *Acer* | BROW | 0-10 |
| 1,7 | 4 | 9 | *Acer negundo* | *Acer* | BROW | 0-10 |
| 1,5 | 4 | 9 | *Acer negundo* | *Acer* | BROW | 0-10 |
| 1,5 | 4 | 9 | *Acer negundo* | *Acer* | BROW | 0-10 |
| 1,5 | 4 | 9 | *Acer negundo* | *Acer* | BROW | 0-10 |
| 1,3 | 4 | 9 | *Acer negundo* | *Acer* | BROW | 0-10 |
| 1,3 | 4 | 9 | *Acer negundo* | *Acer* | BROW | 0-10 |
| 1,4 | 4 | 9 | *Acer negundo* | *Acer* | BROW | 0-10 |
| 1,9 | 4 | 9 | *Acer negundo* | *Acer* | BROW | 0-10 |
| 1,8 | 4 | 9 | *Acer negundo* | *Acer* | BROW | 0-10 |
| 1,5 | 4 | 9 | *Acer negundo* | *Acer* | BROW | 0-10 |
| 1,5 | 4 | 9 | *Acer negundo* | *Acer* | BROW | 0-10 |
| 1,2 | 4 | 9 | *Salix alba* | *Salix* | BROW | 0-10 |
| 1,2 | 4 | 9 | *Salix alba* | *Salix* | BROW | 0-10 |
| 1,4 | 4 | 9 | *Salix alba* | *Salix* | BROW | 0-10 |
| 1,7 | 4 | 9 | *Salix alba* | *Salix* | BROW | 0-10 |
| 1,8 | 4 | 9 | *Salix alba* | *Salix* | BROW | 0-10 |
| 1,9 | 4 | 9 | *Salix alba* | *Salix* | BROW | 0-10 |
| 1,5 | 4 | 9 | *Salix alba* | *Salix* | BROW | 0-10 |
| 1,5 | 4 | 9 | *Salix alba* | *Salix* | BROW | 0-10 |
| 1,6 | 4 | 9 | *Salix alba* | *Salix* | BROW | 0-10 |
| 1,7 | 4 | 9 | *Salix alba* | *Salix* | BROW | 0-10 |
| 1,4 | 4 | 9 | *Salix alba* | *Salix* | BROW | 0-10 |
| 1,3 | 4 | 9 | *Salix alba* | *Salix* | BROW | 0-10 |
| 1,5 | 4 | 9 | *Salix alba* | *Salix* | BROW | 0-10 |
| 1,6 | 4 | 9 | *Salix alba* | *Salix* | BROW | 0-10 |
| 1,4 | 4 | 9 | *Salix alba* | *Salix* | BROW | 0-10 |
| 1,7 | 4 | 9 | *Salix alba* | *Salix* | BROW | 0-10 |
| 1,7 | 4 | 9 | *Salix alba* | *Salix* | BROW | 0-10 |
| 1,8 | 4 | 9 | *Salix alba* | *Salix* | BROW | 0-10 |
| 1,8 | 4 | 9 | *Salix alba* | *Salix* | BROW | 0-10 |
| 1,1 | 4 | 9 | *Salix alba* | *Salix* | BROW | 0-10 |
| 1,2 | 4 | 9 | *Salix alba* | *Salix* | BROW | 0-10 |
| 1,3 | 4 | 9 | *Salix alba* | *Salix* | BROW | 0-10 |
| 1,4 | 4 | 9 | *Salix alba* | *Salix* | BROW | 0-10 |
| 1,3 | 4 | 9 | *Salix alba* | *Salix* | BROW | 0-10 |
| 1,4 | 4 | 9 | *Salix alba* | *Salix* | BROW | 0-10 |
| 1,5 | 4 | 9 | *Salix alba* | *Salix* | BROW | 0-10 |
| 1,5 | 4 | 9 | *Salix alba* | *Salix* | BROW | 0-10 |
| 1,5 | 4 | 9 | *Salix alba* | *Salix* | BROW | 0-10 |
| 1,3 | 4 | 9 | *Salix alba* | *Salix* | BROW | 0-10 |
| 1,3 | 4 | 9 | *Salix alba* | *Salix* | BROW | 0-10 |
| 1,6 | 4 | 9 | *Salix alba* | *Salix* | BROW | 0-10 |
| 1,7 | 4 | 9 | *Salix alba* | *Salix* | BROW | 0-10 |
| 1,7 | 4 | 9 | *Salix alba* | *Salix* | BROW | 0-10 |
| 1,7 | 4 | 9 | *Salix alba* | *Salix* | BROW | 0-10 |
| 1,8 | 4 | 9 | *Salix alba* | *Salix* | BROW | 0-10 |
| 1,7 | 4 | 9 | *Salix alba* | *Salix* | BROW | 0-10 |
| 1,9 | 4 | 9 | *Salix alba* | *Salix* | BROW | 0-10 |
| 1,6 | 4 | 9 | *Salix alba* | *Salix* | BROW | 0-10 |
| 1,5 | 4 | 9 | *Salix alba* | *Salix* | BROW | 0-10 |
| 1,2 | 4 | 9 | *Salix alba* | *Salix* | BROW | 0-10 |
| 1,3 | 4 | 9 | *Salix alba* | *Salix* | BROW | 0-10 |
| 1,6 | 4 | 9 | *Salix alba* | *Salix* | BROW | 0-10 |
| 1,7 | 4 | 9 | *Salix alba* | *Salix* | BROW | 0-10 |
| 1,8 | 4 | 9 | *Salix alba* | *Salix* | BROW | 0-10 |
| 1,9 | 4 | 9 | *Salix alba* | *Salix* | BROW | 0-10 |
| 1,4 | 4 | 9 | *Salix alba* | *Salix* | BROW | 0-10 |
| 1,4 | 4 | 9 | *Salix alba* | *Salix* | BROW | 0-10 |
| 1,4 | 4 | 9 | *Salix alba* | *Salix* | BROW | 0-10 |
| 1,6 | 4 | 9 | *Salix alba* | *Salix* | BROW | 0-10 |
| 1,6 | 4 | 9 | *Salix alba* | *Salix* | BROW | 0-10 |
| 1,8 | 4 | 9 | *Salix alba* | *Salix* | BROW | 0-10 |
| 1,5 | 4 | 9 | *Salix alba* | *Salix* | BROW | 0-10 |
| 1,5 | 4 | 9 | *Salix alba* | *Salix* | BROW | 0-10 |
| 1,4 | 4 | 9 | *Salix alba* | *Salix* | BROW | 0-10 |
| 1,6 | 4 | 9 | *Salix alba* | *Salix* | BROW | 0-10 |
| 1,9 | 4 | 9 | *Salix alba* | *Salix* | BROW | 0-10 |
| 1,8 | 4 | 9 | *Salix alba* | *Salix* | BROW | 0-10 |
| 1,7 | 4 | 9 | *Salix alba* | *Salix* | BROW | 0-10 |
| 1,5 | 4 | 9 | *Salix alba* | *Salix* | BROW | 0-10 |
| 1,2 | 4 | 9 | *Salix alba* | *Salix* | BROW | 0-10 |
| 1,3 | 4 | 9 | *Salix alba* | *Salix* | BROW | 0-10 |
| 1,2 | 4 | 9 | *Salix alba* | *Salix* | BROW | 0-10 |
| 1,3 | 4 | 9 | *Salix alba* | *Salix* | BROW | 0-10 |
| 1,4 | 4 | 9 | *Salix alba* | *Salix* | BROW | 0-10 |
| 1,3 | 4 | 9 | *Salix alba* | *Salix* | BROW | 0-10 |
| 1,4 | 4 | 9 | *Salix alba* | *Salix* | BROW | 0-10 |
| 1,5 | 4 | 9 | *Salix alba* | *Salix* | BROW | 0-10 |
| 1,5 | 4 | 9 | *Salix alba* | *Salix* | BROW | 0-10 |
| 1,7 | 4 | 9 | *Salix alba* | *Salix* | BROW | 0-10 |
| 1,7 | 4 | 9 | *Salix alba* | *Salix* | BROW | 0-10 |
| 1,8 | 4 | 9 | *Salix alba* | *Salix* | BROW | 0-10 |
| 1,7 | 4 | 9 | *Salix alba* | *Salix* | BROW | 0-10 |
| 1,9 | 4 | 9 | *Salix alba* | *Salix* | BROW | 0-10 |
| 1,6 | 4 | 9 | *Salix alba* | *Salix* | BROW | 0-10 |
| 1,5 | 4 | 9 | *Salix alba* | *Salix* | BROW | 0-10 |
| 1,2 | 4 | 9 | *Salix alba* | *Salix* | BROW | 0-10 |
| 1,3 | 4 | 9 | *Salix alba* | *Salix* | BROW | 0-10 |
| 1,6 | 4 | 9 | *Salix alba* | *Salix* | BROW | 0-10 |
| 1,7 | 4 | 9 | *Salix alba* | *Salix* | BROW | 0-10 |
| 1,8 | 4 | 9 | *Salix alba* | *Salix* | BROW | 0-10 |
| 1,9 | 4 | 9 | *Salix alba* | *Salix* | BROW | 0-10 |
| 1,4 | 4 | 9 | *Salix alba* | *Salix* | BROW | 0-10 |
| 1,4 | 4 | 9 | *Salix alba* | *Salix* | BROW | 0-10 |
| 1,4 | 4 | 9 | *Salix alba* | *Salix* | BROW | 0-10 |
| 1,6 | 4 | 9 | *Salix alba* | *Salix* | BROW | 0-10 |
| 1,6 | 4 | 9 | *Salix alba* | *Salix* | BROW | 0-10 |
| 1,8 | 4 | 9 | *Salix alba* | *Salix* | BROW | 0-10 |
| 1,5 | 4 | 9 | *Salix alba* | *Salix* | BROW | 0-10 |
| 1,5 | 4 | 9 | *Salix alba* | *Salix* | BROW | 0-10 |
| 1,4 | 4 | 9 | *Salix alba* | *Salix* | BROW | 0-10 |
| 1,6 | 4 | 9 | *Salix alba* | *Salix* | BROW | 0-10 |
| 1,9 | 4 | 9 | *Salix alba* | *Salix* | BROW | 0-10 |
| 1,8 | 4 | 9 | *Salix alba* | *Salix* | BROW | 0-10 |
| 1,7 | 4 | 9 | *Salix alba* | *Salix* | BROW | 0-10 |
| 1,5 | 4 | 9 | *Salix alba* | *Salix* | BROW | 0-10 |
| 1,2 | 4 | 9 | *Salix alba* | *Salix* | BROW | 0-10 |
| 1,3 | 4 | 9 | *Salix alba* | *Salix* | BROW | 0-10 |
| 1,2 | 4 | 9 | *Salix alba* | *Salix* | BROW | 0-10 |
| 1,3 | 4 | 9 | *Salix alba* | *Salix* | BROW | 0-10 |
| 1,4 | 4 | 9 | *Salix alba* | *Salix* | BROW | 0-10 |
| 1,3 | 4 | 9 | *Salix alba* | *Salix* | BROW | 0-10 |
| 1,4 | 4 | 9 | *Salix alba* | *Salix* | BROW | 0-10 |
| 1,5 | 4 | 9 | *Salix alba* | *Salix* | BROW | 0-10 |
| 1,5 | 4 | 9 | *Salix alba* | *Salix* | BROW | 0-10 |
| 1,3 | 4 | 9 | *Salix alba* | *Salix* | BROW | 0-10 |
| 1,3 | 4 | 9 | *Salix alba* | *Salix* | BROW | 0-10 |
| 1,3 | 4 | 9 | *Salix alba* | *Salix* | BROW | 0-10 |
| 1,3 | 4 | 9 | *Salix alba* | *Salix* | BROW | 0-10 |
| 1,2 | 4 | 9 | *Salix alba* | *Salix* | BROW | 0-10 |
| 1,2 | 4 | 9 | *Salix alba* | *Salix* | BROW | 0-10 |
| 1,5 | 4 | 9 | *Salix alba* | *Salix* | BROW | 0-10 |
| 1,5 | 4 | 9 | *Salix alba* | *Salix* | BROW | 0-10 |
| 1,5 | 4 | 9 | *Salix alba* | *Salix* | BROW | 0-10 |
| 1,5 | 4 | 9 | *Salix alba* | *Salix* | BROW | 0-10 |
| 1,5 | 4 | 9 | *Salix alba* | *Salix* | BROW | 0-10 |
| 1,5 | 4 | 9 | *Salix alba* | *Salix* | BROW | 0-10 |
| 1,6 | 4 | 9 | *Salix alba* | *Salix* | BROW | 0-10 |
| 1,7 | 4 | 9 | *Salix alba* | *Salix* | BROW | 0-10 |
| 1,7 | 4 | 9 | *Salix alba* | *Salix* | BROW | 0-10 |
| 1,6 | 4 | 9 | *Salix alba* | *Salix* | BROW | 0-10 |
| 1,5 | 4 | 9 | *Salix alba* | *Salix* | BROW | 0-10 |
| 1,2 | 4 | 9 | *Salix alba* | *Salix* | BROW | 0-10 |
| 1,2 | 4 | 9 | *Salix alba* | *Salix* | BROW | 0-10 |
| 1,3 | 4 | 9 | *Salix alba* | *Salix* | BROW | 0-10 |
| 1,3 | 4 | 9 | *Salix alba* | *Salix* | BROW | 0-10 |
| 1,2 | 4 | 9 | *Salix alba* | *Salix* | BROW | 0-10 |
| 1,3 | 4 | 9 | *Salix alba* | *Salix* | BROW | 0-10 |
| 1,3 | 4 | 9 | *Salix alba* | *Salix* | BROW | 0-10 |
| 1,3 | 4 | 9 | *Salix alba* | *Salix* | BROW | 0-10 |
| 1,3 | 4 | 9 | *Salix alba* | *Salix* | BROW | 0-10 |
| 1,3 | 4 | 9 | *Salix alba* | *Salix* | BROW | 0-10 |
| 1,4 | 4 | 9 | *Salix alba* | *Salix* | BROW | 0-10 |
| 1,4 | 4 | 9 | *Salix alba* | *Salix* | BROW | 0-10 |
| 1,4 | 4 | 9 | *Salix alba* | *Salix* | BROW | 0-10 |
| 1,4 | 4 | 9 | *Salix alba* | *Salix* | BROW | 0-10 |
| 1,4 | 4 | 9 | *Salix alba* | *Salix* | BROW | 0-10 |
| 1,4 | 4 | 9 | *Salix alba* | *Salix* | BROW | 0-10 |
| 1,4 | 4 | 9 | *Salix alba* | *Salix* | BROW | 0-10 |
| 1,5 | 4 | 9 | *Salix alba* | *Salix* | BROW | 0-10 |
| 1,5 | 4 | 9 | *Salix alba* | *Salix* | BROW | 0-10 |
| 1,5 | 4 | 9 | *Salix alba* | *Salix* | BROW | 0-10 |
| 1,5 | 4 | 9 | *Salix alba* | *Salix* | BROW | 0-10 |
| 1,5 | 4 | 9 | *Salix alba* | *Salix* | BROW | 0-10 |
| 1,5 | 4 | 9 | *Salix alba* | *Salix* | BROW | 0-10 |
| 1,5 | 4 | 9 | *Salix alba* | *Salix* | BROW | 0-10 |
| 1,5 | 4 | 9 | *Salix alba* | *Salix* | BROW | 0-10 |
| 1,5 | 4 | 9 | *Salix alba* | *Salix* | BROW | 0-10 |
| 1,5 | 4 | 9 | *Salix alba* | *Salix* | BROW | 0-10 |
| 1,5 | 4 | 9 | *Salix alba* | *Salix* | BROW | 0-10 |
| 1,5 | 4 | 9 | *Salix alba* | *Salix* | BROW | 0-10 |
| 1,4 | 4 | 9 | *Salix alba* | *Salix* | BROW | 0-10 |
| 1,6 | 4 | 9 | *Salix alba* | *Salix* | BROW | 0-10 |
| 1,9 | 4 | 9 | *Salix alba* | *Salix* | BROW | 0-10 |
| 1,8 | 4 | 9 | *Salix alba* | *Salix* | BROW | 0-10 |
| 1,7 | 4 | 9 | *Salix alba* | *Salix* | BROW | 0-10 |
| 1,5 | 4 | 9 | *Salix alba* | *Salix* | BROW | 0-10 |
| 1,2 | 4 | 9 | *Salix alba* | *Salix* | BROW | 0-10 |
| 1,3 | 4 | 9 | *Salix alba* | *Salix* | BROW | 0-10 |
| 1,2 | 4 | 9 | *Salix alba* | *Salix* | BROW | 0-10 |
| 1,3 | 4 | 9 | *Salix alba* | *Salix* | BROW | 0-10 |
| 1,4 | 4 | 9 | *Salix alba* | *Salix* | BROW | 0-10 |
| 1,3 | 4 | 9 | *Salix alba* | *Salix* | BROW | 0-10 |
| 1,4 | 4 | 9 | *Salix alba* | *Salix* | BROW | 0-10 |
| 1,5 | 4 | 9 | *Salix alba* | *Salix* | BROW | 0-10 |
| 1,5 | 4 | 9 | *Salix alba* | *Salix* | BROW | 0-10 |
| 1,3 | 4 | 9 | *Salix alba* | *Salix* | BROW | 0-10 |
| 1,3 | 4 | 9 | *Salix alba* | *Salix* | BROW | 0-10 |
| 1,3 | 4 | 9 | *Salix alba* | *Salix* | BROW | 0-10 |
| 1,3 | 4 | 9 | *Salix alba* | *Salix* | BROW | 0-10 |
| 1,2 | 4 | 9 | *Salix alba* | *Salix* | BROW | 0-10 |
| 1,2 | 4 | 9 | *Salix alba* | *Salix* | BROW | 0-10 |
| 1,5 | 4 | 9 | *Salix alba* | *Salix* | BROW | 0-10 |
| 1,5 | 4 | 9 | *Salix alba* | *Salix* | BROW | 0-10 |
| 1,5 | 4 | 9 | *Salix alba* | *Salix* | BROW | 0-10 |
| 1,5 | 4 | 9 | *Salix alba* | *Salix* | BROW | 0-10 |
| 1,5 | 4 | 9 | *Salix alba* | *Salix* | BROW | 0-10 |
| 1,5 | 4 | 9 | *Salix alba* | *Salix* | BROW | 0-10 |
| 1,6 | 4 | 9 | *Salix alba* | *Salix* | BROW | 0-10 |
| 1,7 | 4 | 9 | *Salix alba* | *Salix* | BROW | 0-10 |
| 1,7 | 4 | 9 | *Salix alba* | *Salix* | BROW | 0-10 |
| 1,6 | 4 | 9 | *Salix alba* | *Salix* | BROW | 0-10 |
| 1,5 | 4 | 9 | *Salix alba* | *Salix* | BROW | 0-10 |
| 1,2 | 4 | 9 | *Salix alba* | *Salix* | BROW | 0-10 |
| 1,2 | 4 | 9 | *Salix alba* | *Salix* | BROW | 0-10 |
| 1,3 | 4 | 9 | *Salix alba* | *Salix* | BROW | 0-10 |
| 1,3 | 4 | 9 | *Salix alba* | *Salix* | BROW | 0-10 |
| 1,2 | 4 | 9 | *Salix alba* | *Salix* | BROW | 0-10 |
| 1,3 | 4 | 9 | *Salix alba* | *Salix* | BROW | 0-10 |
| 1,3 | 4 | 9 | *Salix alba* | *Salix* | BROW | 0-10 |
| 1,3 | 4 | 9 | *Salix alba* | *Salix* | BROW | 0-10 |
| 1,3 | 4 | 9 | *Salix alba* | *Salix* | BROW | 0-10 |
| 1,3 | 4 | 9 | *Salix alba* | *Salix* | BROW | 0-10 |
| 1,4 | 4 | 9 | *Salix alba* | *Salix* | BROW | 0-10 |
| 1,4 | 4 | 9 | *Salix alba* | *Salix* | BROW | 0-10 |
| 1,4 | 4 | 9 | *Salix alba* | *Salix* | BROW | 0-10 |
| 1,4 | 4 | 9 | *Salix alba* | *Salix* | BROW | 0-10 |
| 1,4 | 4 | 9 | *Salix alba* | *Salix* | BROW | 0-10 |
| 1,4 | 4 | 9 | *Salix alba* | *Salix* | BROW | 0-10 |
| 1,4 | 4 | 9 | *Salix alba* | *Salix* | BROW | 0-10 |
| 1,5 | 4 | 9 | *Salix alba* | *Salix* | BROW | 0-10 |
| 1,5 | 4 | 9 | *Salix alba* | *Salix* | BROW | 0-10 |
| 1,5 | 4 | 9 | *Salix alba* | *Salix* | BROW | 0-10 |
| 1,5 | 4 | 9 | *Salix alba* | *Salix* | BROW | 0-10 |
| 1,4 | 4 | 9 | *Salix alba* | *Salix* | BROW | 0-10 |
| 1,6 | 4 | 9 | *Salix alba* | *Salix* | BROW | 0-10 |
| 1,4 | 4 | 9 | *Salix alba* | *Salix* | BROW | 0-10 |
| 1,4 | 4 | 9 | *Salix alba* | *Salix* | BROW | 0-10 |
| 1,5 | 4 | 9 | *Salix alba* | *Salix* | BROW | 0-10 |
| 1,5 | 4 | 9 | *Salix alba* | *Salix* | BROW | 0-10 |
| 1,5 | 4 | 9 | *Salix alba* | *Salix* | BROW | 0-10 |
| 1,5 | 4 | 9 | *Salix alba* | *Salix* | BROW | 0-10 |
| 1,4 | 4 | 9 | *Salix alba* | *Salix* | BROW | 0-10 |
| 1,6 | 4 | 9 | *Salix alba* | *Salix* | BROW | 0-10 |
| 1,5 | 4 | 9 | *Salix alba* | *Salix* | BROW | 0-10 |
| 1,5 | 4 | 9 | *Salix alba* | *Salix* | BROW | 0-10 |
| 1,5 | 4 | 9 | *Salix alba* | *Salix* | BROW | 0-10 |
| 1,5 | 4 | 9 | *Salix alba* | *Salix* | BROW | 0-10 |
| 1,5 | 4 | 9 | *Salix alba* | *Salix* | BROW | 0-10 |
| 1,5 | 4 | 9 | *Salix alba* | *Salix* | BROW | 0-10 |
| 1,4 | 4 | 9 | *Salix alba* | *Salix* | BROW | 0-10 |
| 1,4 | 4 | 9 | *Salix alba* | *Salix* | BROW | 0-10 |
| 1,5 | 4 | 9 | *Salix alba* | *Salix* | BROW | 0-10 |
| 1,5 | 4 | 9 | *Salix alba* | *Salix* | BROW | 0-10 |
| 1,5 | 4 | 9 | *Salix alba* | *Salix* | BROW | 0-10 |
| 1,5 | 4 | 9 | *Salix alba* | *Salix* | BROW | 0-10 |
| 1,4 | 4 | 9 | *Salix alba* | *Salix* | BROW | 0-10 |
| 1,6 | 4 | 9 | *Salix alba* | *Salix* | BROW | 0-10 |
| 1,5 | 4 | 9 | *Salix alba* | *Salix* | BROW | 0-10 |
| 1,5 | 4 | 9 | *Salix alba* | *Salix* | BROW | 0-10 |
| 1,5 | 4 | 9 | *Salix alba* | *Salix* | BROW | 0-10 |
| 1,5 | 4 | 9 | *Salix alba* | *Salix* | BROW | 0-10 |
| 1,5 | 4 | 9 | *Salix alba* | *Salix* | BROW | 0-10 |
| 1,5 | 4 | 9 | *Salix alba* | *Salix* | BROW | 0-10 |
| 5,5 | 5 | 10 | *Sambucus nigra* | *Sambucus* | UNBROW | 0-10 |
| 5,6 | 5 | 10 | *Sambucus nigra* | *Sambucus* | UNBROW | 0-10 |
| 5,9 | 5 | 10 | *Sambucus nigra* | *Sambucus* | UNBROW | 0-10 |
| 39,8 | 5 | 10 | *Alnus glutinosa* | *Alnus* | UNBROW | 0-10 |
| 40,2 | 5 | 10 | *Alnus glutinosa* | *Alnus* | UNBROW | 0-10 |
| 6,2 | 5 | 10 | *Sambucus nigra* | *Sambucus* | UNBROW | 0-10 |
| 7,5 | 5 | 10 | *Alnus glutinosa* | *Alnus* | UNBROW | 0-10 |
| 6,7 | 5 | 10 | *Alnus glutinosa* | *Alnus* | UNBROW | 0-10 |
| 6,4 | 5 | 10 | *Quercus rubra* | *Quercus* | BROW | 11-20 |
| 6,7 | 5 | 10 | *Quercus rubra* | *Quercus* | BROW | 11-20 |
| 6,6 | 5 | 10 | *Quercus rubra* | *Quercus* | BROW | 11-20 |
| 6,8 | 5 | 10 | *Quercus rubra* | *Quercus* | BROW | 11-20 |
| 6,3 | 5 | 10 | *Quercus rubra* | *Quercus* | BROW | 11-20 |
| 6,4 | 5 | 10 | *Quercus rubra* | *Quercus* | UNBROW | 11-20 |
| 6,7 | 5 | 10 | *Quercus rubra* | *Quercus* | UNBROW | 11-20 |
| 6,6 | 5 | 10 | *Quercus rubra* | *Quercus* | UNBROW | 11-20 |
| 6,5 | 5 | 10 | *Quercus rubra* | *Quercus* | UNBROW | 11-20 |
| 6,5 | 5 | 10 | *Quercus rubra* | *Quercus* | UNBROW | 11-20 |
| 37,5 | 5 | 10 | *Alnus glutinosa* | *Alnus* | UNBROW | 0-10 |
| 36,5 | 5 | 10 | *Alnus glutinosa* | *Alnus* | UNBROW | 0-10 |
| 3,4 | 5 | 10 | *Sambucus nigra* | *Sambucus* | BROW | 0-10 |
| 3,5 | 5 | 10 | *Alnus glutinosa* | *Alnus* | BROW | 0-10 |
| 3,2 | 5 | 10 | *Sambucus nigra* | *Sambucus* | UNBROW | 0-10 |
| 5,6 | 5 | 10 | *Malus sylvestris* | Others | BROW | 0-10 |
| 34,1 | 5 | 10 | *Alnus glutinosa* | *Alnus* | UNBROW | 0-10 |
| 9,8 | 5 | 10 | *Crataegus* sp. | *Crataegus* | BROW | 0-10 |
| 2,3 | 5 | 10 | *Alnus glutinosa* | *Alnus* | UNBROW | 0-10 |
| 2,1 | 5 | 10 | *Alnus glutinosa* | *Alnus* | UNBROW | 0-10 |
| 47,8 | 5 | 10 | *Alnus glutinosa* | *Alnus* | UNBROW | 0-10 |
| 45,6 | 5 | 10 | *Alnus glutinosa* | *Alnus* | UNBROW | 0-10 |
| 1,9 | 5 | 10 | *Salix alba* | *Salix* | UNBROW | 0-10 |
| 1,8 | 5 | 10 | *Salix alba* | *Salix* | UNBROW | 0-10 |
| 2 | 5 | 10 | *Salix alba* | *Salix* | UNBROW | 0-10 |
| 2 | 5 | 10 | *Salix alba* | *Salix* | UNBROW | 0-10 |
| 2,3 | 5 | 10 | *Salix alba* | *Salix* | UNBROW | 0-10 |
| 1,5 | 5 | 10 | *Salix alba* | *Salix* | UNBROW | 0-10 |
| 1,4 | 5 | 10 | *Salix alba* | *Salix* | UNBROW | 0-10 |
| 2,3 | 5 | 10 | *Acer negundo* | *Acer* | BROW | 0-10 |
| 2,2 | 5 | 10 | *Acer negundo* | *Acer* | BROW | 0-10 |
| 2,1 | 5 | 10 | *Acer negundo* | *Acer* | UNBROW | 0-10 |
| 78,9 | 5 | 10 | *Populus nigra* | *Populus* | UNBROW | 11-20 |
| 80 | 5 | 10 | *Populus nigra* | *Populus* | UNBROW | 11-20 |
| 3,4 | 5 | 10 | *Populus nigra* | *Populus* | UNBROW | 11-20 |
| 76,5 | 5 | 10 | *Populus nigra* | *Populus* | UNBROW | 11-20 |
| 34,4 | 5 | 10 | *Alnus glutinosa* | *Alnus* | UNBROW | 11-20 |
| 41,2 | 5 | 10 | *Alnus glutinosa* | *Alnus* | UNBROW | 11-20 |
| 42,3 | 5 | 10 | *Alnus glutinosa* | *Alnus* | UNBROW | 11-20 |
| 41,4 | 5 | 10 | *Alnus glutinosa* | *Alnus* | UNBROW | 11-20 |
| 45,5 | 5 | 10 | *Alnus glutinosa* | *Alnus* | BROW | 11-20 |
| 44,7 | 5 | 10 | *Alnus glutinosa* | *Alnus* | UNBROW | 21-30 |
| 7,8 | 5 | 10 | *Alnus glutinosa* | *Alnus* | UNBROW | 21-30 |
| 7,9 | 5 | 10 | *Alnus glutinosa* | *Alnus* | UNBROW | 21-30 |
| 3,4 | 5 | 10 | *Alnus glutinosa* | *Alnus* | UNBROW | 21-30 |
| 5,6 | 5 | 10 | *Alnus glutinosa* | *Alnus* | UNBROW | 21-30 |
| 3,2 | 5 | 10 | *Alnus glutinosa* | *Alnus* | UNBROW | 21-30 |
| 5,1 | 5 | 10 | *Alnus glutinosa* | *Alnus* | UNBROW | 21-30 |
| 4,4 | 5 | 10 | *Alnus glutinosa* | *Alnus* | UNBROW | 21-30 |
| 4,3 | 5 | 10 | *Alnus glutinosa* | *Alnus* | UNBROW | 21-30 |
| 3,1 | 5 | 10 | *Alnus glutinosa* | *Alnus* | UNBROW | 11-20 |
| 57,8 | 5 | 10 | *Populus alba* | *Populus* | UNBROW | 11-20 |
| 54,9 | 5 | 10 | *Populus alba* | *Populus* | UNBROW | 11-20 |
| 52,8 | 5 | 10 | *Populus alba* | *Populus* | UNBROW | 11-20 |
| 51,6 | 5 | 10 | *Salix alba* | *Salix* | UNBROW | 11-20 |
| 50,1 | 5 | 10 | *Salix alba* | *Salix* | UNBROW | 11-20 |
| 57,3 | 5 | 10 | *Salix sepulcralis* | *Salix* | UNBROW | 11-20 |
| 10,1 | 5 | 10 | *Acer platanoides* | *Acer* | BROW | 11-20 |
| 10,3 | 5 | 10 | *Acer platanoides* | *Acer* | BROW | 11-20 |
| 4,8 | 5 | 10 | *Acer negundo* | *Acer* | BROW | 0-10 |
| 33,9 | 5 | 10 | *Alnus glutinosa* | *Alnus* | UNBROW | 0-10 |
| 44,2 | 5 | 10 | *Alnus glutinosa* | *Alnus* | UNBROW | 0-10 |
| 2,9 | 5 | 10 | *Alnus glutinosa* | *Alnus* | UNBROW | 0-10 |
| 2,7 | 5 | 10 | *Sambucus nigra* | *Sambucus* | UNBROW | 0-10 |
| 32,3 | 5 | 10 | *Alnus glutinosa* | *Alnus* | UNBROW | 0-10 |
| 5,4 | 5 | 10 | *Sambucus nigra* | *Sambucus* | BROW | 0-10 |
| 5,7 | 5 | 10 | *Sambucus nigra* | *Sambucus* | BROW | 0-10 |
| 6,1 | 5 | 10 | *Sambucus nigra* | *Sambucus* | BROW | 0-10 |
| 7,3 | 5 | 10 | *Sambucus nigra* | *Sambucus* | BROW | 0-10 |
| 3,3 | 5 | 10 | *Sambucus nigra* | *Sambucus* | UNBROW | 0-10 |
| 1,5 | 5 | 10 | *Salix alba* | *Salix* | BROW | 0-10 |
| 1,5 | 5 | 10 | *Salix alba* | *Salix* | BROW | 0-10 |
| 1,5 | 5 | 10 | *Salix alba* | *Salix* | BROW | 0-10 |
| 1,2 | 5 | 10 | *Salix alba* | *Salix* | BROW | 0-10 |
| 1,2 | 5 | 10 | *Salix alba* | *Salix* | BROW | 0-10 |
| 1,4 | 5 | 10 | *Salix alba* | *Salix* | BROW | 0-10 |
| 1,7 | 5 | 10 | *Salix alba* | *Salix* | BROW | 0-10 |
| 1,8 | 5 | 10 | *Salix alba* | *Salix* | BROW | 0-10 |
| 1,9 | 5 | 10 | *Salix alba* | *Salix* | BROW | 0-10 |
| 1,5 | 5 | 10 | *Salix alba* | *Salix* | BROW | 0-10 |
| 1,5 | 5 | 10 | *Salix alba* | *Salix* | BROW | 0-10 |
| 1,6 | 5 | 10 | *Salix alba* | *Salix* | BROW | 0-10 |
| 1,7 | 5 | 10 | *Salix alba* | *Salix* | BROW | 0-10 |
| 1,4 | 5 | 10 | *Salix alba* | *Salix* | BROW | 0-10 |
| 1,3 | 5 | 10 | *Salix alba* | *Salix* | BROW | 0-10 |
| 1,5 | 5 | 10 | *Salix alba* | *Salix* | BROW | 0-10 |
| 1,6 | 5 | 10 | *Salix alba* | *Salix* | BROW | 0-10 |
| 1,4 | 5 | 10 | *Salix alba* | *Salix* | BROW | 0-10 |
| 1,7 | 5 | 10 | *Salix alba* | *Salix* | BROW | 0-10 |
| 1,7 | 5 | 10 | *Salix alba* | *Salix* | BROW | 0-10 |
| 1,8 | 5 | 10 | *Salix alba* | *Salix* | BROW | 0-10 |
| 1,8 | 5 | 10 | *Salix alba* | *Salix* | BROW | 0-10 |
| 1,1 | 5 | 10 | *Salix alba* | *Salix* | BROW | 0-10 |
| 1,2 | 5 | 10 | *Salix alba* | *Salix* | BROW | 0-10 |
| 1,3 | 5 | 10 | *Salix alba* | *Salix* | BROW | 0-10 |
| 1,4 | 5 | 10 | *Salix alba* | *Salix* | BROW | 0-10 |
| 1,3 | 5 | 10 | *Salix alba* | *Salix* | BROW | 0-10 |
| 1,4 | 5 | 10 | *Salix alba* | *Salix* | BROW | 0-10 |
| 1,5 | 5 | 10 | *Salix alba* | *Salix* | BROW | 0-10 |
| 1,5 | 5 | 10 | *Salix alba* | *Salix* | BROW | 0-10 |
| 1,5 | 5 | 10 | *Salix alba* | *Salix* | BROW | 0-10 |
| 1,3 | 5 | 10 | *Salix alba* | *Salix* | BROW | 0-10 |
| 1,3 | 5 | 10 | *Salix alba* | *Salix* | BROW | 0-10 |
| 1,6 | 5 | 10 | *Salix alba* | *Salix* | BROW | 0-10 |
| 1,7 | 5 | 10 | *Salix alba* | *Salix* | BROW | 0-10 |
| 1,7 | 5 | 10 | *Salix alba* | *Salix* | BROW | 0-10 |
| 1,7 | 5 | 10 | *Salix alba* | *Salix* | BROW | 0-10 |
| 1,8 | 5 | 10 | *Salix alba* | *Salix* | BROW | 0-10 |
| 1,7 | 5 | 10 | *Salix alba* | *Salix* | BROW | 0-10 |
| 1,9 | 5 | 10 | *Salix alba* | *Salix* | BROW | 0-10 |
| 1,6 | 5 | 10 | *Salix alba* | *Salix* | BROW | 0-10 |
| 1,5 | 5 | 10 | *Salix alba* | *Salix* | BROW | 0-10 |
| 1,2 | 5 | 10 | *Salix alba* | *Salix* | BROW | 0-10 |
| 1,3 | 5 | 10 | *Salix alba* | *Salix* | BROW | 0-10 |
| 1,6 | 5 | 10 | *Salix alba* | *Salix* | BROW | 0-10 |
| 1,7 | 5 | 10 | *Salix alba* | *Salix* | BROW | 0-10 |
| 1,8 | 5 | 10 | *Salix alba* | *Salix* | BROW | 0-10 |
| 1,9 | 5 | 10 | *Salix alba* | *Salix* | BROW | 0-10 |
| 1,4 | 5 | 10 | *Salix alba* | *Salix* | BROW | 0-10 |
| 1,4 | 5 | 10 | *Salix alba* | *Salix* | BROW | 0-10 |
| 1,4 | 5 | 10 | *Salix alba* | *Salix* | BROW | 0-10 |
| 1,6 | 5 | 10 | *Salix alba* | *Salix* | BROW | 0-10 |
| 1,6 | 5 | 10 | *Salix alba* | *Salix* | BROW | 0-10 |
| 1,8 | 5 | 10 | *Salix alba* | *Salix* | BROW | 0-10 |
| 1,5 | 5 | 10 | *Salix alba* | *Salix* | BROW | 0-10 |
| 1,5 | 5 | 10 | *Salix alba* | *Salix* | BROW | 0-10 |
| 1,4 | 5 | 10 | *Salix alba* | *Salix* | BROW | 0-10 |
| 1,6 | 5 | 10 | *Salix alba* | *Salix* | BROW | 0-10 |
| 1,9 | 5 | 10 | *Salix alba* | *Salix* | BROW | 0-10 |
| 1,8 | 5 | 10 | *Salix alba* | *Salix* | BROW | 0-10 |
| 1,7 | 5 | 10 | *Salix alba* | *Salix* | BROW | 0-10 |
| 1,5 | 5 | 10 | *Salix alba* | *Salix* | BROW | 0-10 |
| 1,2 | 5 | 10 | *Salix alba* | *Salix* | BROW | 0-10 |
| 1,3 | 5 | 10 | *Salix alba* | *Salix* | BROW | 0-10 |
| 1,2 | 5 | 10 | *Salix alba* | *Salix* | BROW | 0-10 |
| 1,5 | 5 | 10 | *Salix alba* | *Salix* | BROW | 0-10 |
| 1,2 | 5 | 10 | *Salix alba* | *Salix* | BROW | 0-10 |
| 1,2 | 5 | 10 | *Salix alba* | *Salix* | BROW | 0-10 |
| 1,4 | 5 | 10 | *Salix alba* | *Salix* | BROW | 0-10 |
| 1,7 | 5 | 10 | *Salix alba* | *Salix* | BROW | 0-10 |
| 1,8 | 5 | 10 | *Salix alba* | *Salix* | BROW | 0-10 |
| 1,9 | 5 | 10 | *Salix alba* | *Salix* | BROW | 0-10 |
| 1,5 | 5 | 10 | *Salix alba* | *Salix* | BROW | 0-10 |
| 1,5 | 5 | 10 | *Salix alba* | *Salix* | BROW | 0-10 |
| 1,6 | 5 | 10 | *Salix alba* | *Salix* | BROW | 0-10 |
| 1,7 | 5 | 10 | *Salix alba* | *Salix* | BROW | 0-10 |
| 1,4 | 5 | 10 | *Salix alba* | *Salix* | BROW | 0-10 |
| 1,3 | 5 | 10 | *Salix alba* | *Salix* | BROW | 0-10 |
| 1,5 | 5 | 10 | *Salix alba* | *Salix* | BROW | 0-10 |
| 1,6 | 5 | 10 | *Salix alba* | *Salix* | BROW | 0-10 |
| 1,4 | 5 | 10 | *Salix alba* | *Salix* | BROW | 0-10 |
| 1,7 | 5 | 10 | *Salix alba* | *Salix* | BROW | 0-10 |
| 1,7 | 5 | 10 | *Salix alba* | *Salix* | BROW | 0-10 |
| 1,8 | 5 | 10 | *Salix alba* | *Salix* | BROW | 0-10 |
| 1,8 | 5 | 10 | *Salix alba* | *Salix* | BROW | 0-10 |
| 1,1 | 5 | 10 | *Salix alba* | *Salix* | BROW | 0-10 |
| 1,2 | 5 | 10 | *Salix alba* | *Salix* | BROW | 0-10 |
| 1,3 | 5 | 10 | *Salix alba* | *Salix* | BROW | 0-10 |
| 1,4 | 5 | 10 | *Salix alba* | *Salix* | BROW | 0-10 |
| 1,3 | 5 | 10 | *Salix alba* | *Salix* | BROW | 0-10 |
| 1,4 | 5 | 10 | *Salix alba* | *Salix* | BROW | 0-10 |
| 1,5 | 5 | 10 | *Salix alba* | *Salix* | BROW | 0-10 |
| 1,5 | 5 | 10 | *Salix alba* | *Salix* | BROW | 0-10 |
| 1,5 | 5 | 10 | *Salix alba* | *Salix* | BROW | 0-10 |
| 1,3 | 5 | 10 | *Salix alba* | *Salix* | BROW | 0-10 |
| 1,3 | 5 | 10 | *Salix alba* | *Salix* | BROW | 0-10 |
| 1,6 | 5 | 10 | *Salix alba* | *Salix* | BROW | 0-10 |
| 1,7 | 5 | 10 | *Salix alba* | *Salix* | BROW | 0-10 |
| 1,7 | 5 | 10 | *Salix alba* | *Salix* | BROW | 0-10 |
| 1,7 | 5 | 10 | *Salix alba* | *Salix* | BROW | 0-10 |
| 1,8 | 5 | 10 | *Salix alba* | *Salix* | BROW | 0-10 |
| 1,7 | 5 | 10 | *Salix alba* | *Salix* | BROW | 0-10 |
| 1,9 | 5 | 10 | *Salix alba* | *Salix* | BROW | 0-10 |
| 1,6 | 5 | 10 | *Salix alba* | *Salix* | BROW | 0-10 |
| 1,5 | 5 | 10 | *Salix alba* | *Salix* | BROW | 0-10 |
| 1,2 | 5 | 10 | *Salix alba* | *Salix* | BROW | 0-10 |
| 1,3 | 5 | 10 | *Salix alba* | *Salix* | BROW | 0-10 |
| 1,6 | 5 | 10 | *Salix alba* | *Salix* | BROW | 0-10 |
| 1,5 | 5 | 10 | *Salix alba* | *Salix* | BROW | 0-10 |
| 1,2 | 5 | 10 | *Salix alba* | *Salix* | BROW | 0-10 |
| 1,2 | 5 | 10 | *Salix alba* | *Salix* | BROW | 0-10 |
| 1,4 | 5 | 10 | *Salix alba* | *Salix* | BROW | 0-10 |
| 1,7 | 5 | 10 | *Salix alba* | *Salix* | BROW | 0-10 |
| 1,8 | 5 | 10 | *Salix alba* | *Salix* | BROW | 0-10 |
| 1,9 | 5 | 10 | *Salix alba* | *Salix* | BROW | 0-10 |
| 1,5 | 5 | 10 | *Salix alba* | *Salix* | BROW | 0-10 |
| 1,5 | 5 | 10 | *Salix alba* | *Salix* | BROW | 0-10 |
| 1,6 | 5 | 10 | *Salix alba* | *Salix* | BROW | 0-10 |
| 1,7 | 5 | 10 | *Salix alba* | *Salix* | BROW | 0-10 |
| 1,4 | 5 | 10 | *Salix alba* | *Salix* | BROW | 0-10 |
| 1,3 | 5 | 10 | *Salix alba* | *Salix* | BROW | 0-10 |
| 1,5 | 5 | 10 | *Salix alba* | *Salix* | BROW | 0-10 |
| 1,6 | 5 | 10 | *Salix alba* | *Salix* | BROW | 0-10 |
| 1,4 | 5 | 10 | *Salix alba* | *Salix* | BROW | 0-10 |
| 1,7 | 5 | 10 | *Salix alba* | *Salix* | BROW | 0-10 |
| 1,7 | 5 | 10 | *Salix alba* | *Salix* | BROW | 0-10 |
| 1,8 | 5 | 10 | *Salix alba* | *Salix* | BROW | 0-10 |
| 1,8 | 5 | 10 | *Salix alba* | *Salix* | BROW | 0-10 |
| 1,1 | 5 | 10 | *Salix alba* | *Salix* | BROW | 0-10 |
| 1,2 | 5 | 10 | *Salix alba* | *Salix* | BROW | 0-10 |
| 1,3 | 5 | 10 | *Salix alba* | *Salix* | BROW | 0-10 |
| 1,4 | 5 | 10 | *Salix alba* | *Salix* | BROW | 0-10 |
| 1,3 | 5 | 10 | *Salix alba* | *Salix* | BROW | 0-10 |
| 1,4 | 5 | 10 | *Salix alba* | *Salix* | BROW | 0-10 |
| 1,5 | 5 | 10 | *Salix alba* | *Salix* | BROW | 0-10 |
| 1,5 | 5 | 10 | *Salix alba* | *Salix* | BROW | 0-10 |
| 1,5 | 5 | 10 | *Salix alba* | *Salix* | BROW | 0-10 |
| 1,3 | 5 | 10 | *Salix alba* | *Salix* | BROW | 0-10 |
| 1,3 | 5 | 10 | *Salix alba* | *Salix* | BROW | 0-10 |
| 1,6 | 5 | 10 | *Salix alba* | *Salix* | BROW | 0-10 |
| 1,7 | 5 | 10 | *Salix alba* | *Salix* | BROW | 0-10 |
| 1,7 | 5 | 10 | *Salix alba* | *Salix* | BROW | 0-10 |
| 1,7 | 5 | 10 | *Salix alba* | *Salix* | BROW | 0-10 |
| 1,8 | 5 | 10 | *Salix alba* | *Salix* | BROW | 0-10 |
| 1,7 | 5 | 10 | *Salix alba* | *Salix* | BROW | 0-10 |
| 1,9 | 5 | 10 | *Salix alba* | *Salix* | BROW | 0-10 |
| 1,6 | 5 | 10 | *Salix alba* | *Salix* | BROW | 0-10 |
| 1,5 | 5 | 10 | *Salix alba* | *Salix* | BROW | 0-10 |
| 1,2 | 5 | 10 | *Salix alba* | *Salix* | BROW | 0-10 |
| 1,3 | 5 | 10 | *Salix alba* | *Salix* | BROW | 0-10 |
| 1,6 | 5 | 10 | *Salix alba* | *Salix* | BROW | 0-10 |
| 1,7 | 5 | 10 | *Salix alba* | *Salix* | BROW | 0-10 |
| 1,8 | 5 | 10 | *Salix alba* | *Salix* | BROW | 0-10 |
| 1,9 | 5 | 10 | *Salix alba* | *Salix* | BROW | 0-10 |
| 1,4 | 5 | 10 | *Salix alba* | *Salix* | BROW | 0-10 |
| 1,4 | 5 | 10 | *Salix alba* | *Salix* | BROW | 0-10 |
| 1,4 | 5 | 10 | *Salix alba* | *Salix* | BROW | 0-10 |
| 1,6 | 5 | 10 | *Salix alba* | *Salix* | BROW | 0-10 |
| 1,6 | 5 | 10 | *Salix alba* | *Salix* | BROW | 0-10 |
| 1,8 | 5 | 10 | *Salix alba* | *Salix* | BROW | 0-10 |
| 1,5 | 5 | 10 | *Salix alba* | *Salix* | BROW | 0-10 |
| 1,5 | 5 | 10 | *Salix alba* | *Salix* | BROW | 0-10 |
| 1,4 | 5 | 10 | *Salix alba* | *Salix* | BROW | 0-10 |
| 1,6 | 5 | 10 | *Salix alba* | *Salix* | BROW | 0-10 |
| 1,9 | 5 | 10 | *Salix alba* | *Salix* | BROW | 0-10 |
| 1,8 | 5 | 10 | *Salix alba* | *Salix* | BROW | 0-10 |
| 1,7 | 5 | 10 | *Salix alba* | *Salix* | BROW | 0-10 |
| 1,5 | 5 | 10 | *Salix alba* | *Salix* | BROW | 0-10 |
| 1,2 | 5 | 10 | *Salix alba* | *Salix* | BROW | 0-10 |
| 1,3 | 5 | 10 | *Salix alba* | *Salix* | BROW | 0-10 |
| 1,2 | 5 | 10 | *Salix alba* | *Salix* | BROW | 0-10 |
| 1,3 | 5 | 10 | *Salix alba* | *Salix* | BROW | 0-10 |
| 1,4 | 5 | 10 | *Salix alba* | *Salix* | BROW | 0-10 |
| 1,3 | 5 | 10 | *Salix alba* | *Salix* | BROW | 0-10 |
| 1,4 | 5 | 10 | *Salix alba* | *Salix* | BROW | 0-10 |
| 1,5 | 5 | 10 | *Salix alba* | *Salix* | BROW | 0-10 |
| 1,5 | 5 | 10 | *Salix alba* | *Salix* | BROW | 0-10 |
| 1,7 | 5 | 10 | *Salix alba* | *Salix* | BROW | 0-10 |
| 1,7 | 5 | 10 | *Salix alba* | *Salix* | BROW | 0-10 |
| 1,8 | 5 | 10 | *Salix alba* | *Salix* | BROW | 0-10 |
| 1,7 | 5 | 10 | *Salix alba* | *Salix* | BROW | 0-10 |
| 1,5 | 5 | 10 | *Salix alba* | *Salix* | BROW | 0-10 |
| 1,5 | 5 | 10 | *Salix alba* | *Salix* | BROW | 0-10 |
| 1,5 | 5 | 10 | *Salix alba* | *Salix* | BROW | 0-10 |
| 1,5 | 5 | 10 | *Salix alba* | *Salix* | BROW | 0-10 |
| 1,5 | 5 | 10 | *Salix alba* | *Salix* | BROW | 0-10 |
| 1,5 | 5 | 10 | *Salix alba* | *Salix* | BROW | 0-10 |
| 24,7 | 5 | 10 | *Acer platanoides* | *Acer* | BROW | 0-10 |
| 23,1 | 5 | 10 | *Acer platanoides* | *Acer* | BROW | 0-10 |
| 2,3 | 5 | 10 | *Acer negundo* | *Acer* | BROW | 0-10 |
| 2,2 | 5 | 10 | *Acer negundo* | *Acer* | BROW | 0-10 |
| 2,2 | 5 | 10 | *Acer negundo* | *Acer* | BROW | 0-10 |
| 2,3 | 5 | 10 | *Acer negundo* | *Acer* | BROW | 0-10 |
| 5,6 | 5 | 10 | *Acer negundo* | *Acer* | UNBROW | 0-10 |
| 1,2 | 5 | 10 | *Salix alba* | *Salix* | UNBROW | 0-10 |
| 1,4 | 5 | 10 | *Salix alba* | *Salix* | UNBROW | 0-10 |
| 1,3 | 5 | 10 | *Salix alba* | *Salix* | UNBROW | 0-10 |
| 1,2 | 5 | 10 | *Salix alba* | *Salix* | BROW | 0-10 |
| 1,2 | 5 | 10 | *Salix alba* | *Salix* | BROW | 0-10 |
| 35,4 | 5 | 10 | *Picea abies* | Others | UNBROW | 0-10 |
| 33,1 | 5 | 10 | *Picea abies* | Others | UNBROW | 0-10 |
| 30 | 5 | 10 | *Abies alba* | Others | UNBROW | 0-10 |
| 30,9 | 5 | 10 | *Abies alba* | Others | UNBROW | 0-10 |
| 24,7 | 5 | 10 | *Picea abies* | Others | UNBROW | 0-10 |
| 67,8 | 5 | 10 | *Populus nigra* | *Populus* | UNBROW | 0-10 |
| 30,5 | 6 | 12 | *Acer platanoides* | *Acer* | UNBROW | 0-10 |
| 23,3 | 6 | 12 | *Picea abies* | Others | UNBROW | 0-10 |
| 20,7 | 6 | 12 | *Picea abies* | Others | UNBROW | 0-10 |
| 51,4 | 6 | 12 | *Populus alba* | *Populus* | UNBROW | 11-20 |
| 49,8 | 6 | 12 | *Populus alba* | *Populus* | UNBROW | 11-20 |
| 47,1 | 6 | 12 | *Populus alba* | *Populus* | UNBROW | 11-20 |
| 21 | 6 | 12 | *Acer platanoides* | *Acer* | UNBROW | 11-20 |
| 24,5 | 6 | 12 | *Acer pseudoplatanus* | *Acer* | UNBROW | 11-20 |
| 9,8 | 6 | 12 | *Juniperus communis* | Others | UNBROW | 11-20 |
| 3,7 | 6 | 12 | *Juniperus communis* | Others | UNBROW | 11-20 |
| 3,2 | 6 | 12 | *Juniperus communis* | Others | UNBROW | 11-20 |
| 20,5 | 6 | 12 | *Acer pseudoplatanus* | *Acer* | UNBROW | 11-20 |
| 7,8 | 6 | 12 | *Thuja* sp. | Others | UNBROW | 11-20 |
| 7,9 | 6 | 12 | *Thuja* sp. | Others | UNBROW | 11-20 |
| 8,2 | 6 | 12 | *Thuja* sp. | Others | UNBROW | 11-20 |
| 25,6 | 6 | 12 | *Acer pseudoplatanus* | *Acer* | UNBROW | 11-20 |
| 26 | 6 | 12 | *Acer platanoides* | *Acer* | UNBROW | 11-20 |
| 2,5 | 6 | 12 | *Juniperus communis* | Others | UNBROW | 21-30 |
| 2,9 | 6 | 12 | *Juniperus communis* | Others | UNBROW | 21-30 |
| 3,4 | 6 | 12 | *Juniperus communis* | Others | UNBROW | 21-30 |
| 21,2 | 6 | 12 | *Acer platanoides* | *Acer* | UNBROW | 21-30 |
| 20,2 | 6 | 12 | *Acer platanoides* | *Acer* | UNBROW | 21-30 |
| 62,4 | 6 | 12 | *Populus* x *canadensis* | *Populus* | UNBROW | 21-30 |
| 62,9 | 6 | 12 | *Populus* x *canadensis* | *Populus* | UNBROW | 21-30 |
| 63,1 | 6 | 12 | *Populus* x *canadensis* | *Populus* | UNBROW | 21-30 |
| 80 | 6 | 12 | *Populus* x *canadensis* | *Populus* | UNBROW | 21-30 |
| 24,1 | 6 | 12 | *Acer pseudoplatanus* | *Acer* | UNBROW | 21-30 |
| 4,7 | 6 | 12 | *Thuja* sp. | Others | UNBROW | 21-30 |
| 8,9 | 6 | 12 | *Thuja* sp. | Others | UNBROW | 21-30 |
| 8,7 | 6 | 12 | *Thuja* sp. | Others | UNBROW | 21-30 |
| 80 | 6 | 12 | *Populus* x *canadensis* | *Populus* | UNBROW | 21-30 |
| 15,7 | 6 | 12 | *Acer platanoides* | *Acer* | UNBROW | 21-30 |
| 16,9 | 6 | 12 | *Acer platanoides* | *Acer* | UNBROW | 21-30 |
| 10,3 | 6 | 12 | *Acer platanoides* | *Acer* | UNBROW | 21-30 |
| 10,4 | 6 | 12 | *Acer platanoides* | *Acer* | UNBROW | 21-30 |
| 34,5 | 6 | 12 | *Picea abies* | Others | UNBROW | 21-30 |
| 34 | 6 | 12 | *Picea abies* | Others | UNBROW | 21-30 |
| 29,2 | 6 | 12 | *Picea abies* | Others | UNBROW | 21-30 |
| 10,4 | 6 | 12 | *Acer negundo* | *Acer* | UNBROW | 21-30 |
| 9,7 | 6 | 12 | *Acer negundo* | *Acer* | UNBROW | 21-30 |
| 9,6 | 6 | 12 | *Acer negundo* | *Acer* | UNBROW | 21-30 |
| 2,3 | 6 | 12 | *Acer negundo* | *Acer* | UNBROW | 21-30 |
| 2,4 | 6 | 12 | *Acer negundo* | *Acer* | UNBROW | 21-30 |
| 1,7 | 6 | 12 | *Acer negundo* | *Acer* | BROW | 0-10 |
| 1,8 | 6 | 12 | *Acer negundo* | *Acer* | BROW | 0-10 |
| 1,8 | 6 | 12 | *Acer negundo* | *Acer* | BROW | 0-10 |
| 1,1 | 6 | 12 | *Acer negundo* | *Acer* | BROW | 0-10 |
| 1,2 | 6 | 12 | *Acer negundo* | *Acer* | BROW | 0-10 |
| 1,3 | 6 | 12 | *Acer negundo* | *Acer* | BROW | 0-10 |
| 1,4 | 6 | 12 | *Acer negundo* | *Acer* | BROW | 0-10 |
| 1,3 | 6 | 12 | *Acer negundo* | *Acer* | BROW | 0-10 |
| 1,4 | 6 | 12 | *Acer negundo* | *Acer* | BROW | 0-10 |
| 1,5 | 6 | 12 | *Acer negundo* | *Acer* | BROW | 0-10 |
| 1,5 | 6 | 12 | *Acer negundo* | *Acer* | BROW | 0-10 |
| 1,5 | 6 | 12 | *Acer negundo* | *Acer* | BROW | 0-10 |
| 1,3 | 6 | 12 | *Acer negundo* | *Acer* | BROW | 0-10 |
| 1,3 | 6 | 12 | *Acer negundo* | *Acer* | BROW | 0-10 |
| 1,6 | 6 | 12 | *Acer negundo* | *Acer* | BROW | 0-10 |
| 1,7 | 6 | 12 | *Acer negundo* | *Acer* | BROW | 0-10 |
| 1,7 | 6 | 12 | *Acer negundo* | *Acer* | BROW | 0-10 |
| 1,7 | 6 | 12 | *Acer negundo* | *Acer* | BROW | 0-10 |
| 1,8 | 6 | 12 | *Acer negundo* | *Acer* | BROW | 0-10 |
| 1,7 | 6 | 12 | *Acer negundo* | *Acer* | BROW | 0-10 |
| 1,9 | 6 | 12 | *Acer negundo* | *Acer* | BROW | 0-10 |
| 1,6 | 6 | 12 | *Acer negundo* | *Acer* | BROW | 0-10 |
| 1,5 | 6 | 12 | *Acer negundo* | *Acer* | BROW | 0-10 |
| 1,2 | 6 | 12 | *Acer negundo* | *Acer* | BROW | 0-10 |
| 1,3 | 6 | 12 | *Acer negundo* | *Acer* | BROW | 0-10 |
| 1,6 | 6 | 12 | *Acer negundo* | *Acer* | BROW | 0-10 |
| 1,5 | 6 | 12 | *Acer negundo* | *Acer* | BROW | 0-10 |
| 1,2 | 6 | 12 | *Acer negundo* | *Acer* | BROW | 0-10 |
| 1,2 | 6 | 12 | *Acer negundo* | *Acer* | BROW | 0-10 |
| 1,4 | 6 | 12 | *Acer negundo* | *Acer* | BROW | 0-10 |
| 1,7 | 6 | 12 | *Acer negundo* | *Acer* | BROW | 0-10 |
| 1,8 | 6 | 12 | *Acer negundo* | *Acer* | BROW | 0-10 |
| 1,9 | 6 | 12 | *Acer negundo* | *Acer* | BROW | 0-10 |
| 1,5 | 6 | 12 | *Acer negundo* | *Acer* | BROW | 0-10 |
| 1,5 | 6 | 12 | *Acer negundo* | *Acer* | BROW | 0-10 |
| 1,6 | 6 | 12 | *Acer negundo* | *Acer* | BROW | 0-10 |
| 1,7 | 6 | 12 | *Acer negundo* | *Acer* | BROW | 0-10 |
| 1,4 | 6 | 12 | *Acer negundo* | *Acer* | BROW | 0-10 |
| 1,3 | 6 | 12 | *Acer negundo* | *Acer* | BROW | 0-10 |
| 1,5 | 6 | 12 | *Acer negundo* | *Acer* | BROW | 0-10 |
| 1,6 | 6 | 12 | *Acer negundo* | *Acer* | BROW | 0-10 |
| 1,4 | 6 | 12 | *Acer negundo* | *Acer* | BROW | 0-10 |
| 1,7 | 6 | 12 | *Acer negundo* | *Acer* | BROW | 0-10 |
| 1,7 | 6 | 12 | *Acer negundo* | *Acer* | BROW | 0-10 |
| 1,8 | 6 | 12 | *Acer negundo* | *Acer* | BROW | 0-10 |
| 1,8 | 6 | 12 | *Acer negundo* | *Acer* | BROW | 0-10 |
| 1,1 | 6 | 12 | *Acer negundo* | *Acer* | BROW | 0-10 |
| 1,2 | 6 | 12 | *Acer negundo* | *Acer* | BROW | 0-10 |
| 1,3 | 6 | 12 | *Acer negundo* | *Acer* | BROW | 0-10 |
| 1,4 | 6 | 12 | *Acer negundo* | *Acer* | BROW | 0-10 |
| 1,3 | 6 | 12 | *Acer negundo* | *Acer* | BROW | 0-10 |
| 1,4 | 6 | 12 | *Acer negundo* | *Acer* | BROW | 0-10 |
| 1,5 | 6 | 12 | *Acer negundo* | *Acer* | BROW | 0-10 |
| 1,5 | 6 | 12 | *Acer negundo* | *Acer* | BROW | 0-10 |
| 1,5 | 6 | 12 | *Acer negundo* | *Acer* | BROW | 0-10 |
| 1,3 | 6 | 12 | *Acer negundo* | *Acer* | BROW | 0-10 |
| 1,3 | 6 | 12 | *Acer negundo* | *Acer* | BROW | 0-10 |
| 1,6 | 6 | 12 | *Acer negundo* | *Acer* | BROW | 0-10 |
| 1,7 | 6 | 12 | *Acer negundo* | *Acer* | BROW | 0-10 |
| 1,7 | 6 | 12 | *Acer negundo* | *Acer* | BROW | 0-10 |
| 1,7 | 6 | 12 | *Acer negundo* | *Acer* | BROW | 0-10 |
| 1,8 | 6 | 12 | *Acer negundo* | *Acer* | BROW | 0-10 |
| 1,7 | 6 | 12 | *Acer negundo* | *Acer* | BROW | 0-10 |
| 1,9 | 6 | 12 | *Acer negundo* | *Acer* | BROW | 0-10 |
| 1,6 | 6 | 12 | *Acer negundo* | *Acer* | BROW | 0-10 |
| 1,5 | 6 | 12 | *Acer negundo* | *Acer* | BROW | 0-10 |
| 1,2 | 6 | 12 | *Acer negundo* | *Acer* | BROW | 0-10 |
| 1,3 | 6 | 12 | *Acer negundo* | *Acer* | BROW | 0-10 |
| 1,6 | 6 | 12 | *Acer negundo* | *Acer* | BROW | 0-10 |
| 1,7 | 6 | 12 | *Acer negundo* | *Acer* | BROW | 0-10 |
| 1,8 | 6 | 12 | *Acer negundo* | *Acer* | BROW | 0-10 |
| 1,9 | 6 | 12 | *Acer negundo* | *Acer* | BROW | 0-10 |
| 1,5 | 6 | 12 | *Acer negundo* | *Acer* | BROW | 0-10 |
| 1,2 | 6 | 12 | *Acer negundo* | *Acer* | BROW | 0-10 |
| 1,3 | 6 | 12 | *Acer negundo* | *Acer* | BROW | 0-10 |
| 1,6 | 6 | 12 | *Acer negundo* | *Acer* | BROW | 0-10 |
| 1,5 | 6 | 12 | *Acer negundo* | *Acer* | BROW | 0-10 |
| 1,5 | 6 | 12 | *Acer negundo* | *Acer* | BROW | 0-10 |
| 1,5 | 6 | 12 | *Acer negundo* | *Acer* | BROW | 0-10 |
| 1,7 | 6 | 12 | *Acer negundo* | *Acer* | BROW | 0-10 |
| 1,8 | 6 | 12 | *Acer negundo* | *Acer* | BROW | 0-10 |
| 1,7 | 6 | 12 | *Acer negundo* | *Acer* | BROW | 0-10 |
