# Supplementary material for: Eurasian Beaver (Castor fiber) Winter Foraging Preferences in Northern Poland—The Role of Woody Vegetation Composition and Anthropopression Level
Source: Animals (Basel). 2020 Aug 8;10(8):1376. doi: 10.3390/ani10081376 (PMC7460282; doi:10.3390/ani10081376)
Supplement: Supplementary file 1 [file animals-10-01376-s001.zip › Table S2.docx]

**Table S2.** Electivity index values for woody plants inventoried in each transect located along the Gwda River in three diameter classes for selected woody plants.

| Transect number | Shoot diameter [cm] | *Acer* (introduced) | *Acer* (native) | *Alnus* | *Crataegus* | *Pinus* | *Populus* | *Prunus* | *Quercus* | *Salix* | *Sambucus* | Others | Total |
| --- | --- | --- | --- | --- | --- | --- | --- | --- | --- | --- | --- | --- | --- |
|  |  | **index value** | | | | | | | | | | | |
| I | <2.5 | - | - | 0.47 | - | - | - | 1.61 | - | - | - | - | 1.03 |
|  | 2.5-10 | - | - | 0.09 | 0.06 | - | - | 1.85 | - | - | -0.62 | - | 0.28 |
|  | >10 | - | - | - | 0.80 | - | 1.69 | - | - | - | - | 3.51 | -1.33 |
|  | total | - | - | -0.76 | 0.03 | - | 0.56 | 2.04 | - | - | -1.07 | 2.01 | - |
| II | <2.5 | - | 1.11 | 1.02 | -0.04 | - | -1.44 | 0.24 | - | - | - | -0.71 | 0.37 |
|  | 2.5-10 | - | - | 0.42 | -0.54 | -0.86 | - | 0.44 | - | - | - | - | 0.17 |
|  | >10 | - | - | 0.86 | - | - | -1.97 | - | - | - | - | - | -1.10 |
|  | total | - | 0.92 | 0.65 | 0.40 | -1.56 | -0.57 | 0.52 | - | - | - | -0.74 | - |
| III | <2.5 | - | 0.05 | -0.20 | - | - | - | - | - | 0.15 | -0.29 | - | 1.49 |
|  | 2.5-10 | - | - | - | - | - | - | - | - | 1.75 | 0.77 | 0.15 | -1.85 |
|  | >10 | 1.10 | - | - | 0.25 | - | - | - | - | - | 0.92 | - | -0.78 |
|  | total | -0.55 | 0.12 | -0.41 | -2.51 | - | - | - | - | 0.39 | -0.72 | -2.17 | - |
| IV | <2.5 | 0.00 | - | -0.29 | - | - | - | - | - | 0.01 | - | - | 1.65 |
|  | 2.5-10 | 1.63 | - | 1.16 | - | - | - | - | - | - | -2.47 | 1.10 | -1.40 |
|  | >10 | - | 2.27 | - | - | - | -0.25 | - | - | - | - | - | -1.92 |
|  | total | 0.23 | 0.16 | -1.18 | - | - | -2.06 | - | - | 0.38 | -3.16 | -0.83 | - |
| V | <2.5 | -0.09 | - | - | - | - | - | - | - | 0.35 | - | - | 1.31 |
|  | 2.5-10 | 0.38 | - | -1.83 | 1.10 | - | - | - | 0.51 | - | 0.26 | 1.10 | -0.86 |
|  | >10 | - | 3.43 | -1.21 | - | - | - | - | - | - | - | - | -1.77 |
|  | total | 0.03 | 0.28 | -2.57 | 0.28 | - | - | - | -0.43 | 1.11 | -0.62 | -1.53 | - |
| VI | <2.5 | 100% of browsed plants in this transect were classified as *Acer negundo* (introduced species) with diameter below 2.5 cm | | | | | | | | | | | |
|  | 2.5-10 |  |  |  |  |  |  |  |  |  |  |  |  |
|  | >10 |  |  |  |  |  |  |  |  |  |  |  |  |
|  | total |  |  |  |  |  |  |  |  |  |  |  |  |

dashes in cells with no plants in the diameter class or belonging to the genus
